# Critical factors influencing cost estimators’ judgements on cost contingencies in highway construction projects: An empirical study in the UK

**DOI:** 10.1371/journal.pone.0314665

**Published:** 2024-12-16

**Authors:** Lilin Zhao, Jinpeng Wang, Shang Zhang

**Affiliations:** 1 School of Transportation and Civil Engineering, Nantong University, Nantong, China; 2 Department of Construction Management, Suzhou University of Science and Technology, Suzhou, China; Atlantic Technological University, IRELAND

## Abstract

Unforeseen additional costs are major sources of cost overruns for the UK’s highway projects. General contractors normally allocate cost contingencies in their tender prices to cover these costs, primarily based on the judgements of their cost estimators. However, cost contingencies allocated to the same risk by different cost estimators can vary significantly. Although objective factors influencing the estimation of cost contingencies have been extensively investigated, subjective factors remain underexplored. Therefore, this paper aims to address this gap by investigating the subjective factors influencing cost estimators’ judgements through a qualitative study. Twelve semi-structured interviews were conducted with experienced cost estimators working for general contractors in the UK’s construction industry. A follow-up focus group study was also conducted with seven experienced cost estimators in the UK’s construction industry to validate the interview results. The findings reveal that there are thirteen influencing factors affecting cost estimators’ judgements on cost contingencies in highway construction projects. Seven of them, namely cost estimators’ ability to effectively communicate with stakeholders, self-reflection on previous practice, open-mindedness to project uniqueness, previous project experiences, risk attitude, assumption of the controllability of risks, and perceived quality of tender documents were identified as critical ones. They were grouped into two categories, including expertise-related factors and personal factors. The findings contribute to the lack of research on the subjective factors influencing cost estimators’ judgement-making process in highway construction projects in developed countries. The results also provide new practical insights for general contractors to develop more focused strategies and training programs to enhance the reliability of cost estimators’ judgements on cost contingencies, which can effectively reduce the probability of cost overruns.

## Introduction

Infrastructure has contributed significantly to the development of the UK’s economy and society [1]. Highways are characterised as one of the most important infrastructures in the UK, which carry two-thirds of all freight traffic, providing necessary road networks for numerous businesses (e.g., construction, logistics, engineering and raw materials) [2]. However, despite that large budget allocations have been designated to spend on the construction of highway projects in the UK, cost overruns are still a prevalent problem, which impedes the successful delivery of highway projects within budget [1]. For instance, according to [3], the actual cost spent on the development of the UK’s highway system from 2020–2025 was estimated to exceed its budgeted cost by approximately 31%. Because developing a new highway construction project involves huge capital investment and human resources, cost overruns can have significant adverse impacts on the economy of a country [4], which can further cause serious consequences among taxpayers and shareholders and even lead to political destabilization of the incumbent party [5].

Unforeseen costs caused by residual risks that remain after applying all risk mitigation strategies have been identified as the primary source of cost overruns [6,7]. The most common practice for general contractors to cover these costs to avoid cost overruns is to include cost contingencies in their tender price to the owner [8]. Since highway construction projects normally have high capital investment, complex longitudinal underground conditions [9], wide geographical distribution [10], and long delivery time [11], the accurate estimation of the cost contingencies is challenging, which can lead to frequent cost overruns and significant project failure. More importantly, most general contractors tend to use deterministic methods that highly rely on expert judgement, especially the judgements of cost estimators in their organizations, to determine the cost contingencies, which is criticized as subjective, imprecise, and difficult to justify or defend [7]. Therefore, it is necessary to understand the underlying factors that affect cost estimators’ judgements on cost contingencies.

In terms of identifying factors influencing professionals’ judgements on cost contingencies in the construction industry, previous studies have primarily focused on examining objective factors, such as those related to project characteristics (e.g., [6,12]), policies, economics, and technologies (e.g., [13,14]). However, there remains a significant gap in research focusing on subjective factors. It is essential to understand subjective factors influencing cost estimators’ judgements since under the same conditions, the risk ratings assigned to risks by different cost estimators may vary significantly [15], resulting in diverse cost contingencies allocated and thus increasing the likelihood of cost overruns. For instance, a case study of two UK civil engineering contractors by [7] found that insufficient attention to cost estimators’ risk perception and intuition leads to significant variations in allocated contingencies, resulting in cost overruns. Understanding these subjective factors is crucial for general contractors, as it influences their interpretation of cost contingencies allocated by cost estimators and thus the amount of contingencies incorporated into their tender prices to reduce cost overruns [7]. Furthermore, while some studies have explored subjective factors influencing professionals’ risk judgements in the construction industry, they have predominantly examined these factors from the perspectives of company directors or project managers (e.g., [6,16]). There is a lack of research investigating the judgements of cost estimators within the contractor organizations in highway projects. It is important to understand the factors influencing the judgement of cost estimators, as professionals in different positions within an estimating team fulfil various roles and evaluate risks from distinct perspectives. For instance, compared to senior directors who adopt a commercial viewpoint, cost estimators concentrate more on risk-specific details and are less susceptible to the influence of external project factors, such as market conditions [17]. Therefore, factors which are considered influential by other professionals may be less important from the perspective of cost estimators. Additionally, cost estimators play a pivotal role in predicting the tender prices, including costs of materials, labor and equipment, management fees, and profits [18]. They are recognized as important professionals in this field, particularly when it comes to assessing and judging cost contingencies in tender prices, as their judgements enable senior management team to make informed decisions on cost contingencies to win the contract with reasonable profits [17]. Therefore, to address the research gap, this paper aims to examine critical subjective factors influencing cost estimators’ judgements on cost contingencies in general contractors’ tender prices for highway construction projects. This research specifically focuses on small highway construction projects due to the observed higher average cost overruns in comparison to larger projects [19]. Additionally, general contractors place greater reliance on their cost estimators’ judgements when evaluating cost contingencies for small construction projects [7].

The findings are important for general contractors to develop more targeted strategies to enhance the accuracy of cost estimators’ judgements. This will improve the competitiveness of their tender prices in addition to reduce the probability of cost overruns in highway construction projects.

### Literature review

Objective factors influencing contractors’ cost contingencies in construction projects. Factors influencing contractors’ cost contingencies in construction projects in current research can be broadly categorized into endogenous and exogenous factors. Endogenous factors are related to specific project characteristics, such as size, design quality, and type of procurement [20]. For instance, [6], through a questionnaire survey among construction contractors and university professors in the Gaza Strip, identified incompatible designs as a significant factor influencing cost contingencies [12], by analysing quantitative data on project characteristics from historical records and qualitative data collected from transportation professionals via opinion survey, discovered that changes requested by owners and inaccurate structural design as the two factors that have significantly positive impact on contractors’ cost contingencies in highway projects.

Exogenous factors are external to projects but influence their costs, such as political, legal, social, and economic factors [20]. [6] identified 61 factors influencing cost contingencies and classified them into 12 groups, including environmental, legal, economic, and political factors. Among these, political factors group was found to have the most significant impact on cost contingencies. [14] also investigated the influence of economic, political, and technical factors on contractors’ cost contingencies by studying cases collected from the archive of a contractor in the Netherlands, identifying technical and political factors as having the greatest impact. [13] discovered that exogenous factors, particularly those related to the economy, policy, and technology, significantly influence estimators’ judgements on cost contingencies in Ghana construction industry.

### Subjective factors influencing cost estimators’ judgements on cost contingencies

Despite limited research specifically on subjective factors influencing cost estimators’ judgments on cost contingencies, many studies have explored subjective factors affecting professionals’ risk judgments in the construction industry. For instance, [21] proposed that professionals use heuristics when making judgements, which renders them susceptible to cognitive biases. [14] concurred, noting that cognitive biases, particularly optimism bias, significantly influence contractors’ judgements on cost contingencies in construction projects, consequently leading to cost overruns. [15] found that experts’ judgements on risk ratings are associated with their perceived controllability of risks and risk attitudes. Similarly, [16] observed the relationship between risk attitude and experts’ risk rating, adding that risk-seeking project managers tend to assign higher ratings to positive risks (i.e., opportunities) compared to their risk-adverse and risk-neutral counterparts.

In addition, expertise has been widely recognized as a significant characteristic of experts, contributing to their ability to make more reliable and accurate judgements compared to non-experts [22,23]. Numerous studies have explored the role of expertise in expert judgement across various fields, such as medicine (e.g., [22,24]), forensic accounting (e.g., [14,25]), and project management (e.g., [26,27]). Factors influencing professionals’ judgements in these research fields can offer valuable insights into understanding those in the construction industry. Building on a comprehensive review of relevant studies in these fields, this research further identified three expertise-related factors significantly influencing professionals’ judgements, namely self-reflection, the ability to interpret and adapt to unique situations, and virtue ethics.

As a result, a total of six subjective factors influencing professionals’ judgements on cost contingencies are identified and classified into two categories, namely personal factors and expertise-related factors, as shown in Table 1. These identified factors guided the design of the subsequent interview questions to enhance the relevance and comprehensiveness of data collection.

**Table 1:** Subjective factors influencing professionals’ judgements on cost contingencies.

Categories	Factors	Descriptions	References
Personal factors	Optimism bias	Professionals experiencing optimism bias tend to be overly optimism about key project parameters such as capital and operating costs, duration, and benefits delivery, often leading to an underestimation of cost contingencies.	[14,20]
	Perceived controllability of risks	Professionals, who believe they can control a risk, tend to underestimate its probability and impact, resulting in lower cost contingencies being allocated.	[15,16]
	Risk attitude	For the same risk scenario, a risk-seeking expert tends to assign a lower risk rating when compared with a risk-averse expert, leading to lower cost contingencies being allocated.	[15,16,28]
Expertise-related factors	Self-reflection	Experts, through ongoing self-reflection on their past experiences, can be aware of their limitations and be dedicated to finding ways to mitigate them. This enables them to make more accurate judgements in future projects.	[14,29]
	Ability to interpret and adapt to unique situations	By effectively understanding the specific characteristics of each project, experts can tailor their judgements to account for its uniqueness, thus enabling them to make more informed and accurate judgements.	[27,30]
	Virtue ethics	The ethical framework guides cost estimators to make judgements that are not only driven by personal gain or self-interest but also are aligned with the common good, which enhances the reliability of their judgements.	[14,22,27]

### Research methodology

**Fig 1:**
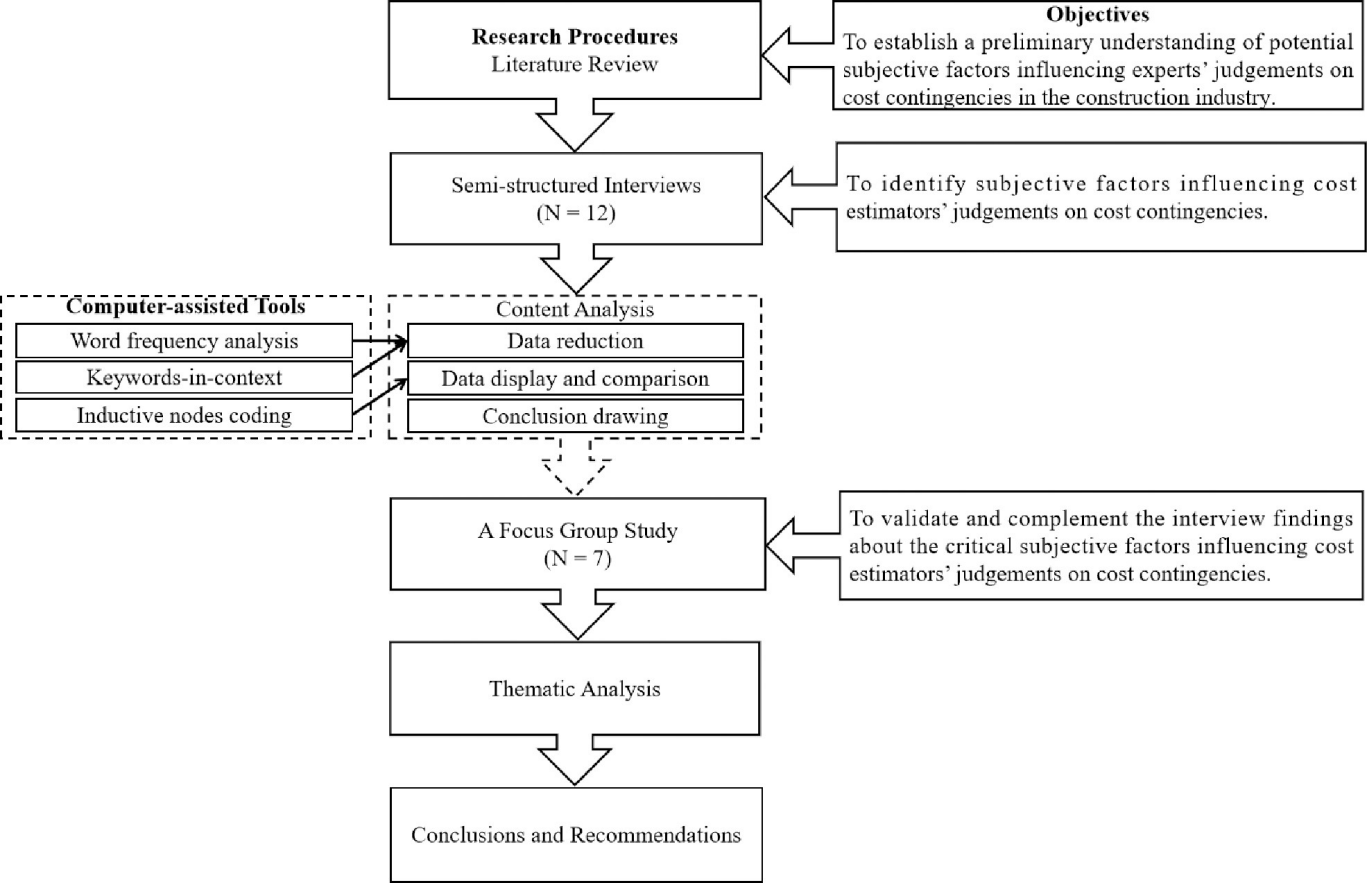
The research framework of this study.

As illustrated in Fig 1, an empirical qualitative research design was adopted in this research, which allowed in-depth, rich, and detailed data to be gathered [31]. The research started with a critical literature review to identify the potential subjective factors influencing cost estimators’ judgements on cost contingencies in the construction industry. This consolidates a strong basis for subsequent empirical studies, including extensive semi-structured interviews and a focus group study. Both fieldworks were carried out with experienced cost estimators online through *Teams* (Microsoft Corp., Redmond, WA, USA), following ethics approval from Loughborough University’s ethics committee and receipt of signed consent forms from the participants. Semi-structured interviews were chosen for their flexibility, allowing probing of key themes while still offering participants the freedom to elaborate on their experiences [32]. They were conducted to solicit and analyse practitioners’ perceptions and experiences about the influencing factors involved in cost estimators’ judgement-making process regarding cost contingencies. A focus group study was followed to validate and complement the interview findings. This approach has been successfully employed in existing studies in the construction industry. For instance, [33] identified critical risk factors and risk management measures for residential recycled-water schemes by conducting interviews with practitioners, which were validated through a focus group study and a detailed literature review.

### Semi-structured interviews

The interviews were conducted from May, 2021 to August, 2021. A total of twelve semi-structured interviews were conducted with cost estimators selected through purposive sampling (see Table 2 for sample details). To ensure the representativeness and validity of the data collected, interviewees were required to have at least three years of experience working as cost estimators for UK general contractors and extensive knowledge of assessing risks in highway construction projects. UK contractors are typically categorised into three tiers based on size, financial capacity, project scope, and specialization. Tier 1 contractors are large firms, capable of managing complex, high-value projects, typically with contract values exceeding £50 million. Tier 2 contractors normally handle mid-sized projects, with contract values ranging between £10 and £50 million, while Tier 3 contractors focus on smaller and more localized projects, generally valued below £10 million [34]. To enhance the comprehensiveness and generalisation of research results, interviewees were recruited from eight general contractors across tier 1 to 3 within the UK construction sector, with a maximum of three interviewees per contractor. The majority of the interviewees were from tier 1 and 2 contractors, as these companies typically handle larger and more complex projects where cost contingencies are critical and challenging to estimate.

**Table 2:** Interviewee profiles.

No.	Codes	Positions	Levels of contractors	Experience in risk assessment (years)	Experience in the construction industry (years)
1	A	Managing cost estimator	Tier 3	10–15	20–25
2	B	Chief cost estimator	Tier 2	20–25	> 30
3	C	Chief cost estimator	Tier 1	> 30	> 30
4	D	Senior cost estimator	Tier 1	0–5	10–15
5	E	Senior cost estimator	Tier 1	0–5	> 30
6	F	Managing cost estimator	Tier 1	10–15	10–15
7	G	Senior cost estimator	Tier 2	10–15	15–20
8	H	Chief cost estimator	Tier 1	> 30	> 30
9	I	Senior cost estimator	Tier 2	5–10	20–25
10	J	Senior cost estimator	Tier 2	> 30	> 30
11	K	Senior cost estimator	Tier 2	> 30	> 30
12	L	Senior cost estimator	Tier 2	> 30	> 30

The recruitment process began by contacting company directors to obtain permission to interview their estimators and to request recommendations for suitable candidates. Subsequently, the recommended estimators were directly emailed. Snowball sampling was also employed, asking current interviewees to recommend eligible estimators for future interviews [35]. There was no predetermined sample size and the interview process completed when the data reached saturation [36]. Data saturation, a widely accepted method for determining sample size in interview research, ensures that the data collected is rich and comprehensive enough to represent the diversity and complexity of the phenomenon being studied [37]. In this research, no new viewpoints about influencing factors emerged after the tenth interview, suggesting that the data were saturated. Two additional interviews were conducted to confirm that the data saturation was achieved.

The interview mainly comprised two sections. The first section aimed to collect the interviewees’ background information, including their professional backgrounds and years of working experience. The second section focused on identifying the potential factors that influence individual cost estimators’ judgements on cost contingencies. It commenced by asking the interviewees to elaborate how they judged the magnitude of cost contingencies in a recent or notable highway construction project. The purpose of this part was to elicit detailed responses by prompting the interviewees to reflect on a specific case that they could recall vividly, which can facilitate comprehensive answers to subsequent questions in relation to the factors involved in the judgement process. The subsequent questions were designated based on the literature review results. For instance, since risk attitude was identified as a significant factor influencing professionals’ risk judgements, the question “How can your personal attitude towards risks affect your judgement on cost contingencies?” was raised. Additionally, because this study employed semi-structured interviews, some spontaneous questions were also asked based on the interviewees’ responses, allowing for a more comprehensive and in-depth understanding of the subjective factors influencing cost estimators’ judgements on cost contingencies

Furthermore, throughout the interview process, the researcher consistently reminded the interviewees that this study focuses on individual cost estimators rather than the organizations they work for. This approach was pivotal in ensuring the validity of the collected data and achieving the research objectives.

In this research, an inductive content analysis approach was employed to qualitatively analyse the interview data using *NVivo 12* (QSR, Inc., Melbourne, Australia). The software’s built-in quantitative tools were leveraged for organising and coding qualitative data, aiming to mitigate bias and enhance the validity and reliability of the results [38]. Similar data analysis approach has been successfully employed by [39] in identifying performance indicators measuring the effectiveness of claim management in the construction management field. The content analysis approach consists of three steps, namely data reduction, data display and comparison, and conclusion drawing. First, all interviews were transcribed and imported into the *NVivo* platform. Then, word frequency analysis and keywords-in-context analysis were conducted in *NVivo 12* to achieve data reduction by focusing on relevant information aligned with the research objectives [40]. These tools are widely used in interview studies to count and highlight words that appear more frequently in transcripts [41]. Table 3 summarises the results of the word frequency analysis, listing the keywords in the interview data. The results show that "experience" was the most frequently mentioned word with a total of 200 times, accounting for 2.84% of the total word count, which is followed by "information", "team", "business", and "client". This indicates that the influencing factors involved in the cost estimators’ judgement process are mostly related to these topics, especially those in relation to cost estimators’ experiences. After that, the keywords-in-context method was employed to examine how these keywords were used by interviewees. This involved identifying the words and phrases that appear before and after the keywords. As a result, a collection of key words and phrases was generated, providing deeper insights into the contexts for the subsequent coding process. Second, inductive node coding was employed to codify the transcripts, analysing and comparing the interviewees’ responses regarding the influencing factors. The initial codes and categories were established based on the keywords and phrases identified through the keywords-in-context method, which served as a foundation for the overall coding process. Third, line-by-line coding was conducted on the transcripts to complement the assigned codes, categories, or themes. Through a comprehensive examination and comparison of the established codes and the factors identified in the literature review, the factors influencing cost estimators’ judgements were identified.

**Table 3:** Word frequency analysis results.

Rank	Statistical Index	Count	Weighted percentage (%)	Similar words
1	Experience	200	2.84	Past experience
2	Information	167	2.37	Documents, design
3	Team	145	2.06	Group
4	Business	115	1.64	Contractor
5	Client	96	1.37	Local authority, government
6	Knowledge	81	1.14	Understanding
7	Attitude to risks	63	0.95	Conservative person, pessimistic person
8	Control	57	0.81	Mitigation, controllability
9	Contract	47	0.67	Contracts
10	Directors	33	0.47	Director, senior level
11	Environment	30	0.43	Environmental issues, plant
12	Supply chain	28	0.4	Stakeholders, suppliers
13	Win	20	0.28	Competitive tender
14	Lessons learned	19	0.29	Feedback
15	Safety	14	0.2	Health and safety

### Focus group study

After analysing the interview data, a focus group study was conducted with seven experienced cost estimators from June, 2022 to September, 2022, including the recruitment process. This group comprised six new participants from two contractor companies and Interviewee H in Table 2 was included due to recruitment challenges and time constraints. All participants were recruited following the same criteria and procedures as the interview study (see Table 4 for sample details). The aim of the focus group study was to verify the external validity of the interview findings by capturing the collective views of the participants [42] towards the critical factors influencing cost estimators in judging cost contingencies. Compared to interviews, the focus group study provided a more accurate account of the phenomenon studied due to the researchers’ role as facilitators rather than investigators in the process [43]. This minimised the researchers’ bias and subjectivity in the data analysis process. In addition, the focus group study provided more in-depth and richer data than interviews by fostering constant interactions and in-depth discussions among participants who share similar professional backgrounds [44].

**Table 4:** Profiles of the focus group participants.

Codes	Positions	Levels of contractors	Experience ranges in risk assessment (years)	Experience ranges in the construction industry (years)
Participant 1	Head of cost estimation department	Tier 2	5–10	5–10
Participant 2	Senior cost estimator	Tier 1	5–10	15–20
Participant 3	Senior cost estimator	Tier 2	15–20	> 20
Participant 4	Senior cost estimator	Tier 2	15–20	> 20
Participant 5	Senior cost estimator	Tier 1	5–10	> 20
Participant 6	Senior cost estimator	Tier 1	> 20	> 20
Participant 7	Chief cost estimator	Tier 1	> 20	> 20

The focus group commenced with a warm-up talk, encouraging the professionals to actively engage in the forthcoming formal discussion. During the formal discussion, instead of directly presenting the interview results to the participants and asking for their comments, a set of open-ended questions were posed concerning the various factors influencing individual estimators’ judgements on cost contingencies. For instance, participants were asked about the reasons for cost estimators judging differently on cost contingencies for the same risk and the causes of their irrational judgements on cost contingencies. This approach improved the validity of the results by reducing the confirmation bias of the participants and observer-expectancy effects [45]. Participants were asked to individually write down their answers on the Whiteboard (*an auxiliary tool embedded in Teams*) and then explain the factors they had identified to the rest of the group. Throughout the explanation process, other focus group participants were encouraged to express dissenting opinions if they disagreed with the factors presented by a participant. These dissenting opinions triggered further in-depth discussions among the participants. In the absence of dissent, agreement was assumed by default. A factor was granted as an influencing factor in this research when all participants reached a unanimous agreement.

The discussion was recorded, transcribed, and analysed in four steps, using a hybrid of deductive and inductive thematic analysis approach via *NVivo* 12. First, the researchers thoroughly read the transcript multiple times to familiarise themselves with the data. Second, the researchers developed a coding framework with themes, categories, and codes predetermined based on the interview results, serving as a foundation for further data interpretation. Third, relevant data were organized under different codes, with some new codes emerging which were not included in the coding framework. Fourth, the researchers examined patterns in the data and classified them into the predetermined categories and themes. Some new categories emerged from the focus group data were also developed in this process. The factors identified in the interview findings were validated if they were also recognised by all the focus group participants. In general, the focus group results confirmed most of the interview findings, with a few differences observed (see Table 5). The detailed results are discussed in the following sections.

**Table 5:** Factors influencing cost estimators’ judgements on cost contingencies.

Factors	Interviewees													Focus group
	A	B	C	D	E	F	G	H	I	J	K	L	Total	
(1) Risk attitude	x	x			x	x	x	x	x	x	x	x	10	√
(2) Previous project experiences	x	x	x	x	x	x	x	x	x	x	x	x	12	√
(3) Past experiences with stakeholders	x		x										2	√
(4) Assumption of the controllability of risks	x	x	x	x	x		x	x	x		x		9	√
(5) Motivation to win a contract	x				x		x			x	x		5	
(6) Organisational culture			x		x			x					3	√
(7) Current workload	x												1	√
(8) Perceived quality of tender documents						x		x	x	x	x	x	6	√
(9) Perceived competition among general contractors	x						x				x		3	√
(10) Ability to extract relevant information from documents	x						x		x	x	x		5	√
(11) Ability to deconstruct risks		x				x			x	x	x	x	6	
(12) Ability to effectively communicate with stakeholders	x	x	x	x	x	x	x	x	x	x	x	x	12	√
(13) Open-mindedness to project uniqueness		x			x	x	x		x	x	x		7	√
(14) Self-reflection on previous practice	x	x	x	x	x	x	x	x	x	x	x	x	12	√
(15) Ability to contextualise risks	x	x	x	x	x		x	x	x	x	x	x	11	
(16) Empathy and sympathy		x	x			x		x	x	x		x	7	
(17) A strong sense of responsibility	x	x	x	x				x	x	x			7	
(18) Perceived senior managers’ attitude towards risks*													0	√

Note: “x” means that the factor was identified by interviewees as influencing cost estimators in judging cost contingencies in highway construction projects.

“√” means that the factor identified was unanimously agreed by all the focus group professionals as an influencing factor that affects cost estimators in judging cost contingencies in highway construction projects.

“*” means that the factor was emerged from the focus group study.

### Inclusivity in global research

Additional information regarding the ethical, cultural, and scientific considerations specific to inclusivity in global research is included in the Supporting Information.

## Results and discussions

Based on the results from the literature review and an inductive content analysis of the interview data, a total of seventeen factors were identified that influence cost estimators in judging cost contingencies. Among them, twelve factors were further validated through the focus group study. Additionally, a new influencing factor, namely *the perceived senior managers’ attitude towards risks*, was suggested by all the professionals in the focus group. Consequently, a total of thirteen factors are considered influencing factors in the judgement-making process of cost estimators for cost contingencies. The results are summarised in Table 5.

### Critical factors influencing cost estimators’ judgements on cost contingencies

Among the thirteen influencing factors, seven of them were further recognized as critical ones, which were determined based on the criteria of being recognized by at least half of the interviewees and unanimously agreed by all focus group participants [46]. The seven critical factors are ranked in order of their frequency of agreement by the interviewees. They are further classified into two categories according to the specific context of this study, namely expertise-related factors and personal factors, as shown in Table 6. The detailed elaborations on the seven critical influencing factors are provided in the following sections.

**Table 6:** Critical factors influencing cost estimators’ judgements on cost contingencies.

Categories	Factors	Rank
Expertise-related factors	Ability to effectively communicate with stakeholders	1
	Self-reflection on previous practice	1
	Open-mindedness to project uniqueness	6
Personal factors	Previous project experiences	1
	Risk attitude	4
	Assumption of the controllability of risks	5
	Perceived quality of tender documents	7

It is worth noting that the *ability to contextualise risks* was recognised as an influencing factor by 11 out of 12 interviewees in the semi-structured interviews but did not emerge as such in the focus group discussion. This discrepancy can be attributed to the influence of peers in the focus group setting [43], where dynamics and interactions among participants may have led to different prioritisations compared to free-talk individual interviews [47]. Specifically, Interviewee H, who participate in both the interview and the focus group, might have adjusted his opinion based on the new perspectives and insights shared by others during the group discussion. Additionally, the limited time and depth of focus group discussions might have constrained the exploration of certain factors, leading to a shift in perceived importance. As a result, the ability to contextualise risks is not recognised as a critical influencing factor in this research.

### Expertise-related factors

Expertise-related factors encompass the skills and personality traits of cost estimators driven by their knowledge. Among them, *self-reflection on previous practice* is commonly recognized as an expertise-related factor influencing expert judgement in existing studies (e.g., [48] in forensic accounting), while the remaining two factors identified in this research are new. These factors are derived from the special roles of cost estimators within the estimating team, such as coordinating among various stakeholders (e.g., clients, contractors) and providing context-specific estimations of the cost contingencies based on tender documents [19].

### Ability to effectively communicate with stakeholders

Cost estimators’ ability to effectively gather and balance other professionals’ (e.g., commercial managers, project managers) experiences and viewpoints regarding risks is an essential skill for improving the reliability of their judgements. This aligns with the view that effective communication is essential for professionals in the construction industry, as it facilitates knowledge transfer, allowing professionals to accumulate or acquire new knowledge from others [49]. Additionally, the UK government actively promotes public engagement in highway construction projects, facilitating opportunities for stakeholders and local communities (e.g., residents, businesses) to voice their concerns, offer insights, and actively contribute to the decision-making processes [50]. As a result, fostering effective collaboration among these stakeholders and local communities is crucial for achieving successful project outcomes in the context of highway construction projects in the UK [51]. Cost estimators play a critical role in this multidisciplinary collaboration as coordinators, who are required to develop strong communication skills to effectively assemble, consolidate, and reconcile diverse viewpoints from them to make reasonable judgements [19]. To improve cost estimators’ communication skills, the provision of training sessions and the promotion of technological instruments such as the gesture technique and keyword emphasisers are recommended for the general contractors [52]. Moreover, projects participants’ perceptions of risks can vary significantly depending on their specific positions and responsibilities. Hence, cost estimators should be equipped with the ability to comprehend and balance diverse viewpoints through effective communication and brainstorming. By gathering different perspectives, cost estimators can gain valuable insights and improve the reliability of judgements on cost contingencies.

- **Self-reflection on previous practice.** Self-reflection is a process of knowledge accumulation through learning facilitated by individuals’ awareness of their personal limitations and their determination to overcome them [53]. It is widely recognized as an essential skill for practitioners in project management, enabling them to develop concrete and context-dependent knowledge and capabilities to effectively address challenges in contemporary projects [54,55]. The interviewees and focus group professionals frequently engaged in reflective thinking by critically evaluating their previous highway project working experiences, identifying their areas for improvement, and actively taking actions to enhance cost estimating skills and knowledge. Given the rapidly evolving and multidisciplinary nature of the construction projects, it is essential for cost estimators to engage in continuous self-reflection to identify their development needs in cost estimating and update their knowledge accordingly to make informed adjustments in cost contingencies [56]. For instance, Geographic Information System (GIS) has emerged as a widely adopted tool in highway construction projects, which can provide valuable spatial analysis, geotechnical analysis, environmental impact assessment, and utility coordination capabilities to address the complex geological, long-distance, and multidisciplinary characteristics of such projects in the UK [57]. Hence, cost estimators are required to possess a comprehensive understanding of GIS to effectively assess terrain conditions, evaluate environmental factors, and identify potential risks and constraints in their judgement-making processes [58]. With effective self-reflection on previous practice, cost estimators can make more accurate judgements on cost contingencies. To foster positive self-reflection, the promotion of linguistic tools such as distanced self-talk and expressive writing as well as the adoption of an observer perspective are essential, which can allow cost estimators to step back from the emotional immediacy of a situation and get rid of negative emotions and intrusive thoughts to further improve the reliability of their judgements [59].

### Open-mindedness to project uniqueness

Open-mindedness refers to the personality trait exhibited by individuals who are receptive to new ideas and information, willing to consider them, and able to integrate them into their own perspectives without being influenced by preconceived notions or biases [60]. The interviewees and focus group professionals displayed a high level of open-mindedness when assessing cost contingencies in highway construction projects. They emphasized the importance of considering the unique characteristics of each highway project, rather than rigidly applying historical data of similar projects. Highway construction projects are characterized by their uniqueness, as they encounter higher regulatory requirements (e.g., resource-sufficient, cost-effective, environmentally-friendly), involve numerous stakeholders, have longer life span, and are influenced by more traffic restrictions [51,61]. Consequently, when judging cost contingencies for highway construction projects, it is crucial for cost estimators to be open to these unique aspects of each project and actively tailor the judgements to meet the specific requirements and challenges presented.

### Personal factors

Personal factors pertain to the individual characteristics and attributes of cost estimators. This category, as described by [62], encompass factors that are specific to the employees themselves, distinguished from those related to the construction companies they represent for (e.g., general contractors). *Previous project experience* and the perceived quality of tender documents are newly identified factors that have significant influences on cost estimators’ judgements on cost contingencies.

### Previous project experiences

Previous project experiences was a critical factor influencing cost estimators’ judgements on cost contingencies. The participants noted that while both pleasant and unpleasant experiences can affect cost estimators’ judgements, they are more susceptible to the influence of the unpleasant ones (e.g., risk materialising, project failure). The severe substantial financial and reputational consequences associated with the negative outcomes in highway construction projects suffered by cost estimators can make them more inclined to overestimate the probability and impacts of similar risks in future projects, resulting in a pessimistic bias. This finding contradicts the views of [14] and [63], who suggested an optimistic bias among contractors, where they tend to overestimate the opportunities and underestimate the costs. The disparity in findings may stem from the specific emphasis of this research on highway construction projects, which are exposed to more severe consequences resulting from cost overruns compared to other construction projects (e.g., residential and industrial construction projects). Specifically, highway construction projects involve substantial investments, which can strain the economic growth potential in a region if cost overruns occur, necessitating huge additional expenses spent to complete the projects [4]. General contractors are hence recommended to accumulate adequate historical data so that cost estimators can adjust their subjective judgements accordingly to minimise the impact of either optimism or pessimism bias caused by their previous experiences [14]. Additionally, adopting an aggregation-based group decision-making approach is recommended. This involves collecting and aggregating cost estimates on cost contingencies from multiple cost estimators, which can mitigate biases and discrepancies that may arise from individuals [64].

### Perceived quality of tender documents

Perceived quality of tender documents from the client in terms of completeness and accuracy was identified as a critical influencing factor. The participants suggested that when the tender documents are perceived incomplete or incorrect, cost estimators are likely to allocate higher cost contingencies to tender prices to offset additional losses from uncertainties. For example, the cost estimators may need more resources or actions (e.g., project consultation) to acquire relevant information about the project, which will be included in the allowances. This finding is consistent with [6], who pointed out that cost contingencies tend to increase as the contradictions in tender documents increase. However, it is widely recognized that providing high-quality tender documents for highway construction projects poses a significant challenge for clients [4,65]. This is attributed to their unique characteristics, including wide geographical distribution [66] and dependence on economic, social, and political conditions [67], which increase the likelihood of scope changes during the project lifecycle [68]. Therefore, general contractors should promptly and effectively communicate with clients, raising their questions and concerns to improve the clarity and completeness of tender documents in the tendering process.

### Risk attitude

Risk attitude represents an expert’s disposition towards uncertainty, which can be generally classified as risk aversion, risk neutrality, and risk appetite [16]. Risk attitude has been widely recognized as a factor that has a significant impact on decision-makers’ risk ratings [16,22,28]. The findings suggest that risk-averse estimators tend to be pessimistic about risks, perceiving a higher probability of their occurrence and expecting more severe negative impacts, which results in higher cost contingencies allocated. Conversely, risk-appetite cost estimators tend to take a more optimistic stance, leading to lower amounts of allowances. [15] also found that under the same situation, risk-averse decision makers always assign higher risk ratings than risk-appetite ones. In order to mitigate the impact of risk attitudes, general contractors are recommended to encourage their cost estimators to attend relevant trainings and courses focused on managing personal risk attitudes. Moreover, hiring cost estimators with strong teamwork competency, rich work experience, professional education background, and high level of emotional intelligence is also recommended because cost estimators with such characteristics can better manage their own risk attitudes and are less subject to biases [69].

Cost estimators’ different risk attitudes can be attributed to various factors. Specifically, cost estimators who have experienced negative consequences as a result of their incorrect judgements on cost contingencies in previous projects, tend to be risk-averse. Furthermore, the level of completeness of tender documents provided by clients at the tender stage, particularly in the case of highway construction projects, can also explain the various risk attitudes of cost estimators [65]. As explained by [70], when cost estimators lack adequate and accurate information to make judgements about risks, they tend to be risk-averse. This is because without a clear understanding on project risks, they lack confidence in making well-informed trade-offs between risks and benefits. Moreover, this research identified that cost estimators’ risk attitudes can also be influenced by their previous project experiences. Cost estimators who have encountered unpleasant project experiences in previous highway construction projects tend to be risk-averse.

### Assumption of the controllability of risks

It was found that cost estimators tend to reduce cost contingencies when they perceive project risks as controllable, and vice versa. This aligns with [15], who found that experts’ assumptions of the controllability of risks can lead to optimism bias, making them prone to underestimate the probability of their occurrences and their potential negative consequences in the construction industry. Additionally, it was found that cost estimators with risk-appetite attitude tend to perceive risks as controllable while their risk-averse counterparts are inclined to view risks as less controllable. This is consistent with the findings of [16], who found that risk-appetite professionals tend to believe that risks are more controllable when projects are environmentally complex, while risk-averse professionals tend to believe that risks are more uncontrollable when projects are technically and organisationally complex. In comparison to other construction projects, cost estimators in highway construction projects are more influenced by the assumption of the controllability of risks. This is due to the presence of additional risks, such as traffic control and underground utilities, which add difficulties for cost estimators in assessing risks thus affect their ability to make accurate judgements on cost contingencies [71]. For instance, unforeseen underground utilities, such as gas pipelines, water mains, or telecommunication cables, pose enhanced challenges during highway construction, making it difficult for cost estimators to accurately assess the controllability of relevant risks [72]. This difficulty may arise from the lack of accurate record about the location and depth of these utilities. To mitigate the influence of cost estimators’ assumption of controllability of risks on their judgements, it is recommended to design a multi-stage process in which professionals are required to timely report their assumptions on the controllability of various risks to improve the transparency of their judgement-making processes [15].

## Conclusions and limitations

Determining proper cost contingencies plays an important role in finalizing general contractors’ tender prices, as it can not only reduce the possibility of cost overruns but also facilitate project success. Due to the indispensable role of cost estimators’ judgements in estimating cost contingencies while limited studies focusing on examining the subjective factors influencing their judgements in highway construction projects, this paper investigates the subjective factors influencing cost estimators’ judgement on cost contingencies. A total of thirteen influencing factors were identified through semi-structured interviews and a subsequent focus group study with twelve and seven experienced cost estimators, respectively. Among these factors, the ability to effectively communicate with stakeholders, self-reflection on previous practice, open-mindedness to project uniqueness, previous project experiences, risk attitude, assumption of the controllability of risks, and the perceived quality of tender documents were validated as the critical factors. Specifically, the ability to effectively communicate with stakeholders enables cost estimators to gather diverse perspectives from stakeholders, thereby enhancing the reliability of their judgements. Meanwhile, self-reflection and open-mindedness enable estimators to adapt and refine their estimates based on past experiences and unique characteristics of each project. Additionally, unpleasant experiences from previous projects and risk-averse attitude can lead estimators to overestimate risks, resulting in higher contingencies. Conversely, the assumption that risks are controllable can lead to lower contingency estimates if estimators believe they can manage those risks effectively. Furthermore, the perceived quality of tender documents plays a significant role in shaping judgments, with incomplete documents prompting higher contingencies to manage uncertainties. The research contributes to the current body of knowledge in two ways. First, it investigates factors influencing professionals’ risk judgements in the construction industry from the perspective of cost estimators. Second, it examines subjective factors influencing cost estimators’ judgements on cost contingencies in highway construction projects, a rather underexplored area in the current literature. The findings provide general contractors with a comprehensive understanding of the cost contingencies estimated by their cost estimators, thereby reducing the probability of cost overruns. Additionally, the results enable general contractors to enhance the reliability of cost estimators’ judgements on cost contingencies for highway construction projects by focusing on: enhancing communication among the team members; establishing effective self-reflection policies; recruiting cost estimators with rich experience and high emotional intelligence; providing trainings to help cost estimators better manage their own risk attitudes; improving the transparency of cost estimators’ judgement-making process; and implementing an aggregation-based group decision-making approach to get balanced views from different cost estimators.

There are a number of limitations in this research, which could be possible directions for future research. First, the influencing factors were studied separately under a qualitative research framework. Although some potential connections between influencing factors were identified, future research is necessary to investigate the relationships between the factors and their combined influences on cost contingencies by using quantitative research methods (e.g., questionnaire surveys). Second, for both interviews and the focus group study, although the participants were all experienced cost estimators working for different general contractors, the samples lacked variety in terms of gender as all participants were male. This might influence the research results because males and females are found to think differently in many aspects [73]. Future investigations are hence suggested to explore the potential differences in influencing factors for the judgements between male and female cost estimators. Third, since the purposive sampling technique was used in this research and only cost estimators working for UK general contractors were recruited, the research results can only represent the situation of the UK’s construction industry. Future research is recommended to investigate this issue in the construction industry of different countries, to obtain a more comprehensive understanding of cost estimators’ judgements in a wider context.

## Supporting information

S1 File Inclusivity in global research questionnaire. (DOCX)

S2 File Raw data set. (ZIP)

## References

[1] Infrastructure and Projects Authority (2016) National Infrastructure Delivery Plan 2016–2021. Available at: https://assets.pulishing.service.gov.uk/government/uploads/system/uploads/attachment_data/file/520086/2904569_nidp_deliveryplan.pdf. Accessed on March 2023.

[2] Highways National (2023) Planning for the future—A guide to working with National Highways on planning matters. Available at: https://nationalhighways.co.uk/media/2depj2hh/final-cre23_0370-nh-planning-guide-2023.pdf. Accessed on June 2024.

[3] National Audit Office (2022) Road enhancements: progress with the second road investment strategy (2020 to 2025). Available at: https://www.nao.org.uk/wp-content/uploads/2022/11/Report-Progress-with-the-second-road-investment-strategy-2020-to-2025.pdf. Accessed on April 2023.

[4] Abu El-Maaty A.E., El-Kholy A.M. and Akal A.Y. (2017) Modeling schedule overrun and cost escalation percentages of highway projects using fuzzy approach. *Engineering*, *Construction and Architectural Management*, 24 (5): 809–827.

[5] Love P.E.D., Sing C.-P., Carey B., et al. (2015) Estimating Construction Contingency: Accommodating the Potential for Cost Overruns in Road Construction Projects. *Journal of Infrastructure Systems*, 21 (2): 1–10.

[6] Enshassi A. and Ayyash A. (2014) Factors affecting cost contingency in the construction industry–Contractors’ perspective. *International Journal of Construction Management*, 14 (3): 191–208.

[7] Laryea S. and Hughes W. (2011) Risk and price in the bidding process of contractors. *Journal of Construction Engineering and Management*, 137 (4): 248–258.

[8] The Chartered Institute of Building (2018) New Codes of Estimating Practice. Hoboken: John Wiley & Sons, Ltd.

[9] Zayed T., Amer M. and Pan J. (2008) Assessing risk and uncertainty inherent in Chinese highway projects using AHP. *International Journal of Project Management*, 26 (4): 408–419.

[10] Ammar T., Abdel-Monem M. and El-Dash K. (2022) Risk factors causing cost overruns in road networks. *Ain Shams Engineering Journal*, 13 (5): 1–12.

[11] El-Sayegh S.M. and Mansour M.H. (2015) Risk Assessment and Allocation in Highway Construction Projects in the UAE. *Journal of Management in Engineering*, 31 (6): 1–11.

[12] Diab M.F., Varma A. and Panthi K. (2017) Modeling the Construction Risk Ratings to Estimate the Contingency in Highway Projects. *Journal of Construction Engineering and Management*, 143 (8): 04017041.

[13] Asamoah R. O., Offei-Nyako K. and Twumasi-Ampofo K. (2021) Relative importance of triggers influencing cost contingency determination for building contracts—the perspective of quantity surveyors. *International Journal of Construction Management*, 23 (5): 790–798.

[14] Hoseini E., van Veen P., Bosch-Rekveldt M., et al. (2020) Cost Performance and Cost Contingency during Project Execution: Comparing Client and Contractor Perspectives. *Journal of Management in Engineering*, 36 (4): 1–13.

[15] Dikmen I., Budayan C., Talat Birgonul M., et al. (2018) Effects of Risk Attitude and Controllability Assumption on Risk Ratings: Observational Study on International Construction Project Risk Assessment. *Journal of Management in Engineering*, 34 (6): 1–12.

[16] Qazi A., Daghfous A. and Khan M.S. (2021) Impact of Risk Attitude on Risk, Opportunity, and Performance Assessment of Construction Projects. *Project Management Journal*, 52 (2): 192–209.

[17] Laryea S. (2013) Nature of Tender Review Meetings. *Journal of Construction Engineering and Management*, 139 (8): 927–940.

[18] Brook M. (2017) *Estimating and Tendering for Construction Work*. 5th edn. Abingdon, Oxon; New York, NY: Routledge.

[19] Cantarelli C.C., van Wee B., Molin E.J.E., et al. (2012) Different Cost Performance: Different Determinants? The Case of Cost Overruns in Dutch Transport Infrastructure Projects. *Transport Policy*, 22: 88–95.

[20] Catalão F.P, Cruz C.O. and Sarmento J.M. (2019) Exogenous determinants of cost deviations and overruns in local infrastructure projects. *Construction Management and Economics*, 37 (12): 697–711.

[21] Kahneman D. and Klein G. (2009) Conditions for Intuitive Expertise: A Failure to Disagree. *American Psychologist*, 64 (6): 515–526. doi: 10.1037/a0016755 19739881

[22] Cathcart E.B. and Greenspan M. (2013) The role of practical wisdom in nurse manager practice: why experience matters. *Journal of Nursing Management*, 21 (7): 964–970. doi: 10.1111/jonm.12175 24028429

[23] Salas E., Rosen M.A. and DiazGranados D. (2010) Expertise-Based Intuition and Decision Making in Organizations. *Journal of Management*, 36 (4): 941–973.

[24] Kotzee B., Paton A. and Conroy M. (2016) Towards an Empirically Informed Account of Phronesis in Medicine. *Perspectives in Biology and Medicine*, 59 (3): 337–350. doi: 10.1353/pbm.2016.0029 28479576

[25] West A. and Buckby S. (2023) Professional judgement in accounting and Aristotelian practical wisdom. *Accounting*, Auditing & Accountability *Journal*, 36 (1): 120–145.

[26] Melé D. (2016) Understanding Humanistic Management. *Humanistic Management Journal*, 1 (1): 33–55.

[27] Beabout G.R. (2012) Management as a Domain-Relative Practice that Requires and Develops Practical Wisdom. Business Ethics Quarterly, 22 (2): 405–432.

[28] Ball D.J. and Watt J. (2013) Further Thoughts on the Utility of Risk Matrices. Risk Analysis, 33 (11): 2068–2078. doi: 10.1111/risa.12057 23656539

[29] Sellman D. (2012) “Reclaiming competence for professional phronesis.” In Kinsella E.A. and Pitman A. (eds.) *Phronesis as Professional Knowledge*: *Practical Wisdom in the Professions*. Rotterdam: Sense Publishers. pp. 115–130.

[30] Knudsen F. (2009) Paperwork at the service of safety? Workers’ reluctance against written procedures exemplified by the concept of ‘seamanship’. *Safety Science*, 47 (2): 295–303.

[31] Opoku A., Ahmed V. and Akotia J. (2016) “Choosing an appropriate research methodology and method.” In *Research Methodology in the Built Environment*: *A Selection of Case Studies*. pp. 32–49.

[32] Saunders M., Lewis P. and Thornhill A. (2019) *Research Methods for Business Students*. 8th edn. New York: Pearson Education Limited.

[33] West C., Kenway S., Hassall M., et al. (2019) Integrated Project Risk Management for Residential Recycled-Water Schemes in Australia. *Journal of Management in Engineering*, 35 (2): 1–11.

[34] Department for Business Innovation and Skills (2013) BIS Research paper No. 145, Supply Chain Analysis into the Construction Industry, A Report for the Construction Industrial Strategy. Available at: https://assets.publishing.service.gov.uk/media/5a7c08c040f0b645ba3c6499/bis-13-1168-supply-chain-analysis-into-the-construction-industry-report-for-the-construction-industrial-strategy.pdf. Accessed on October 2024.

[35] Acharya A.S., Prakash A., Saxena P., et al. (2013) Sampling: Why and How of It? *Indian Journal of Medical Specialities*, 4 (2): 329–333. doi: 10.7713/ijms.2013.0032

[36] Dworkin S.L. (2012) Sample Size Policy for Qualitative Studies Using In-Depth Interviews. *Archives of Sexual Behavior*, 41 (6): 1319–1320. doi: 10.1007/s10508-012-0016-6 22968493

[37] Hennink M., Kaiser B. N. (2022). Sample sizes for saturation in qualitative research: A systematic review of empirical tests. *Social Science & Medicine*. 292: 114523. doi: 10.1016/j.socscimed.2021.114523 34785096

[38] Davis K. (2017) An empirical investigation into different stakeholder groups perception of project success. *International Journal of Project Management*, 35 (4): 604–617.

[39] Seo W. and Kang Y. (2020) Performance Indicators for the Claim Management of General Contractors. *Journal of Management in Engineering*, 36 (6): 1–14.

[40] Schreier M. (2012) *Qualitative content analysis in practice*. Thousand Oaks, CA: SAGE Publications.

[41] Riggs R.J. and Hu S.J. (2013) Disassembly Liaison Graphs Inspired by Word Clouds. *Procedia CIRP*, 7: 521–526.

[42] Creswell J.W. and Creswell J.D. (2018) *Research design: Qualitative, quantitative and mixed methods approaches*. 5th edn. Los Angeles: SAGE Publications.

[43] Nyumba T.O., Wilson K., Derrick C.J., et al. (2018) The use of focus group discussion methodology: Insights from two decades of application in conservation Geneletti D. (ed.). *Methods in Ecology and Evolution*, 9 (1): 20–32.

[44] Howell B.K.E. (2019) “Methods of Data Collection.” In *An Introduction to the Philosophy of Methodology*. 1 Oliver’s Yard, 55 City Road, London EC1Y 1SP United Kingdom: SAGE Publications Ltd. pp. 193–210.

[45] Bhandari S. and Hallowell M.R. (2021) Identifying and Controlling Biases in Expert-Opinion Research: Guidelines for Variations of Delphi, Nominal Group Technique, and Focus Groups. *Journal of Management in Engineering*, 37 (3): 1–13.

[46] Nguyen, P.T., Babar, M.A. and Verner, J.M. (2006) “Critical factors in establishing and maintaining trust in software outsourcing relationships.” In Proceedings of the 28th international conference on Software engineering. New York, NY, USA, 28 May 2006. ACM. pp. 624–627.

[47] Saunders M., Lewis P. and Thornhill A. (2019) *Research Methods for Business Students*. 8th edn. New York: Pearson Education Limited.

[48] Howieson B. (2018) What is the ‘good’ forensic accountant? A virtue ethics perspective. *Pacific Accounting Review*, 30 (2): 155–167.

[49] Liyanage C., Elhag T., Ballal T., et al. (2009) Knowledge communication and translation—a knowledge transfer model. *Journal of Knowledge Management*, 13 (3): 118–131.

[50] South Gloucestershire Council (2022) Severnside Strategic Infrastructure- led Masterplan. Available at: https://consultations.southglos.gov.uk/SevernsideMasterplan22/consultationHome. Accessed on March 2023.

[51] Goh K.C. and Yang J. (2014) Importance of Sustainability-Related Cost Components in Highway Infrastructure: Perspective of Stakeholders in Australia. *Journal of Infrastructure Systems*, 20 (1): 1–9.

[52] Johari S. and Jha K.N. (2021) Exploring the Relationship between Construction Workers’ Communication Skills and Their Productivity. *Journal of Management in Engineering*, 37 (3): 1–13.

[53] Moberg D.J. (2007) Practical Wisdom And Business Ethics. *Business Ethics Quarterly*, 17 (3): 535–561.

[54] Cicmil S. (2006) Understand Project Management Practice Through Interpretative and Critical Research Perspectives. *Project Management Journal*, 37 (2): 27–37.

[55] Crawford L., Morris P., Thomas J., et al. (2006) Practitioner development: From trained technicians to reflective practitioners. *International Journal of Project Management*, 24 (8): 722–733.

[56] Taroun A. (2014) Towards a better modelling and assessment of construction risk: Insights from a literature review. *International Journal of Project Management*, 32 (1): 101–115.

[57] Jiang F., Ma L., Broyd T., et al. (2022) Underpass clearance checking in highway widening projects using digital twins. *Automation in Construction*, 141: 1–20.

[58] Kim H., Orr K., Shen Z., et al. (2014) Highway Alignment Construction Comparison Using Object-Oriented 3D Visualization Modeling. *Journal of Construction Engineering and Management*, 140 (10): 1–12.

[59] Kross E., Ong M. and Ayduk O. (2023) Self-Reflection at Work: Why It Matters and How to Harness Its Potential and Avoid Its Pitfalls. *Annual Review of Organizational Psychology and Organizational Behavior*, 10 (1): 441–464.

[60] Jenkins K., Kinsella E.A. and DeLuca S. (2019) Perspectives on phronesis in professional nursing practice. *Nursing Philosophy*, 20 (1): 1–8. doi: 10.1111/nup.12231 30450682

[61] Radzi A.R., Rahman R.A., Doh S.I., et al. (2022) Construction Readiness for Highway Projects: Key Decision Criteria. *Journal of Construction Engineering and Management*, 148 (2): 1–17.

[62] Ayodele O.A., Chang-Richards A. and González V. (2020) Factors Affecting Workforce Turnover in the Construction Sector: A Systematic Review. *Journal of Construction Engineering and Management*, 146 (2): 1–24.

[63] Flyvbjerg B. (2006) From Nobel Prize to Project Management: Getting Risks Right. *Project Management Journal*, 37 (3): 5–15.

[64] Monzer N., Fayek A.R., Lourenzutti R., et al. (2019) Aggregation-Based Framework for Construction Risk Assessment with Heterogeneous Groups of Experts. *Journal of Construction Engineering and Management*, 145 (3): 1–10.

[65] Creedy G.D., Skitmore M. and Wong J.K.W. (2010) Evaluation of Risk Factors Leading to Cost Overrun in Delivery of Highway Construction Projects. *Journal of Construction Engineering and Management*, 136 (5): 528–537.

[66] Ammar T., Abdel-Monem M. and El-Dash K. (2022) Risk factors causing cost overruns in road networks. *Ain Shams Engineering Journal*, 13 (5): 1–12.

[67] Ahmadi M., Behzadian K., Ardershir A., et al. (2017) Comprehensive Risk Management using Fuzzy FMEA and MCDA Techniques in Highway Construction Projects. *Journal of Civil Engineering and Management*, 23 (2): 300–310.

[68] Pai S., Patnaik B., Mittal A., et al. (2018) Identification of risks causing time and cost overrun in roads and highway projects in India. *International Journal of Civil Engineering and Technology*, 9 (3): 683–697.

[69] Taofeeq D.M., Adeleke A.Q. and Lee C.-K. (2019) Individual factors influencing contractors’ risk attitudes among Malaysian construction industries: the moderating role of government policy. International Journal of Construction Management, 22 (4): 612–631.

[70] Ward S.C., Chapman C.B. and Curtis B. (1991) On the allocation of risk in construction projects. *International Journal of Project Management*, 9 (3): 140–147.

[71] El-Sayegh S.M. and Mansour M.H. (2015) Risk Assessment and Allocation in Highway Construction Projects in the UAE. *Journal of Management in Engineering*, 31 (6): 1–11.

[72] Radzi A.R., Rahman R.A., Doh S.I., et al. (2022) Construction Readiness for Highway Projects: Key Decision Criteria. *Journal of Construction Engineering and Management*, 148 (2): 1–17.

[73] Arena C., Cirillo A., Mussolino D., et al. (2015) Women on board: evidence from a masculine industry. *Corporate Governance*, 15 (3): 339–356.

