# Supplementary material for: Critical factors influencing cost estimators’ judgements on cost contingencies in highway construction projects: An empirical study in the UK
Source: PLoS One. 2024 Dec 16;19(12):e0314665. doi: 10.1371/journal.pone.0314665 (PMC11649144; doi:10.1371/journal.pone.0314665)
Supplement: S2 File — (ZIP) [file pone.0314665.s002.zip › Focus group transcript.docx]

Focus Group Transcription

**Lilin starts with an introduction of this focus group discussion.**

**A warm-up task being conducted with the participants.**

**Lilin presents the participants with the mechanisms from the cognitive process model, derived from interview results, in a random order, explaining each one. She then asked the participants to collaborate in sorting these mechanisms to reflect their process for judging cost risk allowances.**

**Lilin: So, do you think the process shown on the screen aligns with your thought process for judging cost risk allowances?**

**P1:** Yeah, I mean from my perspective, it's all… it's a very two-way process. All of these things might happen in sequence, but then they happen backwards in sequence as well. I don't know whether anybody else has any thoughts on that?

**P6:** Yeah. A lot of it comes down to what's in your contract as well as to, I mean, we talking about ownership of risk.

**P1:** Absolutely.

**P6:** In my experience, the client doesn't want to take any risk and it all comes to the contractor.

**P1:** Yeah.

**P4:** Um, and if we had to rank those in order, I think the one (consideration of particular circumstances) that we've already got at the top probably needs to stay at the top.

**P1:** Yeah. I'd agree with that

**P4:** Because it's project specific, isn't it? And then probably the second one would be understanding the risk owners. Yeah.

**P3:** Yeah. I agree.

**P1:** Yeah.

**P2:** Understanding the risk owners. It means that, inside of the contractor side, so risk owners, is it estimating department or it could be commercial. It could be.

**P1:** It could be, yeah, it could be any of that, P2. It could be that, and it could be equally assigning it to whether it's a client risk or not. I mean, that that's tends to be one of the first things that, that we do. Isn't it? is identify what, whether it's client owned or whether it's contractor owned. If it's client owned, we largely dismiss it effectively.

**P3:** I think the next one (attention to risk elements) down is, is in the right sequence. That's when you're breaking down the detail of each of the risks

**P1:** mm-hmm mm-hmm (show agreement)

**P3:** um, and, and you can't have a perception of that risk until you know what the risk is anywhere.

**P1:** Yeah.

**P3:** So, you gonna follow a logical process. Now, this just draws us all out has been really bad for the ethical side. cuz it's...

**P6:** but we're estimators. And we are looking at it from the point… from a commercial point of view.

**P4:** So, if we were… if we were ethical, we wouldn't need contracts. Would we?

**P6:** Although, although, ... the ethics, may affect the business because if we, if we... I don't know if we, pump dirty water into a river, we can, well, we could still get prosecuted, which would be a commercial risk, but...

**P4:** Yeah, it's bad reputation as well. Isn’t it?

**P6:** It’s reputation. But as estimators, that's probably lower on our thought process than any cost.

**P1:** I agree. I think, I think I've probably received more challenge on virtue ethics from the likes of operations

**P4:** Yeah.

**P1:** when we get into, into discussing it with the... so we compile our risk register and then when we sit down with operations, they all said, "there go". Well, you cannot just be hiring six ton of silt down the water course without some consideration of what you're gonna do with it. which usually the response is, all right, what's the cheapest way of doing to move with it.

**P6:** So, perception of risk. Just, can you Lilin, can you just explain perception of risk to me again? What your...

**Lilin:** okay, , perception of risk is, you know, just a kind of feeling for the risk, um, this feeling you may, , based on the information you , about the risk and you can get the information from many different sources, like, um, your past experience or, you know, talking to your colleagues or talking to even people on site to get a feeling, just a kind of feel, you know, for the risk. It's something hard to express

**P1:** whether it's kind of high, medium, or low risk, that kind, that kind of thing.

**P3:** Suppose that that gets building... It's down your relationship with a client as well. Isn't it? I mean, you can have a risk that is a risk on one contract, but on another, you know, for a fact that the client is gonna operate differently or they're not gonna be as harsh on you, or they're gonna be harsher on you. So then that way, how, how much a risk weighs on your decisions can be affected by that.

**P7:** yeah. I think, I think as well, it's... It's interest that perception of risk is very subjective, cuz... That I find as we go through the review process, you, you, it's difficult to persuade your MD or your ops director that it's not a 2.5% job or a 5% job, because they've already got a perception of, of what level of risk... of the entire. And, and you, you might end up manipulated your numbers to come out with an answer that that's already the perception of where it should be.

**P3:** Yeah.

**P1:** Yeah. I mean, perception of risks, something that we, we come up with challenges against on, on every single learn. I expect it's... it's the same thing for everybody. No matter what your perception of the risk is, when you sit in a review of that risk register, it's... it's just largely putting it in the bin and start it again because somebody else has an entirely different perception of risk and then you do it again. And somebody else has a new perception of the risk.

**P4:** It... It evolves as you develop the estimate though, doesn't it. As more, more information comes in, as you get more program information, you get more certainty around your time related costs. You can in general reduce that risk amount.

**P6:** That's a less cynical view than the last one expressed

**P7:** I was trying not to be cynical.

**P6:** So, the discussion really is whether the attention to risk elements or perception of risks are in the right order. Because if, if we think that something is a high risk, we are more likely to look into what the cost implications may or may not be.

**P3:** So, on that, on that basis, do you sit them side by side one? The one doesn't it precedence over the other?

**P6:** Well, you could do.

**P2:** Yeah. Could do. I think. Because you can, you, you perception is maybe that the feel is gonna increase, but you don't know the, how much is gonna increase. You, you, you quantify this element, you know, it is an element of inflation, but you, it is a perception how much is gonna increase. So, yeah,

**P6:** Say. arguably inflation. Well, it is a risk because we don't know how much it is and, and everything is only a risk because we don't know whether it will occur or it won't occur.

**P1:** Yeah. Absolutely.

**P3:** I suppose from the, the, the detail of the risk. If, if you've got a site where the clients passing on you, ground conditions for unforeseen, obstructions or whatever, and, you know, for a fact that the site was previously used as a sports stadium or something that, you know, there's gonna be substantial foundations underneath there, that's never been removed. Then you need that detail before even a perception of that comes into account.

**P4:** Yeah, it is a lot of it comes down to the quality of information that's provided in the, in the contract documents. Isn't? Or the tender documents.

**P3:** Yeah.

**Lilin:** So do you all agree with this process? How, how do you think P5?

**P5:** Well, I, I think, P7 and he's... He's quite rightly said, you know, everyone perceives risk differently and, recently, I, I, I had a tender, discussing risk... the risk register and where we tried to the estimating. I tried to, you know, get it down as low as possible. So it's not to bump, bump the total price up and the commercial team have a different agenda and then they try to bump everything else. so, yeah, if ... there's certainly, very different perception between who is..., depending on who you talk to.

**Lilin:** Oh, okay. I see what you are talking about. It's like the factors where influencing your judgment, but, for, you know, for all your judgment, you may have, something in general, the thinking process. So, this is, this is what we are doing now, you know, try to find something, something in common in your thinking process.

Okay. So, sorry,

**P3:** just to add back to that was, I don't know who was doing the drawings of the arrows, ... that, that all then, like what P1 pointed out earlier, this all then feeds back up through the top again, and you would work, rework through the process because once you've determined what the risks are and you've seen what the detail is and what the commercial impact is, you would then read, read back through to understand it all again, and check back through your contract to see other angles, to make another amendment. Or if you, your decision is now being swayed now. That you, you understand the details of the job a lot more.

**P7:** Yeah. Yeah. So very much a circular process. Isn't it?

**P3:** yeah.

**Lilin:** Okay. So, for every step, go back to check the check the contract.

**P3:** yeah, it's probably less so for the, the, the very top one (consideration of particular circumstances), I think more, more often, that's a written thing that you've, you've, you've read and you've got. A hold on from the, the scope of works from the client, but it still can feed back into that. you could then need to just check to see, where your starting point was and whether you understood it right in the first place.

**Lilin:** Okay. Oh, I see. Also, so you mean the double error who weighs.

**P4:** It's definitely an iterative process. You'll go through it several times.

**P7:** Yeah.,

**P4:** and, and each time you'll try and put a, a value against that risk. Try and put something tangible against it so that you can either build it into your bid or, or exclude it or, or allow a percentage.

**P7:** Yeah.

**Lilin:** Okay. Okay. Thank you. So, are you now all happy with, with this process?

**P7:** Yeah.

**Lilin:** Okay. Okay. Okay. Thank you. I would say so. Yeah. Okay. So, in my plan, we will have, take a risk now. Oh, sorry. Take a risk now. So do you want to take a risk or you're happy to, you know, continue. I, I would continue. Yeah. I'm happy.

**P2:** Happy to, yeah. Yeah. Okay. I'm happy as well. Yeah. Okay. Thank you very much. Let me do something.

So yes, someone had come here. We will go to the third part.

Okay, and move it here, would you like to do Mount then move, right? Yes. I can see most of you are there now.

**Lilin:** We now have the thinking process you just work out together. But we know that this cognitive process, I mean the five blue boxes, they are not come out now here. They are facilitated by your knowledge. So, here we come to the three follow-up questions to investigate the knowledge you used in your thinking process. So just like what we did in the warmup task, would you please write down your answers and notes to your answers to these three questions? Because all the questions are about your knowledge, so you can write them on notes in same colour. Okay. So now you have two minutes to write down your answers.

Yeah.

I see some of you, some of you said previous experience, you make it more detailed.

For example, when you talk to someone, you can't just say my previous experience, maybe you can talk it in more detail.

Please write one answer on one note, please. Yes, please. Don't write all your answers on one note. Thank you.

And also, please write your answers to all the three questions, not only the first one, but for all three of the questions.

You can put your notes on the right on the left side at will, you know, left side of the blue books.

**P2:** That's. Oh, with the right on.

**Lilin**: Okay. We will have, one more minute to do this.

So we are answering the questions about, you know, what do you referring to when judging cost of risk requirements?

Because I see someone just answering the questions we will do in the next part.

**P2:** Okay. so, the, the time I have.

**Lilin:** okay, so, let's talk about, your answers. Would you please discuss in group about your answers and link every thought to each blue box, to show where the knowledge is used in this process, you can modify, your answers or, you know, delete something because they'll repeat. So let's start.

Maybe I think you can, move the note next to the blue box, because if we use arrows, it seems a little bit in a mess. Yeah. You please, in, when you moving the books, would you please discuss in group talk to each other? , explain, say something.

**P6:** Well, looking at the answers that are on there. The first thing everyone seems to be agreeing that we're going to look at historical data or the information that we've given or what we would call the works information, well, site information. So, we, so I think that is the... think everybody's pretty much said the same thing.

**P4:** Yep.

**P6:** I'm gonna look at the contract documents and the drawings and historical information that we've got... ground investigations.

**Lilin:** Oh yeah. Please move that to the box next to the box. I mean, that shows, you will use that knowledge in that, in that step. So, thank you, P6. Um, how about other notes?

**P3:** Anything, any reference to likely historical risks or historical working with clients and stuff will come to perception.

**P6:** Yeah. Well, this base… who's written and who put that on, but that's... that influences your perception of the risk. Doesn't it?

**P1:** Any, any feedback you're getting from your operational teams is the same, isn't it? Small feeding into your perceptions?

**P4:** en

**P3:** there's one there that's actually said, someone [unclear] for nonexperience of the client that could kind of fall between, the perception or the understanding of the risk owners. That kind of can go either way. There's more about one more.

**Lilin:** If any of you have different opinions or you agree with...

**P6:** all those, they influence your perception of the risk, but they also are contract specific. Aren't they?

**P7:** yeah.

**P3:** Yeah.

**P6:** How well the spec is written is contract specific... feedback from the commercial teams or all clerical risks on similar projects. Well… That that's... that's relative to a particular circumstance of each project. So, so they could go in, in both of those blue boxes, in my opinion.

**P1:** Yeah. That's, that's kind of where that contract document sits as well. Doesn't it? cuz the contract documents are both where you're picking up the particular circumstances of the project, but at the same time, the contract documents can tell you where the risk lies.

**P4:** en

**P2:** you copy these? Yes. just control C and control.

**Lilin:** Don't don't worry. If you want to use arrows, that's fine.

**P6:** Something none of us have actually got on there. I don't think is that... Passing of the risk? Well, that that's understanding the risk owner, but we could pass a lot of the risk. We've talked about the clients, but we try and pass a lot of the risk to the subcontractor or all the risk.

**P1:** Yeah. I got the point.

**Lilin:** So how about other notes?

**P6:** Experience of the client could fall into understanding of the risk owner because that if, if you know that the client has a lax interpretation of the contract, that would ... they will be prepared to pay for some of the risks, even if it's not necessarily in their court.

That thing I think that happens very often. These, those,

**P4:** I was gonna say, you're comparing lax with fair and reasonable.

**Lilin:** So how about the notes on the left side,

**P6:** Experience of the estimator and the overall bid team

**P1:** where people have numbered these, these are numbered in relation to which one we are talking about. So, one was the top one, two was the second. Three was the third. Is that what that is?

**P4:** Yeah,

**P7:** that's what I, that's what I did.

**P6:** Yeah. Yeah.

**P1:** Let's go on.

**Lilin:** So how about the last box? I see no one put any knowledge against it.

**P4:** I don't like that. Ignore. Oh dear. I don't think my bid manager would like.

**P3:** To be fair. I think that one is more of a scheme specific sort of thing. It's difficult to judge without actually having any detail.

**P4:** It is. Yeah. Yeah.

**P3:** It would be considered in line with the other, whatever the risks above it is. And so once we, once you assess the actual risks, then you look at the ethical side and the environmental approach to things and see if it's, um,

**P4:** say ethics also comes out of your, your relationship with your client. Doesn't it? So, experience of the client could come under ethics. If you've got a clarent client that you know is gonna be fair and reasonable with you, you probably have a different approach.

**P3:** Yeah.

**P4:** Your risk profile, then you would with somebody that was very hardnosed.

**P3:** Yeah. If you know somebody who's gonna be very contractual and that's not the way that you operate or vice versa.

**P4:** Yeah.

**P3:** then there's a bit of a disconnect and. You, you could have issues that, that you need to cover off as a risk.

**P4:** en

**Lilin:** For someone who write technical knowledge of the risk factor. Can, can you explain it further?

**P6:** That's really attention to risk element sort of thought. The technical knowledge.

**P5:** Yeah. I, I was being a bit, generic there with... in answering question two. so I mean, depending on the project and the client., you know, if, if you're working on city center location... there such a thing as where CATV, underground CATV might be predominant. And therefore, when, when you start digging, you allow certain risk into your scheme budget or, or target price. Just so in case, that this you uncover CATV. Yeah. So, a little bit of local knowledge of technical.

**P6:** Oh. That comes into consideration of particular circumstances. And doesn't it? Yeah, the risk owner, but the risk owner is the contractor. If he damages services. So you, your perception of risk is based on your technical knowledge.

**P5:** Correct, but like you said, perception. So, you know, we, we talked about it and some someone else, thought it to mean something else and, looked it under understanding of risk owners. So, so it's not, it's not entirely wrong either.

**P6:** No, there are no right or wrong answers.

**Lilin:** Well, do you have more things to share about the knowledge you used in this, thinking process? So, um, are you happy with the notes or the position of the notes? I see. I see, I see P7 just write a new note against the last box. Would you mind to give some explanation?

**P7:** I saw it was a bit empty that last one. So, I'm just trying... it's a sort of... we, we, as a business, we, we will, we sort have a perception that, that we're good with with the ethical side. And we, we, we generally are. But I think as far as the overall scheme, we have an assumption that if there are issues with protesters, with planning, with all of that sort of thing. That, that the clients looked after that. And that it isn't really our risk as to whether for just taking it to the extreme, whether the job should be built at all.

You know. If it's in the right place, wrong place with sort of... I do not explain myself very well.

**Lilin:** No worries. Um, so are you all happy with the notes on the Blackboard? yes. On the whiteboard. Okay. So yeah.

**P4:** Yeah. I think fair...

**Lilin:** Thank you for sharing this. So, so far, our discussion is based on assumption that judging cause risk allowance is a rational behavior. And also, we haven't talked about the context when you judging the cause of risk allowance. So, but you notice that, an estimated judgment can change from one project to another and also for the same risk, different estimators, they can have different judgment. So, zoom out then move right to our last section. You can see four questions on the screen. Oh, can you all see that? The question is one judging the same risk, what causes estimators to have different judgment about this? Cause response. If I can see all we here. so, like what we just did, you have three to five minutes to write down your answers. You can, if someone share the same opinion with you, you can just like it notes. If you can't see the like button just looming, make it bigger, then you can see, see that button.

Okay. Now we start.

If you want, you can change the color to distinguish your answers. Okay. we can. we can, I think we can discuss the answers and write answers in the same time at the same time. So, for all the notes on the screen, we can see it, you know, as the factors that will make estimators judgment, um, biased and subjective. So, would you please discuss in group about your answers and also at the same time, classify them into different categories and also name the category?

**P1:** Do they classify them into different high, medium, or low kind of thing?

**Lilin:** Maybe not based on that one, based on something more descriptive. You can discuss about this like. How you will classify the notes, what do you see, the factors in common, um, what are the similarities? What is the difference?

**P6:** Should we take question by question then of something and, and?

**Lilin:** Actually, all of the notes here, we can see them as the factors, which will make an estimated judgment, biased and irrational. So, some of the factors may, you know, controllable, but something is out of the control of this matter. So, I'd like you to discuss in group about firstly, about your answers, why you. This is the factor and then make them into different categories and also name the category based on your discussion. Just no right or wrong. Just your opinion.

**P1:** Yeah, I mean, for, from my perspective, I think one of the things that affects judgment of cost risk allowances is, and, and appears to be fairly large consensus, there is being experience.

**P4:** en,

**P1:** whether that's experience on specific projects or experience with clients.

**P4:** Unfortunately, it's usually a bad experience as well. Isn't it?

**P1:** Yeah. 90, 99%. Yeah. So, I think, I mean, if we were gonna put something in a category, I would suggest that experience is probably one of the categories.

**P3:** would you have experience and knowledge in the same group or would you split them off? You can have a working knowledge of... sorry, knowledge of something that you maybe haven't experienced, but

**P4:** yeah.

**P1:** So, do you want us to move these around into categories then? Little result?

**Lilin:** Um, yes. Yes. Put notes which are in the same category together. I think you can firstly, do this and, and then talk yeah. Then discuss about them

**P6:** nearly all experience they are.

**P7:** Yeah.

**P1:** Yeah.

**P4:** Everybody said the same thing.

**P1:** Yeah. I suppose the only thing that's, that's slightly different are that appetite to secure the contract. Slightly different from experience and knowledge in that, in that first section. Yeah. Cuz everything else I think everything else has been knowledge and experience.

**P4:** It's a valid point though. Yeah. You know, if you're keen to win a job, you'll have a different outlook on it then otherwise

**P3:** yeah. Determines whether you think it's gonna win or not, then, then how much you wanna win the job.

**P6:** there's a school of thought that says that estimators should, both. What they think will actually happen. And it took for someone who's probably paid more than I am, to make those type of decisions to how keen we are to win the job.

**P4:** Yeah. Look, I don't about you guys, but my philosophy has always been not to win work as an estimator. My job is to advise the owners of the business, what it's gonna cost to deliver an element of work. And that's it.

**P6:** That's that's what I, that's what I have always seen our role to be.

**P4:** Yeah. Yeah. Anybody can go out there and win a job.

**P6:** Yeah.

**P7:** Yeah.

**P6:** And when you have your review, you point out whether there's opportunity

**P4:** Yep.

**P6:** Or you think the risk is high. So you are expressing an opinion, but the opinion that you give at the time you price something is your genuine assessment of what you think the job will cost.

**P7:** Yeah. I think what, what, what I've always said is, is this... these two things involved in estimate. One's putting the estimate together and the other one is the tender.

**P4:** Yes,

**P1:** absolutely.

**P7:** From the estimate. And what, what people want to do with the estimate is, is their business. But the project is, is sacrosanct. Isn't it?

**P1:** I'm just throwing some additional post at top there, cuz I'm just trying to find some common ground.

**P5:** No, that's fine.

**P3:** Is that desire to secure the work, the actual note that would go under it in the category is business objectives?

**P1:** Yeah, that's probably a more, appropriate a title for it,

**P3:** but the desire to secure the work would be one of the...

**P1:** I think that's the appetite to secure the work is fine for that.

**P3:** Yeah. Three categories are enough to cover everything we've got there.

**P1:** So, one of the things in relation to that third question, what aspects of the company that estimator has influenced them in judging cost risk allowances, is one thing that I have found, is when an estimator has gone into a review for a risk register for a project. If they have been particularly burned or have... it has come out extreme... like sufficiently more optimistic than they thought or something like that. That tends to influence how they approach the risk register the next time..., I don't know whether anybody else has that similar experience?

**P4:** Yes. I agree. Unfortunately, sometimes that comes as a result of bad management on the project delivery side as well. But it's very, very difficult to, you know, at the end of the day, it's... there perhaps things that, or events that perhaps shouldn't have happened, but all the same, they are still a risk and still a cost to the business.

**Lilin:** You can all move the notes and name the category and, you know, just tell people why you thinking this way.

**P7:** I think we're saying that the four posters... notes at the top of the headings that we're looking at, aren't we?

**P1:** Yeah,

**P7:** that was the way I was... I've been looking at it anyway, but yeah,

**P1:** that's what, that's what I was going for. Yeah,

**P7:** yeah, yeah.

**P1:** Loads of the much experience, isn't it?

**P7:** Yeah.

**Lilin:** So, in this, process, for each note, would you mind to give some further explanation? I will say each, maybe you can pick one, you think are most important or you think have the greatest influence on an estimated judgment as an example.

**P1:** Yeah, I think, I think what I, what I just mentioned before in relation to risk registers, I think previous, recent previous experience of estimators can, can heavily influence how they're going to review risk registers and how they approach risk registers. So, if they've been in a, a risk review on, on one tender and taP4 a bit of a battering, the next risk review they go into, they can be a bit more, either conservative or optimistic, depending on what the reason for, the mismanagement of that, of that previous review is. Tend to find that... that is quite a, quite a big thing for us.

and yeah, I suppose the, the business objectives in terms of the appetite to secure the contract, that's always a massive one for us.

**P7:** Yeah

**P1:** no matter what the estimator's view of the, the risk is, they can go into it with one view of the, the risk and opportunities on the tender, and they can largely be thrown out or not thrown out, but, but heavily altered because of the business objectives. And that, that foresight for estimators, if they know that the business isn't particularly interested in them. As a result, then the attention they appear to those risks tends to weigh a little bit more.

**P6:** So, we are sycophantic, then we, we tell the directors what they want to hear

**P1:** often. Yeah. Yeah. I, I think there is a lot of confirmation bias goes on.

**P7:** Yeah. Yeah.

**P4:** Huge amount.

**Lilin:** So P4, how do you think, can you pick one, one of note, you write. To have explanation.

**P1:** I ...I've gone. So, somebody else can go this time.

P2. You're very quiet, but there...

**Lilin:** so how about you, P5? Can you pick one, pick one note to write and explain, you know, share your, your opinions with others?

**P5:** Yeah, well... on the question of, what makes estimators irrational, I think, as I said, humans are generally irrational, so it's not as estimators, same as everyone.

**Lilin:** So, how about you, P3? Can you pick, one or to write at well, to have a further explanation, um,

**P3:** to be fair that… I put on there about knee jerk reaction, previous experiences, similar in what P1 is saying, but from more of an operational side that sometimes the, ... there can be something going on on a, on another scheme on site, that can then directly influence the, the current project that you're pricing in. That something has gone wrong or something's gone, right? Whichever way you wanna look at it and for whatever reason, you've then gonna change your, ... your output of how you're gonna approach your risk for each of the, the, the, the things on your project, because it's happened on another one. And I don't necessarily see that there, there actually is a, a link between them. Cuz every scheme should be on its own merits but it has in the past... got to a point where you're discussing these things with directors and they know what's going on on a previous site and they can't get pass that.

**P4:** Yeah.

**P3:** So, so that they they're hooked up on it on... we've got this issue. We need not have this issue on this job and you, you can't quite tell them it's not gonna happen on this one, cuz there is that risk still there that, it could, but... and they, they're still gonna the fact that it has happened and it is happening. So now it has to happen on every job.

**P7:** Yeah. Yeah. I hundred percent agree with that. It's... it goes back to the risks that are on the risk register. You can generally manage those and there shouldn't really be an excuse for them not being managed. It's when stuff comes out a left field. That is, it anything that would end up on a risk register until that actually happens, and then it's the first thing on top of the risk register for the next tender.

**P3:** Yeah. And at times there is that that, that could come up, that that should happen. Like when COVID first struck things like that... that that's fine. That... that continues through. It just, it doesn't necessarily have to be everything that happens becomes the, the most important thing every time.

**P7:** Absolutely. Yeah. I mean, what if it happens once, it... it's from left field, it shouldn't really be happening again. Should it?

**P3:** No.

**Lilin:** Okay. Do you have more to share or do you all have be with the categories and notes on the screen?

**P4:** No. I ran out of time really on that last one there on the bottom right, about what aspects of a project can influence estimators. There's lots of, lots of aspects that can can influence. And every, every job is gonna be slightly different, but our biggest cost are our time related costs. So, the program has gotta be right. Programmer works is, is essential having an, an accurate program that perhaps has gotta a little bit of fact in it for some wet weather and some rework and, and the like, but, that program dictates all our time related costs. So, our supervision, our site facilities, our traffic management, a lot of our temporary works. They are huge costs. And, the program is, is essential.

**Lilin:** Okay, thank you. Thank you, P4. Oh, are you all happy with that? Anything else to share? No, it's good for me. Okay. That, yeah, let me save the screen. Thank you for sharing this. Um, I will now show you the model I produced and please feel free to share, to make any comments. So, we are a little bit wrong out of time, can you see the screen? Yeah. Yeah. Um, so this is model I produced. You can see the five blue books. The sequence is a little bit different from what you just made. And you can see there is dotted lies and solid lies. The dotted means, you know, the connection is weak and the solid means it's a strong connection. And based on my interview results, I, I think the big difference is where it's the step of consideration of particular circumstances of each project. Um, based on my interview results, the interviews they were firstly said they were, the first thing they will do is to look at the contract conditions, to see who holds the risk, because they do not want to waste time to, to price risks, which are held by other parties.

And I think for, for most, yeah, for most of sequence, we get the same. And then for, you can see on the orange boxes, the technique and SIS, in theory, this is two kinds of knowledge technique. Um, you can understand as skills you used and PHIS is the kind of knowledge you acquired from your experience. I think you just mentioned several you acquired from your experience and this is, there is also strong, solid life and dotted life to show that their, strong connections and weak connections. And for the other three robotics, individual level sources of bias organizing, yeah, the three boxes, this is what we talked about. The last part we talk about, and this is the categories I made for the individual level sources or sources of bias. I mean, that something. Um, estimators can control. Um, but for the organizational level sources of bias, there are something where compare the workload and the size of the business.

This influence cannot be controlled by estimator then for the other one sources of errors, I distinguish arrow from bias because bias doesn't mean error because, some bias, can help, can even benefit people in doing the judgment. So, for this, model, um, do you have any comments or do you have any, you think any areas need some modification?

Can, do you think this model can represent how you judge the cost of risk comments?

**P1:** I think the only thing for me is, is what we mentioned earlier on about all of these things, it's, it's not a, it's not a straight line

**P4:** en

**P1:** the process happens and it happens again and again, and again again, until you get to the point where you're, you you're happy with where you, where your risk profile sits. That, that would be my, my main comment on this. ... this the model, I don't disagree with the model. I just think that there's there's it is an iterative process. It does go back and we do, we do review the, the individual aspects again.

**Lilin:** Okay. Thank you.

**P6:** So, I think we got those in slightly different order, didn't we? Because I think we all agreed between ourselves that you have to identify the risk before you can understand who the risk owner is. So, you can't have understanding of the risk owner at the top because it comes after you've identified the risk.

**Lilin:** Okay. Thank you. Thank you. Do you have any other comments?

**P4:** Not for me. I don't think.

**P7:** I learned a new word today. techne and phronesis.

**P4:** Yeah, me too.

**P7:** Confused that I, I was struggling until Lilin told me what it meant.

**Lilin:** um, actually there are three forms of knowledge in theory, the other one is the theoretical knowledge you learned, but I didn't show this in the model because, you know, in the interviews, no one mentioned, they will use some theoretical knowledge in their judgment process. So, did, so how do you think about this?

**P1:** What do you mean by theoretical knowledge?

**Lilin:** Um, like, like something you learned maybe from university or from, um, or, or we can say the rules set by the company or set by the department to, you know, teach you how to do estimating something like this. Think there, anything like that, that you've learned, um, by the time you've done something, once it then feeds back in as experience, rather than just the knowledge.

**P3:** So, you've learned it to do it, but then you start, cuz you try to do something then it's, it's not... You're not really classed it as knowledge anymore, to more your experience.

**P7:** Yeah. Yeah. I agree with that. Yeah.

**Lilin:** How about you, P5?

**P5:** I'm on mute. Yeah, I sort of agree. And, I mean theoretically, I don't think I've... I'm not, I'm not aware that I've use any theoretical knowledge. But certainly, I do use a lot of technical knowledge, um, because we have to price the work to a certain technical standard. Set by say, department of transport.

**Lilin:** Okay. Thank you. Um, do you have any other comments or, yeah, I think both, what you just mentioned, most of the problems is with the, this is not a one-way process, but integrity. Oh, no intuitive process. Yeah. Yeah. So do you have been with the categories of the sources of, I would say the source bias and arrows, I mean the way to classify the factors.

**P1:** Yeah. I think the sources of errors, definitely.

**P4:** Yeah.

**P1:** It's pretty, pretty bang on. Yeah. Yeah. Same, same for organizational level sources of bias. I would, I don't know what others would say... I would say that that was a stronger connection than, than, than weak, from my perspective, the organizational level one. Cuz they tend to sit, when we, when the organizational level, ... I don't know whether I, I would necessarily call that part of the cognitive process of whether that's just part of the process that tends to come in when we, when we get to review stage, certainly, certainly here it comes in at review stage where, the big boys come in and decide that their bias based on the, on the business, the workload of the business, et cetera, et cetera, might be more.

**Lilin:** Thank you. Do you all happy with the boxes named sources or errors because, you can see, I give some examples, like completeness and accuracy of information, about risk and the availability of amount of historical data about risk. Because I think these factors are something, um, definitely will lead to arrows in an estimated judgment.

**P4:** I mean, the information you've got that you're basing your, ... your decisions on are incorrect, then, it's certainly gonna have an influence on it in, in a negative way.

**P1:** Yeah. I think the source of error box is good. I think that's really good.

**P6:** Yeah, it is. For me, the, the thing about risk is, it's risk because we don't know whether it will occur or it won't occur. So, it, it's difficult to say that there is a source of error when you're actually pricing something that you don't know what's gonna happen, because it's all about probabilities.

**P4:** en, I'm talking about historical data as well. If, if something's happened before. Next time round you, you sort of try and manage it so that it doesn't happen again. So, it's no longer part of the risk. It's part of the, the estimate, isn't it?

**P6:** Yeah. If you know it's gonna happen, then it isn't a risk. Yeah. If you know it isn't going to happen, it isn't a risk. So somewhere in between lies and the truth, which we, we, we, we, and, and when you price a risk register, some things will occur and some things won't, and hopefully you'll end up with enough money in the part to cover what does happen on site.

**Lilin:** So, so this is actually, you know, a kind of skill, I would say the technique, because, you know, you are balancing between whether you rely on a historical data or maybe your past experience. It's like a balance of combination.

**P6:** An example somebody made earlier on was, if you, if you are working on a site, that's previously had some kind of structure on it and you know that it's only been demolished underground level. So, you know, you're gonna find some hidden foundations or whatever, but if you know exactly where they are, then it ceases to be a risk because you can actually quantify where they are.

**P4:** en.

**P6:** But if you don't know where they are, then you're taking a punt on the fact that your new foundations may clash with some existing foundations, but you don't actually know where. But you know that somewhere they quite likely will. So, what percentage of these new foundations will clash with the old foundations? 5%, 10%, and 100%? And if you are taking the risk for that hard excavation, if you don't know the answer, then it's a risk. If you do know the answer, isn't a risk. So, so completeness and accuracy of information. If, if the information was complete and it wouldn't be a risk with it.

**Lilin:** Yes. Hard to say

**P5:** it's, it's the known unknown. Isn't it [name]?

**P6:** Yeah. It's the known unknown. Yeah.

**P5:** Yeah. And then there's the unknown unknown.
