# Supplementary material for: Critical factors influencing cost estimators’ judgements on cost contingencies in highway construction projects: An empirical study in the UK
Source: PLoS One. 2024 Dec 16;19(12):e0314665. doi: 10.1371/journal.pone.0314665 (PMC11649144; doi:10.1371/journal.pone.0314665)
Supplement: S2 File — (ZIP) [file pone.0314665.s002.zip › Transcription (interview A).docx]

**Interview A-Meeting Recording**

**Interviewer:** OK Thank you. So, would you mind to firstly, have a brief introduction of yourself, for example what work do you do in your company?

**Interviewee:**  Yeah. So, I'm a managing estimator. So, I worked for a contractor that turns over around 30 million a year. We work as a civil contractor, but principally we do a lot of highway schemes and probably road schemes. So, stuff for local authorities typically. But we do work split. Sometimes as a sub-contractor as well, as well as a main contractor. It's probably a 50 50 split. But if we work as a subcontractor and the main contractor is doing a building and there's associated highway work, so we would deliver the highway works for them. So, our average order value probably is around about 2 million. But we probably, the range is something like from 200,000 to up to about 10… 12 million would be done. So just schemes we typically do so.

**Interviewer:** So how do you become an estimator? Do you plan to do this?

**Interviewee:** No. Probably not. My background then was I did a civil engineering degree a long time… well, not that long ago. I graduated in 98s, so and then I worked for a contractor that I'd work for on work placement. So then sort of a fairly traditional route as a site engineer, so the agent, site agent, project manager, did a design so common of my, sort of chart, become chartered. I guess… I thought I would probably be more likely to be a contracts manager, area manager, regional manager of that. But one of the contractors I was working for at the time had a framework with one of the local authorities where we did some of my 15 million a year. And they had an estimator on that who left the business. And I'd got approached from … internally to see if I'd be interested in doing some estimating.

I was still on site at the time. I'm got probably… initially I didn't think I did want to do it. And then I agreed to do it almost as a 12 month. It's common because I thought it'd be good commercial experience to acquire. For a career, it probably in a different direction, but I liked it. And then I stayed sort of doing pre-construction stuff. So, then the estimator, senior estimator and now managing estimator, all be of a relatively small team. We got about seven people in total. So, I manage the department. So, you know, but we have quite a flat structure. So, although we've got seven people in there because of the size of business we are, I still have to price works myself, probably just less than some of the others do, but yeah.

**Interviewer:** Yes. So, in the projects you involved, can you pick one risk, which happens most, and talk about all the things you think about while making judgment on it?

**Interviewee:** Okay. Probably the biggest risk that we encounter in highway works would be utilities. So, stats, so any stats that are in the ground. Both ones that, you know, need diverting or protecting or uncharted ones. And then it's not just... The cost of actually doing those works. It's the performance of the utility providers themselves, cuz it can have such a huge impact on the program. So that's probably the biggest one. And I would suggest what we normally do with that. We try and push that risk back onto the client is the thing that we really try to do.

And it would be in terms of some elements of the stats. We wouldn't accept the risk at all. So, it would be a showstopper for us because there'd be some… if there's not… for instance, if there is no information on the stats at all, clearly you just couldn't take the risk because you could have a worst-case scenario. There might be a BT fiber optic cable. That's a million quid to divert. But you're pricing a 200,000-pound scheme. So, it's just the magnitude that potential risk would mean that you couldn't even look at pricing that risk.

If they've done enough detail work, we would consider taking on that risk and then how we'd then probably assess what we did with that or how we viewed it would be if it was on the critical path of the program. Cuz obviously if there's some floats in the program for when these diverse works needed to be done, we'd be a lot more comfortable than if it was on the critical path. Then it was going to extend duration of the whole scheme. The level, I guess, the level of what those stats diversions that I get, if we're having to take the cost on board of them, we'd probably be more amenable if it was something in the order of tens of thousands, rather than hundreds of thousands.

And again, we may well often because of the timing with stats, they get placed by the client in any case, because they need such a lead time that they've often got a 16-week lead time. So, by the time the contractors in contract, the client would like to place those orders. Otherwise, they won't start on site early enough.

But yeah, so we'd really typically try and push that risk back to the client. But if that was floating the program and it was fairly low value, we would price that. That probably, that might not be a great example for where we taken risks, cuz we'd be very reluctant on taking the risk on stats, but it probably is that the risks that we're, that we come encounter the most. I don't know if, what if I took a different risk for the purposes of this to understand how we assess it might be better for that question. I don't know.

So, one of the other ones we'd have, would be ground conditions, I guess. So, and again, with all risk, it probably, it depends on how much information there is. So, if there was no ground investigation carried out, I guess we'd probably make an assumption and try and qualify that and put that risk back on the client. If they've produced, then depending on the level of ground investigation that's been undertaken. And I guess then that probably so it goes hand in hand with how comfortable we would be with taking the risk.

And I guess also what the potential volume of earthworks you're looking at. But so typically with the physical conditions, the ground conditions, it might be that your construction debt is going to increase. And obviously if they give you fairly comprehensive boreholes with CBR, so bearing ratios along the route of the highway, you could probably make quite an informed view on that. And then you might go, well, we might, we're really just, the average CBR’s 4%, we will base the design on a 4% CBR, but we are likely to encounter scenarios where we might have to seek in the construction. And if there's quite a safe, quite comprehensive boreholes, you'd probably be able to plot that on and then go, well, I think it's probably going to be sort of 10%. And so, 10% would have an extra… whatever depth of capping and you could put some number to that and disposal.

So, it is really, probably based on what information, the clients that got ready for you at tender stage for how we assess that. Again, we… ideally… we would probably still like to qualify if we could. Cuz, I think we'd still put, try and shift it as much risk back to the client as possible. I guess the other thing that would change it would also be what the conditions of contracts are. So typically, we're working on doing NEC contracts, I guess, and, as long as they've not massively changed those through Z clauses. We'd be very… generally very comfortable with the base risk that you're taking under those conditions of contract. What happens is you normally get developers that then put some Z clauses in that. They remove some of the core clauses and you'll recourse to have a compensation event, I guess. So, it's all a bit. that's all sounds a bit wordy, but if we've got to take the risk, I mean, it becomes really probably, I guess, of assessing what we think the value of the risks would be and then a probability and then generating some kind of number for that. And then, you'd probably have a number of obvious risk on you. We'd have a risk register for every tender, risk and opportunity.

And we did the same with opportunities as well, cuz there will be potentially, I guess, in that example, I've just said about the ground conditions. You might have some areas where you're gonna put less construction in there. So then in the round, once you've got your risk register with your probability and cost and your opportunities the same, then you might have a pot of money then that you would then either add, or... I typically add. I don't think... the risks normally are higher than the opportunities. I'd suggest on most of the work that we do. So, you might then have some science behind a pot of money that you'd allow for risk.

**Interviewer:** So, from what you said it seems that the attitude to risk of your company is more risk averse?

**Interviewee:** Yeah. Well, I completely agree. Yeah. We do very ... we did limited schemes that on a design and construction basis. So, I guess that's where we're running. In fact, to be honest, while I was a few minutes late, we were discussing, we've got a design and construct tender in at the moment. And the design is very preliminary. So, we were talking about, I think the client would like a all-risk price on that highway scheme. We're not sure that we could do that on the level of information they've got. So, we're sort of going to approach them to see what their view would be having a highly qualified bid.

**Interviewer:** So, what is your personal attitude to risk? Do you think its the same at your company's? And do you think this will affect your judgment on risks?

**Interviewee:** I'm probably fairly conservative with the risk, I think. Yeah. Fairly risk averse it. So, I think having work for bigger… almost if you're a bigger organization, you're spreading your risk over more contracts as well, aren't you? I guest. So, you can almost have the view that all that's civil engineering contracting… you do well on one, but you lose on another one and in the round, it works.

I guess cuz of the size of business we are, if you took a big head on one contract, it would have created more of a ripple for the business. So, and we do do a lot of schemes where the scope is fairly fully defined. So, the risks were then looking at, I guess… inflation, which I guess you can take a better view on. Although at the moment, the market's a bit volatile on that, we've probably gone through a long period of fairly stable.

So, price is… because HS2 is now sort of started in earnest, and obviously coming out of the pandemic and there's a lot of demand again now. So actually, inflation's is more of an issue now. We're seeing our supply chain only hold their prices for maybe two weeks. And when you know, you're not gonna be placing an order for until six months time about. It's difficult to take a view on. But that's probably been very recent and say almost in the entire time I've been estimating, prices have been very stable. So, we might then be go, well, historically that, you know, the indices would be .... is going up one and a half, 2% a year. So, we could take that view. That's probably become a little bit more challenging at the moment. So… but yes, but your question was, are we, yes, we're quite risk averse, and I'm probably quite risk averse is probably true.

**Interviewer:** Okay. So, what do you think your attitude to risk affect your judgment? How will it affect?

**Interviewee:** I see sort of estimating… as to me, you get your base price as you see it, and then you identify the risks and opportunities, and then you go to a tender adjudication. And probably on a bigger scheme, you might've had a mid-tender review and you present that information to... for me here, it would be... I'm managing director. Discuss what you think the risks are, what the values the risks are and then the commercial judgment is often made by collectively all those above. But I'll see informed on the information you've told them. So, I guess it almost then becomes… in this instance, we're a private company. It's actually who I presented to is it's essentially his money on the line.

So, it becomes almost his. So, for me, it's displaying what I see the risks are. And then you gauge how it's... probably his attitude to risk that probably then ultimately informs what we then do with that tender. And that then might vary also depending on workload as well. If we've got a very good order book, we're probably likely to be more risk averse, I've suggested. Then if we have a gap in the forward order book and we need to find homes for staff and our gangs and labor. So, then we're probably likely to take more risk on board because we'd probably in a situation where we have to be of sick spending money if we've not secured work anyway.

**Interviewer:** Yes. So, for this judgment process, do you think it's more objective or subjective because for example do you think estimators will make different judgment on the same risk under the same condition?

**Interviewee:** Yes. Yes, absolutely. I think there's a part... there'll be some science ... some analytical science behind what they do, but it will be informed on to an element on their view. And you're right as their attitude to risk. So, we strangely, I guess… of the three estimators we've got, I could always pitch in whole where what all of our attitude to risk is.

I'd probably suggest that we've got one who's definitely more cavalier or would be more accepting of risk. He's a glass half full man and he doesn't see the same risk that perhaps the other two do. So, yes, I think you would get, and I think then the key, so saying that our tender judication process. I think that whoever's actually ultimately adjudicating a tender, almost needs to understand what that estimator's view on risk normally is as well, because there'll be people that are probably incredibly risk averse. We had a previous estimator who was very very risk averse, and you'd then probably be taking money out of a bit with his. I'd suggest that it would be a very robust price, but you might not win the work. And then at the other end of the scale, we have another estimator who you'd be likely to putting it a little bit on generally, because he's, as you say, his view on risk would be the other way.

**Interviewer:** So, besides the risk attitude, which I'm just mentioned. Any other things, reasons, you can think of that make the estimators make different judgments?

**Interviewee:** I guess what some of the other factors that would be, that shouldn't make a difference. But if you're an estimator and you haven't won a scheme for a while, you've had a lot of unsuccessful and you have rooms where that happens. I guess that potentially could affect your judgment a little, because you are kind of keen to, and you perceive to be obviously winning work is what you're there to do. So, if you've not won any work for a long time, you may well be more likely to take more risks. I'd suggest that would certainly be a factor, but it shouldn't do. And, certainly when reviewing stuff that the other guys do, I tend to, you know... it's sort of, we talk about holding your nerve and still doing the same things, the same process, but I think it would be remiss to suggest that it doesn't affect your judgment, because I guess you could. If you've not won a job and you never win a job in two years, you're probably not going to keep a job as an estimator.

**Interviewer:** Yeah. So, I think involved in many highways project and some projects they may be complex, and the context maybe is dynamic. So, can you take one risk, which worries you or make you difficult to quantify its allowance as an example and describe how you finally approach it?

**Interviewee:** Yeah, difficult… quite. I guess might be on a, we don't do lots, but we do do some on it, as I said some on a design and build basis. So, if you've got a relative, even if it's relatively developed, but that the design is going to be adopted by a local authority. So that will be design development to get that technically approved by the local authority. Assessing that risk based on the drawings you got it’s difficult cuz you could then do your estimate based on the information you've got, but what the local authority then will insist upon in the final design can be challenging. And I guess that'd be probably more, maybe more comfortable with a local authority, you've done lots of work with, so for instance, we do a lot with [council's name]. So, we're probably very familiar with their standards and things they like to see. And, if there's obvious emissions, he may well be able to take a fairly informed view on what the additional stuff will be.

But if it's the local authority where you don't know that would be challenging to work out what that design development, additional construction costs would be. And I guess you could look historically at it, but I think again, I think we probably generally want to qualify that if we could.

**Interviewer:** Ok. So, you mean you will pass the risk back to client?

**Interviewee:** Yeah. So generally, our first protocol would be to say, we'll price the the design as it stands, but any design development would then be treated as a variation. If it's pretty much, it looks like it's... I mean, you've got some, it's only minor comments from the local authority, I guess that the client may well then say, 'well, we want you to take the risk'. So. We'd be then if we say, if we're very familiar with the local authority and comfortable that they won't be adding… I don't know diamond crusty surface course or like… we would then obviously consider and then make an allowance for that design development. But that is a very... can be quite a challenging one because ... so sometimes we'll price and there'll be lots of iterations of a design. So, we might've looked at it from a client and priced it four or five times. So that probably can give you a bit of an indication of the state --what the cost was at that stage and the state under scheme may well have gone up 20 or 30% during those iterations. So that kind of see gives us an indication of how the cost can vary during that design development.

**Interviewer:** So, I know it's very difficult to make this decision. So well, for you, will you think about the consequence, or the effects bring about by your judgment? I mean, I know you're the estimating manager, estimators will report to you, and you will make the decision. So will you think about the consequence, the effects.

**Interviewee:** Yeah. So, we'd probably, obviously we try and then have a risk register with what the additional costs are likely to be and then say probability on it. That's how we try and then review that. And I guess, in an adjudication, you'd probably challenge whether you thought that was realistic. So, you wouldn't sort of hang just what estimator out on his view on that. So, you would particularly... it's depending on the magnitude of the likely magnitude of the risk. So, in the instance of that, he would say it was pretty much there with the design, but we thought there might be the odd tweak. It's a client or a local authority where we work a lot with, we might go, well, they normally insist on this, a different type of surface course. What's the extra cost? Likely is B for that and a couple of other bits and pieces. And then we might then go right that way. We'll put an extra… I don’t know… 20,000 pounds or whatever it might be based on a list of what we think those changes could be.

**Interviewer:** So, you mentioned that you will assess the probability of occurrence of that risk, so in specific how you measure it?

**Interviewee:** Yeah. And I think a lot of that we judgment, if I'm honest, it’s the likelihood of... going back to... we were talking about ground condition and having a soft spot part judgment. I mean, I guess with the soft spots, you'd probably be thinking in the winter were more likely to have more soft spots than in the summer, with that as an example. You would potentially look at previous schemes, we've actually undertaken, where the ground conditions were hopefully similar and how much extra material that cost you and use some of that historical sort of the knowledge to sort of cross check your assumption.

But it's certainly not an exact science. It'd be probably a combination of all those things… probably asking. In our office, it's an open plan office. You'd probably discuss it with a colleague as well and say, I'm thinking of this... do you think that's reasonable? They then probably also then look at what are their previous schemes and see how, what the outturn was on that and might say, well, actually I think you're a bit light because on project B, we expended an extra this much and he wasn't that dissimilar to that.

So yes, it's probably a lot of judgment knowledge, hopefully. And the say just, and then trying to, to tap into. You're the rest of that in the business or even to be honest, beyond the business, you might have other, depending on what the nature of the risk is. It might be that you've not encountered it, but you've worked with somebody, and you'd then use your, and you'd ring them up as well. And you might work with it in the previous business and discuss that with them as well, and get… and take a view from them and then just present... this is what we've done... are we comfortable with this? And then as a business, take that decision on the risk.

**Interviewer:** So, do you have any general principles or procedures, rules you can follow while make this judgment?

**Interviewee:** It really would depend. In all honesty, probably not really there... they're probably they probably are sort of banked in my head, I guess, in some ways, rather than being a specific... yeah, I, yeah... probably not. We've got, I mean on outputs and things like that, we would have, but that's not really a risk. That's just on the general estimate because obviously if every item you price, you'll have risk in there. So, and we would look at... we'd have rates for laying surface in rates, rates for laying type one drainage, output rates and things like that in different conditions. And we'll keep historical records of that to inform the estimate.

But then the extra risk on top of that, I guess is generally a little bit of judgment. But based on probably previous knowledge and experience and, where it's either gone right or wrong. I mean, what we do at the end of what probably aids that process, certainly here at the end of when we've completed a scheme, we'll have the estimator in for a post contract review and then we'll go through what went well on the scheme. What did work in terms of and then you'd probably go actually on that we were using a lot more stone than the estimate has been allowing. So that's something to take forward as a risk moving forward or consequently as we didn't use as much so you're being a bit conservative on that view.

**Interviewer:** So, would you mind sharing some of that like rules of thumbs, guiding principles you made for yourself from your previous experience that you always follow?

**Interviewee:** Yeah. I mean, I, for me, I think a lot of it's a process of just identifying… the identification of what the risks are first for me is. And then having that as a comprehensive list and understanding what's in your base price. So, I have allowed for 300 million of capping. I've allowed for that. And then having as a risk or an opportunity of what's outside of that. Absolutely keeping on top of that is still the principle for me. That's incredibly important. He might say the view slightly wrong, but you do need to know what you've allowed. Who, and in the contract, what, where the risk lies?

Because if they've admitted physical conditions and you've absolutely got to take it, then, you know, as I was lotus, we obviously ideally liked the risk back if we could, but obviously there'd be lots of examples where you can't do that. So, in which case, then this is why we have that schedule and as you're always pricing the works and doing your takeoff, going through the process of pulling together the estimate, always keeping that as a live document, the risks and opportunities that probably more than actually having a fundamental while I'll always allow this much for, but for additional, if we've got to take physical conditions. Because I think that's so scheme specific generally, because you know, if you're doing something in rock or you're doing something in a cohesive material it's wildly different. What you'd probably then just do is you'd look at some, try and find something with fairly similar conditions. So, I guess experience massively helps in that instance, but so having a good memory probably is a good thing, what going well, what did, what happened on that job, when we did that? And or knowing who did a job that was similar and then being able to... because we'll keep reconciliation of materials and staff by them. So. I'm almost keeping it on the capping, cuz this almost seems like an easy thing with the ground conditions... so... or contamination, I guess is another one... what, you know, where we have encountered that, what was the cost? But again, that could be, might be… that probably comes into what's very difficult to assess if you haven't got enough information in the ground investigation for contamination, that would be a very different risk to put a number to. Because even if you know it all, you could classify as not hazardous or contaminated materials, depending on where the license tip is it can take. You'll give you a range of prices per load to take that material could change from 300 pounds a load to 1200 pounds a load. It's just and that would be so specific. So, difficult to have a complete role for that.

**Interviewer:** Okay. Thank you for sharing about this.

**Interviewee:** Sorry. It's a little bit, roughly, I'm afraid.

**Interviewer:** No, that's good. So, I see this is a quite complex process. You need lots of information and think lots of things. So, do you use any software to help you make the judgement?

**Interviewee:** No. We price all our work in an estimating software package. So, I guess the fact that, you know, we don't just do it in Excel. It says that that will give you all the other quantities. And then you can use that software to price up some risks, but it doesn't actually generate a risk part. I mean, I guess you… I don't know if others do that. Some people might look at what contingencies they've had on other schemes and outturn costs. And then historically apply that. I just think that's incredibly difficult to do it because every scheme is so different. And there's just so many factors that influence. What the risks specific to that will be... what would amount to that? So, we use estimating software, but it doesn't generate… automatically generated a sort of typical risk pot or anything because I just... I'm not quite sure how you could do that, but...

**Interviewer:** yeah. So how do you think maybe in future one day, your judgment work, I mean pricing risk allowances can be replaced by some algorithms or some software?

**Interviewee:** I think elements of it could. As I say, probably that if you looked at ground conditions and you could probably put in various factors for what the material is that you're building the road on. For example, I'm guessing it wouldn't beyond the way of man to have the time of year you're doing the work because that would affect how the CBR values of the you're likely to have found the road on. You could probably put something in for that and your construction build up, and it probably could generate something for that. That would have... a more analytical, scientific element to it than the sort of like, our judgment.

The only thing I'm not sure I think is to input all of that, those factors in… might be the actual overriding factor that makes that slightly difficult to do it. So, and certainly, probably on the scale of projects that we're doing, if you're doing a 2-million-pound scheme, I think if you're doing a perhaps on a 200-million-pound sort of huge bypass, that would be viewed differently and at a previous company, a bigger company, they did that, not in the bit that idea, but they did use a lot of… they didn't have the design. So, they had sort of… big sort of -- the square meter of road is typically this much and put big chunks of assumptions in and then would have generated risk in a different way that we consider it. I don't know an algorithm for that would certainly I think, assists that.

**Interviewer:** Okay. So, I think I didn't make it quite clear. I mean, do you think maybe they can totally replace the human beings work? I mean, maybe one day we do not need estimators anymore. The computer software and the algorithm they can calculate, they can do risk allowance quantification.

**Interviewee:** I don't think so if I'm honest, but yeah, I don't see it. I think like, sort of defining sometimes there's the black art, cuz it's sort of like a bit smoke and mirrors. And I think that judgment is... I just think there's, so many factors to consider. I think it would be incredibly difficult. Yeah... i, yeah... it asked... a simple answer-- no, I don't think so.

**Interviewer:** What do you think... what's the differences?

**Interviewee:** I think because, well, time, particularly. I think sometimes you've got reams and reams of information, and you'll sometimes skim reading some of that to try and get an overall understanding of what the ground conditions might be, because obviously in a tender period, you might only have… you have a fairly limited time to try and have a real understanding of the scope and the risks. So, I think you're processing that information. You're reading it and probably doing lots. I don't see how we could give a a piece of software 2000-word ground investigation report and how it would then evaluate. And less and less other elements of the works information are done differently that I guess if you then were able to give some software, the ground investigation in a different electronic format that made it easier for the model to ... the software to also analyze that. I think it would need lots of changes to the how tender information is produced as well. Yeah. For the software to be able to do that. And then as I'm thinking if you're able to do that in a different way then it is possible... it's possibly feasible. And then you might in a way that perhaps we do our pensions or whatever, and you can put pensions in different risk profiles.

You could basically get that this scheme will be this and I'm happy for a risk on a scale of one to ten of six, for example, maybe it would then turn you out sort of what kind of cost, but I don't know. I think that's just even the component parts of building, the estimate would be difficult completely to be done automated because we will go out for quotes to the supply chain for every scheme. We'll build up all of the outputs. For every rate from first principles and that won’t get me wrong, but there might be. We'll have some, perhaps some library outputs that we'll put in, but we'll tweak them depending on the constraints for that particular project. So, all of that to be factored into a software, to come up with a price, I think would be … I think it would be very difficult. I think you could possibly produce something that then somebody would then have to go in and still make some manual adjustments at the very best I would suggest, but...

**Interviewer:** Okay. Thank you. Thank you for sharing your ideas and thoughts and you know, our topic today is about risk allowances, and it seems to be a concept, which is, I think it's financial, commercial related. So, when you pricing risk allowance, do you only think about your financial objectives or will you think about any other aspects, perspectives?

**Interviewee:** No. No. I will see... health and safety risks are certainly something that's paramount but also reputational risks.

So, I would say that's one where if it's a high-profile scheme and it went wrong. So ...sort of say a highway scheme in a city centre environment, probably with some public realm, suppose high profile. You're on this and you're all visible. It's that the contractor's name is on that scheme. Now it might be. There's been a design issue or another factor. That means that you're there longer than you would be. And all the businesses are up in arms and you're going to get some bad press from that. So that would be something we would absolutely consider is reputational risk from that perspective because it's not the designer's name, it's not that... they're not visible delivering the scheme, you know. The public's perception is, and the businesses and the other stakeholders' is the contractor. So, we'd absolutely consider that whip. So, once we were in [City] and the [Activity] are here next year. So, there's quite a lot of schemes that have to be complete, obviously head of that. Get that wrong and I'd suggest it's not just the finances that you'll... I mean, ultimately, you'll probably suffer financially as well through the reputational lost, but… so absolutely we will look at other risk factors as well.

**Interviewer:** So, will you take the, I mean, all the stakeholders and for example the environmental into consideration as well?

**Interviewee:** Yes. I see it. So, I mean, we have as a construction phase plan where you end up having an environmental risk register anyway, so I think but we certainly have to be a lane at tender stage identifying the main environmental risks, so that there's a discussed adjudication.

I mean, again in a previous company, we used to do quite a lot of work directly for the environment agency. So, obviously that would be even more key, but having a prohibition notice or something like that impose on the company. It would affect the business as well as obviously doing the actual environmental damage itself. So yeah, we certainly something we consider,

**Interviewer:** okay. I think that's good for your company to take all this into consideration. And so how about you? Do you think there are anything else which should be taken into consideration? I mean, for yourself.

**Interviewee:** Yeah, not really, particularly. Like I say, I'll be honest, obviously businesses are operated to make profit, but it isn't just about that. It's quite doing interesting schemes that how particularly if it's the local environment that is local to me. It's nice being involved in successfully delivering a scheme well. So, if there's a pro se a time risk and probably something that I sometimes slightly more uncomfortable with it. You might make a commercial decision because you're in a competitive tender that the information is not great, but you can shorten the program knowing something's going to go wrong and you'll get a compensation event for that.

But that means that the scheme will then go on for longer, but you might be covered and that it probably better if it's probably a failing of the competitive tender process in some ways, that the schemes, I've most enjoyed working on have been almost negotiated schemes with the client where you're able to be absolutely upfront with where you see risk of seeing competitive tender, that's more difficult. Because you've got, you're all operating, in a commercial environment where you're trying to secure the work. So, you might not want to highlight all of the risks to the client, but obviously if you're in a negotiated contract work properly in a partnership framework is more fulfilling for the individuals as well. I think works better in terms of managing risk.

So, then you're able to then have risk workshops. And openly discuss the risks with the client, and then you would be able to put more reasonable numbers in, regardless of who's holding that. Yeah. I think you're able to have those open discussions and say, look we see, this is the key risk to this and being less adversarial than perhaps traditional contracting. I think NEC has changed that in the last... so the 20 years, it's got better. I think when I first started in the industry, it was worse for that, but I think there's some way to go. And I think the problem in part is because clients obviously want, they want to go through a commercial exercise. So, they see particularly developers, a competitive tender as the way forward. I'm not sure that always gives them value. Suggest it doesn't. And actually, having that, you perhaps could have a first stage tender process that does that initial competitive nature. And then actually working in partnership with a selected contractor, perhaps with some agreed rates, some things and what not. So, you keep that, you're not obviously giving them free reign for their cost, but actually then you were collaboratively with them to assess risk. I don't think... I think actually just delivery of a project goes so much smoother when you were that way. That might not make complete hearts to your question. To be honest, I might've gone off on a tangent apology, but ...
