# Supplementary material for: Critical factors influencing cost estimators’ judgements on cost contingencies in highway construction projects: An empirical study in the UK
Source: PLoS One. 2024 Dec 16;19(12):e0314665. doi: 10.1371/journal.pone.0314665 (PMC11649144; doi:10.1371/journal.pone.0314665)
Supplement: S2 File — (ZIP) [file pone.0314665.s002.zip › Transcription (interview B).docx]

**Interview B-Meeting Recording**

**Interviewer:** Okay. So, would you mind to firstly, have a brief introduction of yourself, like what sort of work you do?

**Interviewee:** Yeah. Okay. I'm [name]. I'm an estimator, lead estimator for major civil engineering company [company's name] in the UK. Yeah. I've been involved in the industry for 35 years in infrastructure. I've been working on highway projects on and off for that time. I have not worked exclusively in highways. I've worked in general infrastructure. So, part of my working life has been in highways to date. Parts has been in other aspects of infrastructure, railways, water supply, sewage, power generation, other aspects of infrastructure, but a reasonable part has been in highways on some of the schemes you're interested in – these less than 50 million pounds at today's prices and also some larger highway schemes.

So, my involvement is always been with the perspective of the contractor with contracting organisations on site for maybe 10 years as a project manager, working on some highway schemes. And since then, in the last 25 years, I've worked on estimating, putting together tenders and more recently leading bid teams, etc, and more of a management role. But I was still very much involved in estimating and assessing risks, opportunities in associated with these projects.

**Interviewer:** Okay. So how do you become an estimator? Do you plan to do this, or you know, it just happens?

**Interviewee:** It's... yeah. Very interesting question. Very interesting question, cuz we are quite a small group of people. Within any construction company, you will have many project managers, deputy project manager, all involved in project management. You will have a large what they call increasingly commercial team. They used to be called quantity surveyors, but now they're a commercial team. So, we have a lot of them, but estimators tend to be a small group of people and how they get into estimating. There is no degree course in estimating or anything like that. You come into it and in infrastructure you tend to come into it like I did from the project management side of life. You, rather than the quantity surveying side commercial, it's more the project management. You're interested in planning, logistics, productivity, how you set out a site… planner site and then that leads once you've planned and program a site, you then in a position to price the works. So that sort of discipline of doing something.

And then there's quite a specific discipline is quite almost a unique way of working compared with others in the industry. If you're on a project, you're look, you're building your highway. You'll be there for two years… three years and you're working with lots of people over a long period of time. As an estimator, when you're pricing that highway, you're not working for two or three years, you're working for two or three months. So, it's very ... it's more a different discipline. Let's say that… you have to pull together and identify what is important to look at and what is not important to look at cuz when you have all the data in front of you for a 50-million-pound highway scheme, you can… it can be a little maybe overwhelming. You think, what am I going to do with all those data? What data do I look at? How do I look at the data? How do I do this in three months? It's going to take them three years to build it and I got to do it in three months. So, it's a different discipline. And that sort of discipline appealed to me, and it appeals to others. And that's we tend to come from project management background within contractors into estimating, but there is no ... it's not something you generally come in as a... let's say an apprentice or a junior, you tend to go to site and do project management and then come into estimating that way around... tends to be that way for civil engineering infrastructure is what I've experienced and it's how I got into the industry.

**Interviewer:** So, how you learn the thing that required for estimator? Do you do any training?

**Interviewee:** So, well, as I say, maybe it's important to understand how you put a price together. How would you get a price of 2 pounds 53? Where does that come from? So, we're talking about highway. So, let me be specific on highways. So, for highways, there is three elements of a highway where 80% of the cost is. Just as the road for putting to side once for more many structures like bridges and retaining walls if we're just talking about the carriageway.

Okay. So, the carriageway, the main elements are the drainage, the earthworks, and the pavement. And then, in the language of highways, the highways series in these countries, that's the 500, 600 and 700 series of the highway specification in the UK. So, these are the three main elements. So, you have your drainage. Your earthworks are principle. A road is about forming the horizontal and vertical alignment and then the pavements. So, for the earthworks, that's maybe one of the most complex things to price and you price it… you have a process to go through. And the first thing you do is you quantify. So, you have your horizontal and vertical alignment. You look at your typical cross sections and you say, right, I have got a million cubic meters earth to dig. And then I've got, hopefully it's balanced. There's an earthworks balance and I have a million cubic meters of earth to place. Okay? So, it's the engineers have designed the alignment well, and it balances cut fill… I've quantified.

Next, I have to classify. So, what is this material? Is it rock? Is it sand? Is it clay? What is it? So, there is then the estimator… wouldn't do this. The estimator would not do the classification. That will be done by a geotechnical engineer who would say this material is of this classification and is suitable for forming highways. This material is not suitable for forming highways and they will produce an earthwork schedule for the estimator. So, the estimator then has his quantities, his earthwork schedule. Now the complicated bit we have-- mass haul. I don't know if you've come across Masshole yet? It is how you identify where the earth goes in a roadworks job. So, I'm going to dig it from cutting number one. I'm going to put it in embankment number one. You will see a profile of a road and you decide where you're going to move the earth around on that project. Now, this is where the experience comes in. You decide, how am I going to move this earth? What machinery am I gonna use?

The estimator doesn't sit in splendid isolation within a contractor. We're not just a little box and nobody talks to us. So, we will talk to planners. We will talk to our project managers, and we will have a discussion. And we say, this is our project. What I think we will do is… we have a 60-ton excavator. We will have some... a 40-ton dump trucks and we will move the earth in this fashion. So, you put together what we call a fleet of earthworks equipment. So, you have your 60-ton excavators, 40-ton ADTs (Articulated Dump Trucks), compactors or whatever it might be, whatever you decide. You don't decide alone, you discuss it within the company, because this is a key aspect of what you're doing.

It will inform a large percentage of the cost of this job is your earthworks. So, once you've decided on your fleet to plant, next, it is how quick they gonna work. How were they? Were they going to move a thousand meters cube today? 1,500? It depends on the distance they're having to move it. If they do this a short distance, they do more. If they do a longer distance… and you do this study. It's fairly, it's attributed... try one, and then you try another and then you try another, and you come up with what you think is the best output, but there is no… here's my handbook of answers. There is no... it's iterative. If you talk to the other people and you come up with your output. Let's say an estimating... some of estimating is art. It's not a hard science. You just discuss and say, okay, I think this is something like this. So, there is no definitive. You've done a study at university, and you have all the answers? No. No. Each project is unique.

In the distance, you have to hold the material; the way you're going to do it on this job; the amount of suitable material you have; the amount of unsuitable material you have. So, you've got your fleet of plant. You've decided the productivity. You can then work out a plan. You can work out your plan, draw it out, using [software's name] or whatever software you choose to use. You can work out your plan, give you a program. After you've got your plan, you can then produce a program to base on, how many of these gangs you put together, how you resource the project.

At that point, you then have a list of all your bits of equipment or your labor force, everything else you need. You then cost that list. You put a... we have your excavator there for 20 weeks… your dump was there for a hundred weeks, whatever it might be. Your compaction plants, you know what these bits of machinery costs per hour, you hire them, you know, where, how much fuel and driver, etc. You cost all this productivity, you say, ‘right, on that program, you put the resources against the program’, and then you say, ‘well, that's a fleet cost me 5 million pounds for that time and I have 1 million cubic meters of muck to move’. As we say, therefore, it's five pounds per cubic meter. That is how I get to my five pounds. Where did the five pounds come from? That it is that process of pricing, a resource, which is the fleet of an equipment you will use for a duration. And then divide it back by the quantity of muck you're moving.

So that on highways very, very simply, very quickly is the one of the key things you have to do. So that is how we establish a price. Let's just say it's, not old science. So, it's intuitive. There's a bit of art you just say, I think this will be. This is from what I'm reading of the ground and what my colleagues are telling me. I'm talking to the planner ... the site manager. We come up with a consensus and that's how it is priced.

**Interviewer:** Okay. So, I think the cost of a project, it's not just the base cost, but you may also allocate some risk allowances. So, can you take one risk, which happens most in the highway projects and talk about how you make judgment on it. I mean, its probability of occurrence and its potential impact on the project. What things you will think about while making these judgements?

**Interviewee:** Yeah, well, there is on a highways project. There's many, many areas that affect us. As with all infrastructure where we are affected most is when we are in the ground when we are in contact with the ground. Cuz, we don't actually know what that ground is until we get there. We have a site investigation. We will have some ground investigations and trial pits and boreholes. We will think we know what the ground is, but until we get there, we never truly know. So that is one area of the risk is the unknown condition of the ground. And the way this is affected, by the weather. It's very significant because when we're moving all these materials, they require to be moved with a certain moisture content. If the moisture content is too high, usually you cannot compact the material. In simple terms, you can't deal with mud. It's hopeless. But that material could be fine, if it had a reduced moisture content. So, we know this. This is not news, not surprise.

So many highway schemes, we will identify what we call an earthworks season because we only want to move the earth at a certain time of year because of the moisture content. And usually this is simply summer and winter. When this varies, the season is obviously various as you go around the country. We recognize some parts of the country... there is a lot higher rainfall than other parts of the country. So, we have to look at each project and identify earthwork season. So, we say that we will not do any work in that out of season. So, in the winter we will stop the major earthworks. We want to do it. We want to attempt, we may, we may, we may find ourselves. We will price on that basis that we're not going to work. You may have a very rare condition where we have an exceptional winter and it's dry and we can carry on. Well, this is a bonus. The contractor is having a good year. And then conversely, some years you will have ...you were working in the summer and like, today, it's raining. It's the summer and we have a bad summer.

So, then you have... you don't move as much muck, as you thought you were going to move. It's not been good. These are risks which we... as I say, it's more intuitive with… and worked on empirical data. There's nothing hard and fast… there's no hard and fast fact on this. When we decided our fleet of plant would move… so many… thousand cubic meters a day or two thousand ... whatever we fixed on, between the planner and the production team and the estimator. This is just what we, a part of the art of estimating. You think this is the data, you have it. You have it confirmed when we go to projects. We do a little work studies over periods of time. When the plant is working and we said, yeah, okay, we've monitored this plant for a month, and it achieved this output. Within that month, it rained for four or five days. This is normal. So, the plant was stood. It didn't do any work, but that's normal. So, we look at a period of a month. I do a work study and just to confirm that we've got the right sort of data that we're using when we're pricing our projects, but the data is held internally within the businesses. It's not industry wide out there. People need us to say... a very precious about this data. It is commercially sensitive. You know what productivity is you're achieving and people like to keep this data to themselves. So, we do verify our data that we have. So that's how we get our productivity. It's that's how we do it.

**Interviewer:**  So, will you have a cost risk allowance for the risks you identified?

**Interviewee:** So, cost risk allowances on a project basis. We've just been speaking about earthworks. So, let's look a little wider for the whole project. So, for the whole project, my starting point there is my conditions of contract. My contract tells me what I have to consider. So usually, I have to consider the weather, like we've just been talking about for earthworks. And then it will list all the things that can... well, it tries to list it. Invariably it doesn't, but it tries to list all the things that the client wishes you to take on board. So that is my list. They will tell me… you need to consider the weather risks. So, I've done that. I've done an earthwork season. My productivity is based on what I think I can achieve in that part of the country…we... what we know about the rainfall, etc., and what we know about the ground. So, I've taken that risk on there.

And risk for inflation. For example, we may have to consider the cost of materials over the duration of our project, which may be three years. We may, we have to look... and again, just like we did with our earthworks. We just look at what's happened historically. What do we know historically on inflation? And currently in the news, there's a lot of discussion about steel, the price of steel in infrastructure and building. What I see on that is ... it's a commodity of course, and it is very cyclical. It's everybody is talking in the papers and in the media, in construction media at the moment about the price going up. When the price starts coming down, nobody will be talking. Nobody will be talking that the price is coming down, if this happens. So, you just look historically and say, well, over the last three years that steel prices have moved up and they've come down and we just assess where we think we are in the cycle. At the moment, absolutely, the steel prices are going very high. Do we think this is going on forever? Or do we think now as a business, this will turn, we are part of a cycle. We look back 20 years, and we go up and we come down, we go up and we come down. There’s a trend general baseline inflation. Of course, the government's target is to 2% per annum for the baseline inflation. So, we consider that. And any additional items we think that may affect our project in the short term, because we are only there for two or three years, and we buy most of our materials in the first year. So, it's relatively short time periods. If the project is big project, which you're not really talking about, but if it was a large highway project, say for five years, generally the government would take the risk on inflation. They wouldn't ask the contractor to take it for five years.

And then there is other risks, which we have our productivity risks. We have a risk of labor that we know historically what the labor rates. Can we get the labor? That's a risk. Can we get the materials? That's our risk. Can we get the staff, etc? These are all our risks, but we know we've been in business for enough years to know how to manage this. It's not a major issue.

The problem... the issues that are very, very significant and affect infrastructure... as a contractor, we don't take them. For example, we don't take the risks on third parties. Archeology is a big issue for highway schemes. So, before you start, your archeologists will as usually done some, a little bit of study, but they may want to do some more study and we've been finding this increasingly. And they do that with ... come along with their trials and they spend as much time as they need. They spend as much time as they need. So as a contractor, ‘what? You're going to be there for one month... so one year?’ It depends what we find. If we find something interesting, then we will carry on studying and do our work and it stops the contractor. So, for a contractor, this is... we cannot be in business where we are waiting for one year to start. So that is a risk that goes to the client, whether it's the government or the local authority, or whoever's building this road. Then there is other risks that we cannot take, like well, we increasingly were asked to, but it is a magic concern... is with the stands, the gas, water, electricity, all these statutory undertakers. Their infrastructure is usually... if it's not in the way, it needs some new infrastructure on the new highway alignment. There's a lot of interfaces with them... interfaces with landowners. Cuz we as a contractor, we don’t own the land. So, somebody has to do the compulsory purchase orders. There's lots of risks that take time. The risks, we have tended to be the ones that we can manage, which is the labor, the materials, the etc.

And another one that's coming our way more and more is design. The risks that the design is not finished. And we have to assess how much... what we think the design is going to grow and how will that impact us? So, we look at the maturity of the design... when we say, 'well, okay, we've just got a basic'... let me think of something.

**Interviewer:** Okay. That I think that's enough. Thanks.

**Interviewee:** That's enough for you. There's many, many, many aspects of that.

**Interviewer:** Yeah. So, to risk, how do you think your attitude to risk? Do you prefer to accept more risk? Cuz you know, more risk more profit or you would be more likely to avoid some risks?

**Interviewee:** Well, we take the risks... we take the risks. As a contractor, we take what we can manage, but we can manage, if we were in charge. Like I was talking a moment ago about the archeology, I cannot manage this. It's imponderable... the archeology. So, I don't want to take that risk. Not at any price. No. I don't want it because we're talking about small highway schemes, less than 50 million. They could spend one year doing their archeology.

**Interviewer:** I think it is how your company, or I say how your team will do. But for you, personally, for yourself, how do you think of your personal attitude to risk?

**Interviewee:** Again. It's the same. It's what I can manage. I can deal with it. Yeah. I'm happy. I get in my car in the morning, and I drive because I think I'm okay. I can get to where I need to get to. I know how to deal with the highway or the other road users. I feel okay. You put me in a place where I don't know. I can't control things. I think, no, I don't want to do it. I just don't want to do this. This is too many imponderables for me. So, if we can manage the risk, we take the risk. We take many, many risks. We have to organize all this labor and materials and plants and established them for a project. And then in three years time, two years time, they've all got to go. So, there's many, many contractors risk, but this is normal. This is our business.

**Interviewer:**  So, there are many estimators in your estimating team. So, will they always reach the same judgment on the risk, or their opinions will be different?

**Interviewee:** Yeah. There was quite a variance in... well, yeah, there is a reasonable concern. Let me say, let's go back a step. So, I'll check myself there. We work together because we're of a similar mind. You don't have a team of people, all of dissimilar minds, as you may have a chaos. It's rubbish. We all have a similar mind and a similar outlook and a similar understanding of industry. That is how we operate as a team. A team will not have operated if everybody has different and conflicting views. We challenge one another, of course. But we have a similar consensus, and we also work in joint ventures. Joint ventures in this industry is not unheard of. So, you will… companies will join together and then you see different companies. And in my experience, when I've worked in joint ventures, not just in the UK, but internationally. There is a lot of common consensuses amongst the estimators about the metrics we talk about... about risk percentages, about productivity allowances. It tends to be quite a consensus. Very few surprises, which is a reassuring. We've come from a different approach, but we come to the same sort of answer.

**Interviewer:**  Okay. Because people, you know, people, they are in different characteristics. I'm afraid you will have different opinions. And I like to know how you will finally, you know... make a consensus.

**Interviewee:** Yeah. Well, it's... I don't know. I can't, answer that, but as I said before, there's a little bit of estimating that the science and the clever bit is the art. And as always with the art, you cannot define it. It's just something that's you come to. And you think, yeah, that will be a sufficient allowance. There is... no. If you're trying to do something more scientific, it's not possible. We don't gather a great deal of data in this industry to apply some sort of statistical analysis to things. We just don't.

It's interesting on risk that you're studying, I think. It is a great area of construction, where there is very little data. As a comparison, if you, I don't know...something, insurance industry, you have lots of data for people driving cars. You have millions of people, you know, person this age, that age, living in this town, that town, you have lots of data. For construction, we have very little data, very, very little data. Other than the data that the companies keep very close. They keep them close to themselves. That's... if you like their intellectual property, we know this, we check this productivity as we were talking about before. This is the data we have… we keep it... we keep it very close to ourselves. It gives us our competitive edge. But the wider dissemination of this data is not there. You think… that's something that'll be interesting for your study if you're looking at risk and trying to find data it's....

**Interviewer:** You just mentioned that different estimators, it's highly possible that they may have the similar views. So, do you think that risk assessment process, it's more objective or more subjective?

**Interviewee:**  Oh, it is, is more subjective. We, as I say, we don't have enough data. There's not enough data together to be really objective about it, to know factually, this is correct. This is a great. There isn't enough hard data on this. No, no. So yeah, it's a more subjective thing. And we call it an estimate as well. It's important to remember that. Later we will come to the cost, which is a fact. The costs that scheme cost. The money has gone. It costs us. That's a fact. We are doing an estimate by its nature. The very best you can do when it's all complete. It is still an estimate. It is like the weather forecast for tomorrow. The forecast for tomorrow is my best weather forecast for tomorrow, but it is the forecast. It is not the weather tomorrow. It's the forecast. So, we always have a degree of uncertainty, and we know this. We know this is a construction company.

We know this, but we, we gain our confidence in what we are doing by keeping this small team, working with this small team again and again. So, when estimator number 1, 2, 3, we know what this person can achieve. We know how reliable they are. We know the way they work. We have one... three projects with this person or the projects worked out well. There was no problem and you go. So, it's you... you build your confidence that way, but it is. There is not a great deal of written hard data to support this. No.

**Interviewer:** So, to my knowledge, there are many people, they try to use some models or software to do the risk assessment. How do you think the idea that one day, your work, I mean the risk judgment work can be replaced by some algorithms or computer softwares?

**Interviewee:**  Yeah, it's interesting. We touched on it before. I think with, for me, with insurance, the analogy with insurance. To make computer useful and do some data analysis, statistical analysis, you need good data. You need something to put in. And this is an industry, I think we don't have a lot of good data. As to what do you feed in and how do you format that data and put it in. It doesn't exist. And until we start collating that data which is... There's a debate for me amount this because a lot of what we do in infrastructure is one-off. There is only one bypass around the village. That's it. It's a scheme, a little highway. Well, it's not little, it's a 40-million-pound scheme. We built it once and that's it. So, you want to spend a lot of time studying all that, all the metrics and all the data, and you're going to do it once. This is crazy. And then you're trying to compare this scheme with another scheme in the other side of the country.

I have found that we can exist and obviously we have... as contractors we've existed for years using this approach that we still use. That is more subjective discussion. There is no hard data, no great computer software giving me the answer and it's worked. And some of the companies where... then of course the companies, sometimes they don't work. They have not got the right people in place. And they for a combination of reasons, they fail. But the risk assessment is a key part of it. And it's an interesting area of study, if you're looking to apply analysis to it. It's very interesting. I'm not quite sure how you get your data. I think that is the challenge. It's not the computer software... that's no problem. The Monte-Carlo and all these other analyses, no problem. Finding data, that's very, very difficult, very tricky. And how you gather that data. It's that's going to be the interesting thing.

**Interviewer:** Okay. So, for your company, for your team, do you currently use any software?

**Interviewee:** We sometimes use a Monte Carlo type analysis for running risk analysis. Yeah. Here we have a qualified schedule risk analysis and a price risk and analysis. So, the program and the price, we just do a few scenarios. Monte-Carlo as it always, every time you run it, it comes up with a different answer. You run it at three in the morning and one answer running at nine in the morning, it's different answers.

**Interviewer:** How do you think, the answer given by the software and the assessments given by the estimators? How do you think that there are some differences?

**Interviewee:**  Yeah, it’s for me, the answer to that is the software side, we haven't got enough good data. The computer can only work with the data it has and it's working with data that is not that good. There isn't enough good data. So, the industry, as you probably know, is trying to do all these Monte-Carlo risk and other analysis on projects, which is all applying a sort of a scientific approach, a statistical approach to things, which sounds great, but where's the data. And that's for me where things are not working as well as they might at the minute, the data isn't there. So, if you haven't got the good data, you let us carry on with the method we have already used for years and years and years. It's not a hundred percent, but it's works to now.

So, I made a little... personally, a little nervous of these industry approach to use these computers for everything. Well, a computer can work if it has good input. As we say, rubbish in rubbish out, if it's as good input, then we may get a good output. But we need the input first.

**Interviewer:** Yes. Okay. So, cuz you have involved in many highway projects, have you ever encountered a risk, you feel it's hard, difficult for you to price its risk allowances and how you finally approach it? I mean, you have to take that risk.

**Interviewee:** Well, the idea that you say you have to take the risk. Yes, we have... we have in any business, you have a hierarchy of management to control things. So, the estimator will look at something. Let me think of something… let us stay without archeology. For example, we spoke about that before.

The client has asked me to take the risk on the archeology for this 50-million-pound bypass. We know from the study they've done. There are lots of Inam settlements in the area. There is a lot of historical artifacts. It could take one year. I don't know. It's a big problem. The client wants me to take the risk, estimate it, look it and think it. No. Too risky. We cannot evaluate this. We then go up the managements within the organization. And there is discussion about whether we can, or we can't.

Ultimately, if as an organization or any commercial arrangement, if you feel you cannot proceed, then you stop or you make an offer to your client ... make an offer. 'Okay. We'll build your project. It's 50 million pounds, but we cannot take the risk on the archeology', for example. Which we do not understand this, your archeologists may find a beautiful on-edge village that they want to study for a year, and this will stop our project. We can't do. We can't have this risk. So, there is, as we say, there is showstoppers. You come to something that's said, ‘no, we are not doing that’.

Other ones can be with the utilities. We've had projects... I've been aware of projects in the past where they want you to take the risk of that the existing lease was on a building type project, but it illustrates risk is that the local water supply was sufficient to provide the water to the new facility. Well, I don't own the local water supply. I don't know what they're doing with a local water. It's all something that's too much for me. It's a showstopper. So, the answer's no. So, your question, what do I do when... how do I price a risk that I can't deal with? I don't price a risk I can't deal with I don't not do it. No.

**Interviewer:** So how about that some risks, which bring more worries, but you may take it?

**Interviewee:** Yeah. Well ... every item for me, every single item, whether you're doing your earthworks or you're doing a drain, or you're doing the fence on a highway, everything has some risks in there. Everything. Can I get the timber for the fence that I'm putting down the side of the road? We have the answer to that. I think that is very low risk. Why do I think that is very low risk that I can get the timber to put the fence? Because we've been building roads for many, many years. I've been involved in the industry for years. I talk to my colleagues. There is never any problem with getting timber for fences on roads, but there is a risk. But I, from my experience and knowledge and contact with others is ...don't worry about it. You will get the timber. It's not a problem. But then the other ones you can think ... well, this we need to be careful here because this often causes us problems. This is often a problem for us. So, we need to be very careful. We need to make sure we have the correct machinery. If there's limited supply of the machinery, we need to talk to people while we're tendering.

For example, let me think. Maybe not on highway so much but yeah, on highway scheme let us think... supply of aggregates. So, this rescreen requires a million tons of aggregates. Where am I gonna get them from? I go and investigate the local quarry. It is a tiny little quarry. Well, I can't get a million tons out of that query. So where? Then you just have to investigate the surrounding area and talk to the quarry owners. Make sure they have sufficient capacity within their quarry to provide you a million tons over a period of two years or three years or whatever it might be. So, there is that type of risk that you are conscious. I need to get a million tons. That's a big figure. That's a lot of aggregates. Where am I going to get that from? Can I get it from the local quarries, or do I have to start my own quarry? Because we do that as well. We do have what we call borrow pits. You start your own local extraction if you cannot get it. So, you're aware of things that can cause problems called it risk if you like on the project and you deal with them.

**Interviewer:** Okay. So, it seems that risk allowances is a more commercial concept. So, when your price the risk allowances, will you only think about the financial objectives or you're having any other concerns?

**Interviewee:** No. Ultimately time is money. So, the thing that costs contractors usually it's time. You have this project and you thought you were going to be there just the short period of time and you were there a long period of time. This is the risk for the contractor. So, it's to do with the program. That's where your risk is, your fleet... earthworks’ fleet... is there longer? It's taking you longer to put up this timber fence. Everything is taking longer. Why is it taking longer? It can be many... there's many factors. With earthworks is mainly to do with the weather or you could have some issue with the supply of materials. You have a problem with your quarry, or there's many factors that affect the program.

And that's... so it's you say it's commercial. Ultimately everything turns to money because we're a commercial operation. As a contractor, everything comes to money, but it is driven by mainly time. It's the program that affects... that means we're there longer, which means that it costs us more money.

And as we mentioned archeology. Increasingly there's a lot of study done on the environment. So, the ecology in the area where the plants and the animals, there's a lot of study done on that. And this is obviously very relevant when you're building a road. Said linear asset is going, whether it's an urban road going through some part of an existing town or city, or it's a rural scheme going through the fields, the countryside, is always the ecology side of things that we have to study.

Again, the estimator is increasingly drawing on more and more, experts. So, does the estimator know all about all the ecology or the plants? No. We have an ecologist, and we have to go and talk to the ecologist and say, okay, how are we going to deal with these species, plants or animals that we have to deal with? How can we move them if we've got to move them or what have we got to do? And then we have to. Could you supply... produce a program and then finally the price. So, we are talking to those sorts of people as well. The role is getting wider and wider. We have to talk to environmentalist, ecologists, archeologists, and then there's...

We are increasingly looking at what we call social value. And this is the community which we pass through. And we are costing for what we have to do in the context of working with local communities and various initiatives that the client may wishes to do. And as contracting organizations we do as well. So, there's all those things to price.

**Interviewer:** Okay. Thank you. So, for you, what knowledge or skills, do you think it's most helpful for you to make these good judgments?

**Interviewee:**  What knowledge or skill? So, I suppose for an estimator, what an estimator has to be very, very good at is talking to other people. Because you have a highway scheme, as we said, it's 50 million pounds, for example. There's a lot of components in that scheme, a lot of components and can the estimator know everything about every component? No possible... No possible. So, they have to go and seek some advices. We were saying, talk to any colleges, talk to a material engineer about the ground conditions. If we're opening up a query ...geologists talking to many, many disciplines. So, if the estimator needs to be very good at communicating with people and drawing out the information they want and doing it relatively quickly. We usually have no more than three months to consider a project. Sometimes it's longer. So, you have to be able to... That is a key. What is key to being successful is being good at communicating. And then when you get this data back, analyzing it, looking at what's important and what's not important.

Not really. That second bit is an experience thing. There is just, you just know after... as with anything after you've repeated any tasks, a number of times. You get more competent in that task, and more experienced in it. Yeah. But that is very important. You have to talk to a lot of, lot of different people. So that's a key aspect for success in this business.
