# Supplementary material for: Critical factors influencing cost estimators’ judgements on cost contingencies in highway construction projects: An empirical study in the UK
Source: PLoS One. 2024 Dec 16;19(12):e0314665. doi: 10.1371/journal.pone.0314665 (PMC11649144; doi:10.1371/journal.pone.0314665)
Supplement: S2 File — (ZIP) [file pone.0314665.s002.zip › Transcription (Interview C).docx]

**Interview C-Meeting Recording**

**Interviewer:** Thank you. So, would you mind to firstly have an introduction of yourself, like what sort of work you do in your team? Something like this.

**Interviewee:** So, I am the chief estimator for [company's name]. It's a major construction company, one of the largest construction companies in the UK, and then the United States of America. I was solely within the United Kingdom. I work after the estimating for the highways business and the turnover of per annual is probably a billion-pound highway works for one year. It’s quite a lot. And we do projects from 25 million to the biggest, I've negotiated or tendered for the business is 1.4 billion or 1.2 billion. What is the biggest. I'm a civil engineer by profession… I’m a civil engineer and I've been in construction now for…wow, that a long time now… 47 years. Of which I have spent 30 years in estimating.

**Interviewer:** Yeah. So, you must be an expert in this area.

**Interviewee:** Probably. I've worked in the UK, and I've worked in a lot of African countries.

**Interviewer:** So, is what you're doing now the same as what you learned from school? I mean your major. Is it the same?

**Interviewee:** I think the basis… basics are the same, but it's I guess what counts now is probably my experience that in brain and experience that I use. But what I guess… what has changed over the past years is perhaps the move to digital era. With [A software name], we may now only get a 3d model and some schedule... And then a reference to specifications where previously we would probably have on memory maybe 200 drawings and a specification which was that thick... So, yes. I think if anything has changed, it's perhaps construction techniques. We've got a bit more ... what I'm looking for... slicker and obviously technology. If anything influences my role, or the estimated role, it would probably be the move to technology more than anything else that influences an estimator's role.

**Interviewer:** Ok. So, do you mean that you think your role or what you're doing maybe someday will be replaced by some computer software or algorithm?

**Interviewee:** Interesting question. There's a lot of good software and even though I'm 65 years old, I still get very excited by it. We could take the raw data from our historical benchmarking of our construction costs, and we can allocate those costs against… with the new model, a 3D model, i.e., Pavement curbs, drains, chambers, elements in construction... piling. And we can actually get a... what I always call.... I would call it the first POS cost. So yes, you could in theory, in theory replace an estimator with a very good database and a very good model and also using a coding system. That's all you need.

However, however... this a big, however. Computer does not understand intricacies. There are a lot of intricacies around, so a lot of benchmarks or based support averages. So, the degree of difficulty is not always there and the feel for what we're talking about now -- the risk involved with some of the processes. A computer doesn't know because it does vary from project to project -- what percentage of works you may carry out at nights or weekends, especially on highways where you have to carry them out on the closures where there's less traffic around. A computer doesn't understand that. You could model it. Yes, you could model it in. But I think ... maybe in that years you can, but at the moment, I don't believe that we have the capacities. So, you could get a computer and a whole lot of data to do what I would call the donkey work for you. But the intricacy, in English what we said the devils in the details. Yeah? The devils in the details. There, wasn't a detail in little Tronic world, I think at this moment in time would best to understand.

So yes, it is possible that estimators could be partially replaced by technology, but maybe because I'm old fashioned, there's nothing like a bit of experience to look at something and to say "yes, that's standard. Yes. That's complex. Yes. That's got this degree of difficulty".

And you want to talk about risk. A lot of risk is around that or it was about understanding the intricacy of the process that you're undertaking on a project or the activity and the external influences that would be upon that. That would drive risk. Because one could say that we're gonna build a highway and we got to [unclear] the highway and we got to put in a treasury management system. We're always gonna work behind barrier and we're always gonna have the traffic grants through there at 40 miles/h. Risk... well, the risk is that not everybody travels 40 miles/h, and you still need to get in and out of your road works. The risk is that you may inhibit a road closure where you need to completely close the road to do something that crosses the highway, i.e., Put up a new gantry sign or put up a new gantry signal, whatever it may be. That's a generally done under a full closure. The risks around those are ... maybe an external event, which you don't know about that. They would just allow you to do the closure, and that would lead to a delay in your program. So that would be a time risk rather than a physical cost risk or you get affected by adverse weather. When you want to do your closure. Again, it would be a time risk rather than the adverse risk to your direct costs.

So, that’s the intricacies I think that perhaps the experience of people and experience of a construction team working together, analyzing the project would be over and above the standard; there's my model, there's my code; this is equal to that. That's fine. Except to that, it's better that, you would have to still have a team working in the background and all the intricacies that goes with it.

**Interviewer:** Thank you. I realized that you mentioned several times about, you know, the concept, experience, but I'm very curious to know what experience? Can you say it more in specific?

**Interviewee:** Based on experience... it… I think when the more you get involved, the more you do, the more you learn. And, and you use that learning experiences, sometimes good, sometimes bad to inform the decision-making process in yourself. And so, when I referred to, in my experience, it is what I've learned along the way, what works, what doesn't work, what is good, what is bad, and what can occur, what occur. Because often as a junior, you often might, or a junior estimator might miss things because they haven't had the exposure.

So, when I say in my experience, that's what, I mean, what I've learned along the way and application of that knowledge.

**Interviewer:** So, you've mentioned that you think experience plays an important role in your judgment. So, do you think that your experience can be taught to some novices in this area?

**Interviewee:** I pride myself possibly. And I don't know if this is, you might think this is being a little bit break heart. I ... in our business, I made most of the new estimators who come through our system or what we do is we actually bring young engineers and young commercial team through our system. Some of them work for us who work in estimating maybe for one project and say that they don't want to do this.

There are others, I have a very, very, very, very good estimator at the moment who has been recognized as a business's future leader. He worked with me since eight years ago. I think it was first. And he has an interest in estimating and he has a lot of understanding of the construction process and we have developed him through, or I have developed him, or we, we as a team business, I guess have developed him into perhaps one... perhaps a few years time being my replacement within the business. Does that answer your question?

**Interviewer:** Yes, thank you. So, in highway projects, could you pick a one risk which happens most and talk about all the things you considered, goes through your mind, while you're making judgment on it? The judgment I mean is the probability of occurrence and maybe the potential impact on the project. What things you will thinking about?

**Interviewee:** Yeah. Okay. I'll give you two examples. The first one is that if we're on perhaps a design and construction program or project, one of the biggest risks to us, as a contractor is a non-performance of our designer and having our designer design to budget.

So, what quite often happens is in a design team, they will always.... ask if everyone understands the standards. The standards are to build a product that lasts a certain amount of time and has the requirement to meet the design specifications, as well as the material specifications and the safety requirements of not just the construction teams, the safety requirements of the road user. Ultimately the road user is the client to either a local council or to major highway provider. Road user... that are their client.

And biggest risk to them is.... well, there are two risks ... that always occurred to... I will say... I will always look at… one is the lack of understanding of the budget by the design team. And that's where I mentioned design to budget rather... Because It's easy to choose the most expensive option. That's the easy rider. So, if I'm designing… if I'm a designer, I'm designing a road. I made the design to 25 years... It has to 25 years last. Well, just make sure I will make the design to 25 years last. So, these are risks that, we, as a business would identify very, very, very early in the tender stage. And we would mitigate that by having a robust design reification or process a place whereby we as a business together with the designer and the client would rectify the design and check the design for performity but also for buildability and for economy. So, that solves a massive risk because we have standards. We know what the standards are, and we would possibly base our base price upon a historical project of a similar nature.

Personal designers are old, and I need that being 250 millimeters instead of 200 millimeters. Why? So that we'd always have a look at the risk and what we call it -- scope creep. I don't know. On a project, historically scope creep has been between 3% and 5%, historically. So, you can't identify that, and you can't measure off your drawing because your drawing or your basic design as I may say, 200 millimeters, but for scope creep, it may change to 50 millimeters. Or you may have 5 kilometers of drainage that may creep to 5.5-kilometer drainage or your average pop down to maybe 300. Then it may creep to 375 or 450. So that are all... the risks there... we name it as a scope creep and to mitigate it, is having a good design coordinator on your project and ensuring that you work pretty closely with your design team. So those are mitigation risks.

Now we may say that the average scope creep on a project is 4%. So, we would take 4% of our construction value and add that to risk. And we'd say, well, what's the probability of that. And perhaps sometimes the probability we would base upon our experience of working with a particular design team. So, there were some design teams and to be frank, I’m a frank person. Some design teams I've said ridiculous that's scope would be 6%. Others as we know it would be... I don’t know 2%. So, we would perhaps say if it's a business where we don't have a lot of faith and we know that we get scope creep with it, we'd put a 90% probability. But if it was someone that we know we can influence his work and might be closer with, we may put it in a 50% probability. And again, a lot of that is based upon our experience. There are various businesses and sometimes that's right and sometimes it's not because risk is very loose saying, risk.

It’s not… you can’t… risk is… I would say that risk is an assessment based upon the experiences of all the contracting team. If you ask me to define it for you. That's how I would define it. Because if you have a very very raw team, they might not be able to identify the risks or they may overstate the risks and the trick is to get the correct level of risk. So that number one, you can win a tender, because you got… I don’t know... let's say you have an 8% risk pot, and you could put that as a 4% risk pot. Let's say the process he does. And you are delivering at a very same or similar rate of productivity he does. So, straight away, you're at a 4% disadvantage or at the tender stage.

So, that's a major influence on pre-construction and also within the construction period and during construction, I think one of the biggest risks is its action-- non-performance of the teams, to be honest. That is a risk that you control. So again, with as a business play, we are very good teams and we have a business believe that that we can deliver. So, we put a very, very small value on that. The others would put a much larger value on it.

And again, the biggest risk during construction phase depends on the project itself. So, if you were building a bridge across a river, probably your biggest risks would be flooding. That would probably be the biggest risk, which would lead to damage of existing works or delays to the program. So that would be a physical cost or time related cost. If you're building a road, I think your biggest risk is probably your ground conditions. And again, how I mitigate my ground resistance, how do I mitigate it by spending a little bit more money on my ground investigation.

**Interviewer:** So how do you think the ground condition, the risk of ground condition? Is it more controllable or uncontrollable for you?

**Interviewee:** I think it's controllable through spending money on geotechnical investigations and again, having experienced team around you.

**Interviewer:** So, if I suppose that the risk of ground condition becomes uncontrollable, do you think your judgment on it will change?

**Interviewee:**  Ground conditions is one of the largest reasons … or was, I don’t think it is a problem any more… one of the largest reasons for contractual claims at one time. And it is a risk that a lot of contractors, such as ourselves take to a certain extent. We will base our risk upon the information supplied to us by the client. So, if the ground varies tremendously from that, which has been given to us by the client, we always believe that's a client risk. So that wouldn't be as a contractor's risk. That would be their risk. Our risk is that if we underestimate the ground conditions, or we don't interpret our reports correctly, or if perhaps you may have a case where you have to modify your soils or you know you're going to have to modify a soil source by information client gives you, or you know that you're going to hit a contaminated area within your works. That opposed to be good examples. So, in treatment the source, you would attend a stage, putting a treatment strategy together-- this is what I'm gonna do it. Your risk to that is... do I have enough material in to modify the material, to reach the specification I want. And so, you would look at that and again, you would, as an estimator myself, I would have a good feel for it. One of my younger estimators may not have such a good feel for it, but I would expect him or her to be able to approach either myself or approach somebody in the geotechnical team for advice. So, a big thing about risk and a project is to understand what you're doing and to understand everything. And that is very important, hugely important because if you don't understand what you're doing, you can't assess the risk because you just don't know the extent of anatomy that I alluded to that earlier.

And again, at the moment, another big risk at the moment is inflation, at the moment. Now this is the thing. Risks change from project to project, but risk change was tied. So, in the UK at the moment with Brexit, and obviously with the influence of the coronavirus COVID, it's led to what I call artificial inflation. So, I guess at the moment, historically inflation has been around in construction… inflation has been around 3%, I would say. If we, as a business are taking inflation risk, a lot of clients ask us to do on short-term projects. We would assess the risks. Okay. Historically is 3%, but we have this artificial inflation and that official inflation-- it's not something that is tangible. So, it becomes a risk. So, say you would, again, and as purely, and again, a lot of that is, or I know that scale is gonna go up. So, we would assess it and say official inflation is going to be 2% or 3% above normal inflation and the currency is very high. But how would we mitigate it? We would talk or early days we talked to our supply chain to find out from them, what they, what they're finding in their market.

So, it might be, we're building a bridge. It's got a lot of steel lights. We speak to our steelwork contractor cuz that's one of in the UK that is taking off. And, and he would say, “Ooh, boom. I think, yeah, 3% is never gonna cover us. We need 5, 6% on that”. So, we know that that portion of the works, which might be 10% of the work is gonna attract 4% extra and then we'd speak to the, to the earthworks contractor and he would, or earthwork team, we say, “well, our biggest thing at the moment is fuel and fuel is 20% of your cost. So, you take an extra percentage by... I sincerely take 4% of a 20%. It would be 24% and you're dead that you are failing to finish. You put all that in a big pot, and then you would have a look at the possibility, all of the currents of that. So, and that's artificial inflation, which is a risk, and I can also bring it on by scarcity material.

So, there's a lot, there's a lot of things out there... that are. So, those are probably generic outside of, outside of that… that you would take from project to project and through experience, you know, you need to be looking at these risks and your project risks or things like your ground conditions, your design, your design team, weather. If I'm doing a project in the north of Scotland, my weather risk is higher than if I do one down south of England, the weather is very different. So, the weather risk application would be quite different. I would assess that risk by using data for weather stations because the client we'd say we normally get 12 days, but over the past four years, we've had 16 days. So, let's have four more days as an entire risk pot. So, you don’t add it to your program. You put that into your risk pots because it's not, it's not a tangible thing. So, risk, in my mind, is not, not tangible. It's not, it's not, it's not, you can't touch it. You're going to feel it. You can't see it. You could only understand it and have a gut feel for it… for it’s going to occur.

So, we as a business or as an estimator, I would ensure that we have regular risk workshops. So, with workshop the risks with, we may have people who are going to deliver the project that we call the delivery team. So, we have a delivery team with us, we'd have the designers in with us and we may even have the client in with us and we would say, what could happen on this chart? Here's what we're going to build, what can occur? So, we'd often just get posters. Yeah. Poster stickers. Yeah. And everyone would just write down all the risks and thing, and then we would group them together. And quite often you find that a lot of people are think the same and then you get somebody who brings something. Wow. Wow. Wow. Wow. We never thought of that. I never thought that as an estimator. So, I'm really, happy that someone from the delivery team brought it in and he may say “on a project, we did that same activity and that was a problem we had”. So, two ways, did you put in your risk register, or you price it? Yeah. If he, had it on his project, but we know to other projects we didn't have it. It may, we may assess it be a risk rather than the cost. So, I'm wolfing, I think.

**Interviewer:** Okay. Okay. Thank you. So, since you are the chief estimator in the company, do you have to do a risk pricing by yourself or, you know, the other estimators that they will hand in to you and you're just, you know...

**Interviewee:** Both, both. Yeah. I often, in fact, today I have a risk workshop for a major project we're working on, so we have a risk workshop today with the delivery team, the design team and the client. And during that process, I will have input into the costing, and I will probably agree what should be the correct level of value against the majority of the larger risks. So, I do do both.

I'm... yesterday I had a… what we call it a governance meeting. That's where the senior leadership of the business goes through the work that myself and my team have done. And one of my estimators had done the risk register. I had gone through previously with her. Then we went through it with the director. So, there was a case where the estimator with the delivery team had put together the risk register and priced it. And I just gave it what I called the 'sense check'. And then today I will be much, much more hands-on on the one I'm working on today. So, it's a bit of a mixture of both for the most of the projects.

**Interviewer:** Okay. Thank you. So, you know, I have many questions for these two different forms. So, for the, you know, you have to do the risk pricing by yourself, and so, you will have some time to have different opinions with others on the risk assessment. So, from what perspective, you will argue for, justify your own judgment?

**Interviewee:** Ah, that's a good idea. And again, I would probably say a lot of it is based upon the knowledge base, and often a… a delivery team might not quite understand cost. So, ideally, when you… when you're looking at risk, it's good to have commercial people, programmers, or planners rather than programmers ... planners, the delivery team. And there are the estimators who should actually that time be, influencing, which whether the discussion should be going. Again, based upon experience. So, I guess a lot of it, is listening to people's opinions and acknowledging sometimes I don't know the facts, acknowledging some of the facts that the delivery team may got.

So, I never believe, that a risk register should be processed on isolation. It should be processed as a team and the... I guess the minimum, maximum or likely should be agreed, as a team. Sometimes, as an estimator, you… you have to influence, because sometimes the delivery team might try to be over-protective to try and get the budget or might underestimate and saying,' no, no, no, no, don’t worry, we can do that'.

But you know, if the risk is alive, it's a risk. Yes. You might be able to mitigate it. Yes. You might be able to bring the cost of it, through mitigation. That we handle separately because you have the cost of the risk. Then you have the mitigation, mitigation measure costs money. I will give you a final upon, whatever of how much money should go on the risk and how much money should go on the cost.

**Interviewer:** Okay. Okay. Thank you for sharing. So, you know, for the other way, I mean, the estimator they hand in their judgment to you and you do what you just mentioned 'sense check'. So, do different estimators they may make different judgments on the same risk and under the same condition?

**Interviewee:** Yes. Yes. And again, I think it's personality sometimes and experience, and it's an experience. I may look at something, I think there's no risk there because I've seen it and I've done it. While one of my youngest estimators may look at something and say, “oh, that could be risky”. And, A risk, which they would name may occur, which I would not name it. So again, I think you just say it… it's a bit of a personality or you risk averse, you're not risk adverse or are you ... Do you have a lot of experience or a little bit of experience, and those do play a lot into, to understanding of project because the understanding of project, if you couldn't understand your project really well, you can't understand the risks.

And what one must remember as well, that there's positive risks and there’re negative risks and negative risk being an opportunity. How can I do this better? What in this design can we do better to beat and how do we do that?

**Interviewer:** Thank you. So, how do you know whether your team do a good job?

**Interviewee:** As an experienced person, I could generally look at a project and think... and a lot of this depending where it is design wise or perhaps through what the geotechnical conditions are or the exposure to traffic or whatever it might be. You get a feel for whether risks should be. So, a job that's very, very simple, easy to construct, and has a very good design, you would know that you, your risk should probably be run about maybe 4 to 6%. Where a job, very difficult traffic conditions, maybe various geological conditions, huge restrictions on access. You... although you'd pick it up when you price your work, that risk is still... it's still inherent there. So, that... a project like that may attract a higher feeling that the risks... that it is a risky job to the business.

So, again a lot of it based on experience and as well, and that's how our senior leadership team view it. They will have a look because they also... I guess the senior leadership team has the final say, although quite often they'll say, 'yep, we were happy with, with how you've worked to that'. So, the... a lot of is that feeling, but you can look at risk generally and, if you read it, read what the risk is, thinking your mind. Yes. That that's what could occur. That's what of my costs me. So that risk. So, I would in my mind and sometimes a little bit of paper, really look at the article... probably the top 10 risks, cuz that's where 80% of your value generally sets. I would look at the top 10 risks, assess them and see if they're in the right ball. And if they're not, then I would discuss it again with the estimator and I would ask him to call a meeting with delivery team and try and find out what their thinking was when they price it, because they may have a different answer.

So, that's how I would check it. So, yeah, look at it, have a feel for it and read the risk and on a paper sheet process, or on a spreadsheet process. And then, and that's how I would check it. Yeah, as they, you know, sometimes it's quite easy in that we may undercut... I don't know, we may have to, I would take 20%. Well, if I know there's a million cubes, that's 200,000 cubes and another, it might be about 66 pounds. So that's 12 million. Yeah. So, the risk is about 1.2 million. Yeah. So, some are easy. Some are more difficult.

**Interviewer:** So, what... depends on what you think it is difficult or easy?

**Interviewee:** On the complexity of the risks.... are very difficult risk... the high probability risks are probably easier to price than the low probability risks, in that...They're probably more explorative by the team and had written out. So, that's probably the easier to understand and price it. That very difficult... or a lot of difficult reason.

I'm trying to think of one off the top of my head quickly here. That might be an example to use... No.... I think one to be honest that's difficult to price. But I... you know, yeah... It depends on the complexity of the risk. Does it... some are easy that they would perhaps just have a pure cost implication. Some are a little bit more difficult. They may have a cost and time implication.

**Interviewer: Ok.** Thank you. How do you think your personal attitude to risks? I mean, do you think your attitude to risks can affect your judgment on risks?

**Interviewee:** Yes. I think, the, all... the all-group people out there who want to ignore the risk, bear with me. And then they might be incorporated to team. So, they will play down at risk with, and every... and then you have a person in the team. So again, it's probably very, it's probably per person should attribute to that. A very brave person is probably less averse to risk than a very conservative person. So, if you have a mixed team of, yes, some people were very bright, very outgoing. You find that they are... their attitude to risk is, “oh, don't worry, we can sort it”. And if you have a very conservative person in the team, they'll say, “oh, I don't know that that's going to be a problem. That's going to be a problem”. So, you have these traits within a team and again you're also... With my estimating team I have some estimators who were very risk adverse, always. Yeah. And I sometimes count my blessings and that I have them because they will.... I can't for... just about every risk you could think of when you're doing a risk register or working in a team with a risk register.

**Interviewer:**  Okay. Okay. Okay. Yeah. Thank you. So, from your own perspective, how do you think of your risk attitude and how do you think it will affect your risk assessment?

**Interviewee:** Okay. So, you're asking me from my perspective, a personal perspective, rather than a business perspective, how I view risk.

**Interviewer:** Yeah, yeah, yeah, yeah. I mean, so, I mean, are you the person, you know, more willing to take risks or you think you are more conservative?

**Interviewee:** I... I am not the most conservative of estimating managers. Okay. I will say then... I am... there are... I'm not conservative for ... when I look at the risks. I tend to look at on the optimistic side. So, I will look at a risk and I will look at the mitigation possible to mitigate that risk. And that, so I would probably say, “yeah, that's a risk. That's great. That's great. But guys, what can we do to mitigate that risks? And what can we do to bring down the cost salaries?” Is that something we can do with the our direct costs in our bid that we don't have to worry about that risk, or we reduce the cost of that risk. That's how I tend to look at it.

So, I'm again, I'm... Yeah, I'm bit of optimistic, but when I look at it, I would tend to try mitigate the risks earlier, then say, “yeah, it's a risk. Let's throw the money at it. Let's put the money into the cost and reduce the risks”. So, I guess that's maybe a little bit conservative cost-wise and a little bit optimistic on the risk side.

**Interviewer:** Yes. So, I think you have undertaken many highway projects. Have you ever encountered a risk, which you think it's hard to quantify it? And can you describe how you finally approach it and how you finally make the judgment on it?

**Interviewee:** Okay. So the question is have I... have I ever found a risk that is very, very difficult to quantify.

**Interviewer:** Yes. You don't know how to quantify, for example, it's probability of occurrence or the allowance for it.

**Interviewee:** Yes. I, I'm trying to think of an example to crunchy here.

I probably come across some many times in my career, many many times. Maybe a couple of examples. One example, I was working in a... for a French business. We were working in the Africa, and I was pricing a major project there. The government was slightly unstable. So, a huge risk to the project was that the collapsion of the government and that wouldn't... or that would lead to two things-- instability in the country, but also the non-payment to the business.

So, the non-payments in the business. Because we work in that country, we couldn't insure, so as an uninsurable risk, that we had to take. So, they got to say, what happens if it happens? And part of the problem is when does it happen? So, if it happens at the start of the project, your risk is probably quite small because you're just starting, you don't mind. But if it happens when you peak, when you're doing your peak work, it's gonna cost you more money. So, what do you do? Do you take the average? You take the most? When do you take?... So... yeah, so it ... so, what we actually did is we actually worked at a low-, medium- and high-cost value and then we took currencies against it happening at those times as -- it happened; the project went fine; there was no problems in that country. So, that was very, very, very difficult risk to actually put a price to. It was really difficult as well as being a difficult situation, to find a business. And to be honest, it wasn't a project with much much much more than 50 million pounds. But anyway, so that is one.

One another risk that I found difficult to quantify was, and it's on our current project. We were in a design and construct project, there was going to ... We were informed that there would be a change in design standards. They don't could tell us anything about, but they ask us to make an allowance for. So, they actually said, “well, that changing design standard may bring about A, B, C, D, E, and F”. I say, okay, and may bring those about. So, what do you do? Do you do take occurrence to change as I said. I and say, well, that would change my design by this, and it cost me there. Type B there and C that D that, E that, F that. To me, it was a very, very simple solution. So, I asked my designer, if A occurs, what happens, and I can... try put some money to that. B happened. The problem was that the designer didn't know the application of the design chain because it was still in the process.

So, what we were trying to do, we were trying to price something that wasn't tangible. There's no, there was nobody understood. It was a huge risk. It was actually messy. And what we actually did at the end of that, we priced it, but we price it at a very, very, very high value. And then we told the client what the value we thought it was and how we got there. The client then said, well, that makes my project unaffordable. So, we said to the client, well, if you could tell us more about the risk, we can, we could take it. If we don't understand, if you don't know what that standard is, why don't you take the risk? And we all sorted out during the course of the project as a change order. So, at the end of the day, that's what actually happened on the project. And actually, the client, we actually delivered it for, luckily that we thought the risk was going to be, but it was something that was just totally not tangible. It was just almost a possible price because nobody understood what we had to do, how the scope would grow? No. Nobody understood that.

**Interviewer:** Yeah. So, do you think this risk pricing process, there are some universal or general principles or procedures you can follow or not?

**Interviewee:** I think the basis, the basic principles of identifying risk is easy. And I think we as a business, have a system. We will... during the tender period, we will assess our risks by doing risk workshop by having the correct people in the room, big, big, big, big part. But sometimes you have the correct people, and you might bring in an external influence of people we don't know. And again, that's just so long to get experienced, but.... so, it's to assess the risk at the early stages, identify what your mitigation is and then to... I would say when you price a risk as gonna to occur, you could price a mitigation and say, okay, by spending that money on mitigation I told, I reduce my risk. So, the process is, identify the risks, identify where possible the mitigation… the mitigation measures.

So, yeah, and then you risk register must be a live document because it changes. So, during the tender process, which might be 16 weeks. It might be dependent on a project. It might be six months, but as most tenders around 16 weeks. During a 16-week process, that document is live, and it must be kept life from almost from day one. It must be a live document that can be accessed and understood by everybody. So as perhaps, as we all ... as you process your project, or as we speak to our supply chain, more risk get identified or risks that we want identified might not actually be risks. It might be fine. We might be able to say, no, that risk disappears because we've done that. So, risk would totally disappear.

So, it's all about from day one. You need to start your risk register and you need to keep.... it needs to be a live document all the time until... cuz you particularly talk about estimating, until the tender is submitted and once a tender is submitted, it needs to go and be a document given to the contract delivery team and it needs to be explained to the contract delivery team.

**Interviewer:** So, because your judgment may have some indirect effect, maybe on other people or on the society, something like this, will you think about this or have some reflection on this?

**Interviewee:** Yeah, I see what you're where you're coming from now, I guess. The thing is when you're assessing risks, it's not always the... it's not always the financial application on a project. It's not always about that. It may be ... how.... if the risk occurs, what would it do to... I don't know... the environment. What would it do to the road users? What would it do to the local community, about where you're building something or doing something?

So, you don't just look purely at that. You know, there's financial to be looked at, the safety aspects, there's environmental aspects. And then there's the environmental aspects include things like noise pollution, like general pollution, access need risk for the general public -- if you hold everyone around… it's… it was a bit of a problem because you always, generally, we'd always be cutting through an area that's populated or not so populate. It will generally have a public right of way in it. So, when you're looking at your construction methods and your risks and your opportunities, you always need to be assessed that. You wouldn't say, well, I've got to build a temporary footbridge, so people get over my works. But opportunities... I don't... but my opportunities, I'm not gonna put it in. And people could walk two miles down the road and two miles back for months. No, that is not. So, that perhaps looking at a negative risk opportunity against the ethics of it.

And I would... I've looking at contaminated ground. You know, when you did contaminate to make ground, you may say, well, yeah, we're gonna have a bit of vapor that comes out, but how would... that doesn't really matter, or do you put a control environment around it to protect with extraction with filters?

Yeah. So those are all things we look at when we're looking at the risk and also when we're looking at the buildability in any construction methods. So, they're all very intertwined with each other. So, yes. So, when you're looking at risk in, you need to not only look at the financial application, you to look at the, the whole application of everything upon the environment and community and all the stakeholders, all the stakeholders need to focus.

**Interviewer:** So, I think you just described how your company's risk assessment process. So, do you use any software during this process to help you doing the estimating work?

**Interviewee:** Yeah. So, for risks, for risk itself, we as a business, have a piece of software we do use that takes ...we take it from the cradle, i.e., when we tender the job to the grave, when we closed the job down. That risk... the risk restraints in a piece of software is live all the time. We use a bit of software in the process and the software is name in our business called it it's called risk.

**Interviewer:** Okay. So, for yourself, what's knowledge or skills you think are most helpful for making good judgment, well make it, maybe more accurate?

**Interviewee:** Having a bit of an analytical mind, perhaps. Yep. And that's probably, I think a good strain being able to analyze the risk and take it. What I will say, you need to take the risk apart. You need to deconstruct it and analyse your mind. What does it do? What does, if this risk occurs, what does it do? Does it change my program? So, it's a time risk or does it change a program and have some financial application upon actually construction prices. So, it is very much... risk is a very analytical thing. And I think perhaps the people who can price risk the best or the ones in my team were very analytic.

I myself, I think I've got a fairly analytical mind.
