# Supplementary material for: Critical factors influencing cost estimators’ judgements on cost contingencies in highway construction projects: An empirical study in the UK
Source: PLoS One. 2024 Dec 16;19(12):e0314665. doi: 10.1371/journal.pone.0314665 (PMC11649144; doi:10.1371/journal.pone.0314665)
Supplement: S2 File — (ZIP) [file pone.0314665.s002.zip › Transcription (Interview D).docx]

**Interview D-Meeting Recording**

**Interviewer:** Would you please to have a brief introduction of yourself?

**Interviewee:** Okay. So, I'm [name] I'm a senior estimator for [company's name] major projects and highways. My background... I've come from a quantity surveying background. So, I started off as a training quantity surveyor. Oh, about 15 years ago now. And I worked my way from being quantity surveyor right away through to being a managing quantity surveyor. And then I took a bit of a curveball and went into estimating because that was probably where my specialisms like. So, prior to joining the estimating team, I'd done a lot of negotiations on Highways England projects. So, I'd done various smart motorway, negotiations, pricing, supporting the estimators and that sort of thing. So, and then since then, yeah, various various types of highways projects, HST projects. I'm currently working at [project's name] looking at the [project's name] construction and the old highways project the here and now. So, yeah, so it was still in that sort of build, but probably a bit more diverse.

**Interviewer:** Do you plan to be an estimator or it just, you know, be like this?

**Interviewee:** I've always enjoyed the pre-construction side of construction... enjoy and I suppose I was good at the pre-construction side. So, that's where it took me in that direction. So, that's how I ended up becoming part of the estimating team.

**Interviewer:** So, what you're doing now, is it, you know relevant to what you learned from school?

**Interviewee:** Yeah. There are still things that there's things there. The basic principles of measuring, your basic contract law, all of the technical side of things, but it's probably taking what you've learned at university, college, etc, and then taking it further in saying, getting that practical experience like I worked on various jobs to becoming an estimator and had that opportunity to understand how construction methods work in principle and then took it back to first principles when pricing something and saying, well, hang on, how fast is it going to take me to dig that hole?

**Interviewer:** Would you mind to take one risk, you may, I'd say mostly happened and then talk about all the things you will think when you make the assessment on it, the probability of occurrence of that risk and its potential impact on the project? When you assess this, what things do you think?

**Interviewee:** Okay. So let me think about this one. So, if I put it to a recent way of looking at projects at the moment where we were pricing things up, and one of these is a highways project. So, we're looking at, first of all who holds, so who holds the risk under the contract? So that's the first thing. So yes, you're assessing the risk, but you're trying to understand who holds that risk from a tendering point of view. Yeah. So, what you don't want to do is go and price yourself out of the water by pricing risks, it's held by the client or the employer or whoever... whoever's the named person under that side of things. So, it's understanding that ownership of risk in the first basis... is a, it's a key part. Once you understand that, and you will have a feel where you'll say ' I'm not sure if that makes sense. I'm not sure if that sits with one person or another person'. You then... so that, that you put part to one side, but at the same point in time, there'll be some domestic risks that you, you look to assess. So, If I looked at ... there's probably a certain number of risks that you... generic risks that you go over and over and over again on a NEC type contract or a highway... highways, or an NEC type contract. So, you'd be looking at assessing what you, well, basically taking your knowledge and understanding that now. I always like to price it, prior to assessing my risks, if that makes sense, because then so I can identify the risk is on pricing. So, as I'm pricing, I'll be identifying risks. So is there an over measure and under measure, is there a risk that's going to get larger, smaller... getting take longer etc.

So, I would go through, I would collate my risks and then what I would do is, is then filter back. And then you probably enter into some sort of peer review, if that makes sense, where you talk it through and you talk with others, who've been doing probably a similar thing. Maybe the methods engineers, maybe the planners, maybe the construction, any sort of technical input you getting along the way to see whether you're getting similarity. Because you generally get similarities on risks because if you… you're thinking of a risk, somebody else's generally thinking of that same risk. Yeah. So, once you've got that consensus, I would say, then you're looking at how you evaluate it,

And depending on what the risk is. You might be able to say actually... let's think of a couple of examples. It might be a time impact risk. So, you might say actually it's gonna take me two weeks longer to do this. So, you make that, you talk it through, and you decide how probable is that to happen, if that makes sense. So, if you, depending on what client and what...why you look at it, you may have a situation where you say actually the percentage, or the probability is too high to sit in a risk register. So, you may say actually I'm gonna build an allowance into my base price. Yeah. So, you might say i.e... So, it might be a 75% and higher probability. I'm gonna put it into my base price. So, you put that to one side that becomes part of your base price.

Anything with that lower probability, you then assess, and you basically tried to put it into a three-point estimate is how I would do it from a risk point of view. And I would assess and say, depending on what the risk is and there will be discrete risks that are cost related. So, they would fall into QCRA (Quantitative Cost Risk Analysis) side of things. And then there would be some that are schedule related, which would fit into QSRA (Quantitative Schedule Risk Analysis) side of things. There's always an overlap. Don't get me wrong. There's always an overlap. So, there's that assessment there.

If you then take some things further and you take it probably a little bit outside of the highway's environment and maybe more in systemic like nuclear or the energy side of things. You're also then beginning to look at maturity of... maturity of design... maturity of scope-definition type risks. And that's where you might end up putting some form of uncertainty on top of any calculated risks allowances you make if that makes sense. But generally, in the highways side of things, even if you take it on a design and build project, unless it's very innovative or that sort of thing and generally under 50 million, it's probably going to be quite straightforward, less complex, and that sort of thing. You'd probably focus more on what you know and there's a good, you've got a good level of experience to be able to assess those risks rather than necessarily having to put things in like definition models, estimating uncertainty and that sort of thing. If that makes sense. Yeah.

**Interviewer:** So, for example do you worry about maybe the inflation risk and how will you, I mean price it for the risk allowance for it?

**Interviewee:** Okay. So, the first one on that one is I would check if I hold the inflation risk, if that makes sense. So, if I do hold the inflation risk, which can be interesting. I'm then looking at how I think... so, it's pricing it then understanding the factors. So, breaking it down into bite sized chunks though. One of it's going to be my labor costs. So, how much labor have I got in the job? So, what's my key material, key criteria, key resources that are going to drive my inflation. And then I would take I probably take some guidance from my wider procurement teams and things like that. But at the same point in time, there are published guidance as well to look at and also just a general feel for what you're getting back.

We get supply chain quotes in all the time, and you get a feel for how much things cost. And if you start to see prices escalating, which they have done in the past, you begin to look at it and say, hang on, I'm really worried about that area. So, you might actually say right, I'm going to put an inflation calculation into my price, but I may also identify and the risk of an extraordinary inflation effectively. So, and even if I know that the contract says to me, we're going to use a certain index, we may find that there are some risks. We need to make some differential inflationary allowances within our pricing, and we've got contracts, or I've done contracts where we need to use the differential inflation because we recognize that the published inflation is too low for infer labor, for example.

Yeah. So, there are ways of means of assessing it and you just, you've got to work with the mechanisms that you've got and the the knowledge you gain.

**Interviewer:** So, for example, for the inflation, how do you think of the controllability of inflation? And if I mean this risk becomes to be the opposite state? How do you think that you can control it or not?

**Interviewee:** Can we control it? We can make an allowance for what we know at the time. And we can make a risk assessment. So, we would, generally, even if we were taking a fixed price, we would make an allowance for inflation. Yeah. But there would be... there would then be a risk generally in our risk register or something like that. And depending on the gauge of what the project team views or whether ... and even to a director level, wherever they feel that risk... extraordinary inflation is a high or low probability, if that makes sense.

**Interviewer:** Yeah. So, for your personal perception, how do you think of the controllability of the risk? And if its controllability changed, how do you think it will impact your judgment?

**Interviewee:** Emmmmm........ Good question. I think it is... is it controllable? I think it can be controllable. And there, definitely... there's an opportunity with it. If that makes sense. As a business model, there is also areas where it's risk to us and generally where we see it as an opportunity, we would take the inflationary risk. Yeah. So, we would look to basically make potentially additional margin and that sort of thing and on that side of things. But where we feel it's, it's too much of a risk. We would look to pass it back to an… to the employer or the client effectively to manage using their own mechanisms of how they generate inflation, if that makes sense.

**Interviewer:** Yeah. Yes. I know its things to be what your company will do. So, I mean, for yourself, are you willing to take more risks or maybe you are more risk averse?

**Interviewee:** It depends on, it depends on what I'm pricing, if that makes sense. It depends on if I'm pricing a stuff I know. So, using the highways example, I would be less concerned with take it. We've not taken the inflation as risk if that makes sense. I would be happy to take the inflationary risk on a highways project because I've got a library of information and various historic information that I can sort of fall back to and a good handle of what the market is saying. If I'm going into an area of a new market, I'd be a little bit more concerned. I'd be at big far more risk adverse. Yeah.

**Interviewer:** So, how do you think your attitude to risk, and do you think it can affect your judgment to some extent?

**Interviewee:**  Do I think it can affect your judgment... I think it does affect judgement. I think it's something that grows. It's something that matures in individuals. You have to experience dealing with projects where risks have impacted significantly or the vice versa, where risks haven't impacted as severely. You need to be able to see that... that to be able to gauge your view. I think it's a key part of how we do business. To be honest, how I would do businesses is if somebody comes to me with a tender about a risk allowance and that sort of thing, I'm very, very surprised.

**Interviewer:** Yeah. Yeah. So, you know, I think you're working a team, right? So, do you always have I mean, make the same or similar judgment of risk with other estimators or maybe sometimes you will have different judgment?

**Interviewee:** I will say everybody has a different judgment on the same risk. What you need to do is come to consensus if that makes sense, because we all have differing differing experiences. Yeah. So, you find that some are more risk adverse, some are more risk... more, more cutthroat, if that makes sense. So, you need to get to the happy medium and recognize what the situation. That's where I would say ... risks cannot be done on it. So, a risk has to be done as a consensus because it's a lot of different people's opinions of the impact of risk. You will probably find that everybody agrees that that is the risk, but everybody will have a view of the impact and you need to come to that consensus of what it is you can't purely.... you can do it in isolation, but more, many minds make good answers. Yeah.

**Interviewer:** So, to your understanding, what makes you make different judgement on risks? You just mentioned experience and any other reasons?

**Interviewee:**  I think experience. I think uniqueness. So, is something repetitive and repeatable and that sort of thing you will make different judgements on or is it a one-off if that makes sense. Yeah. Or just think about any other factors that might ...so, experience would be one... contractual terms to a certain degree may influence how I think about risk, if that makes sense. If I'm more culpable for that risk, I may think of it in a hell of a lot more detailed than if I'm not necessarily culpable for that risk, if that makes sense.

**Interviewer:** Yeah. You just mentioned you were finally making a consensus. So how do you know other estimators, whether they are right or wrong, whether they do a good job, how you know that?

**Interviewee:** Well, that's a good question. I would say...emm... right or wrong... I don't think there's ever a right or a wrong answer if that makes sense. It's very much a basis of understanding. So, firstly... so, if you go to the theory side of it, the theory side would say you seek feedback, you understand where you made gains and losses on previous tenders. So, a good estimator will go back and seek feedback from the sites, the tenders that he's been successful on effectively. And from that you'll gauge a view of of how successful someone is, if that makes sense. Yeah. But from a... from seeking consensus. I think it's actually becoming more of a debate, and you've got to trust each other, if that makes sense, to… Generally, you'll be, if you... if you are... you'll be... it's very rare for people not to agree on what the risk is. Yeah. The impact of it will be subjective and you just have to sometimes get to the point where you agree to disagree if that makes sense. Yeah.

**Interviewer:** Yeah. So, I think you have experience in many highway projects and pricing the risk allowance for risks. So, have you ever encountered a risk which you take, and you feel it's difficult for you to, I mean, quantify the allowance for it because maybe the context of the project is you're not familiar with it or it's complex, or maybe the design... there are some technical challenges? Yeah. So, can you take one as an example and tell me how you finally approach it, or you finally decide the allowance for it?

**Interviewee:**  Okay. I'll… I use an example. We had to take the weather risk on a project. Yeah. So, the weather ... so, bad adverse weather basically. Now usually on a contract you would under the NEC, you'd be protected by the 1-in-10-year calculation effectively. So, what we had to do was basically we had to come up with our own calculations for that... for all the weather, all the weather was our risk effectively. So, what we did was we actually took the historic data for the area. Yeah. So, we, we took it all... we took all the information. We analysed how many times that would... it would impact effectively and differing things that would basically mean that we can actually go to work. And we assessed the amount of downtime days that we expected onto a project. Then we modeled it a couple of times. So, we threw it through several models because, of course this data is subjective. Yeah, yeah, yeah. So, you run it, run it numerous times and things like that. Yeah. And then we basically made an assessment and said, this is what a minimum allowance, this is most likely, and this is a maximum allowance and that's generally how well, we we've sort of taken those sorts of complex risks.

It's to be able to put it into... it's you, you need to be able to quantify something, if that makes sense and make some assessment of what a risk. So, you need to understand what is the impact of the risk? You might be wrong. Don't get me wrong. You might be wrong. What the impact of a risk is. And you'll find out later on when you go into delivery, that that the impact might be wrong, but at least if you've got a basis for what you've assessed, you then provide yourself something as almost a negotiation stage to engage with somebody else to peer review it, check it... confidence check it. Go through and negotiate with a client if that makes sense and explain why you've made those allowances. That's how you sort of get to that point if that makes sense. Yeah.

**Interviewer:** You just mentioned that you were thinking about the impact of that risk. So, in specific, what do you mean... what impact do you mean? It's the financial ones or?

**Interviewee:** Mainly, I would suggest tendering stage it's the financial and the time side of things. Yeah. At the same point in time, you don't want to take on a qualitative risk. So, something like a reputation or an environmental risk that you know is gonna cause significant problems to the business, or you highlight those, that tender as part of that review at governance to say, yeah, we've got 10, or top 10 risks. Two of these are reputational risks. They have no financial bearing. We can't understand what the financial impact of this. This is you get work, or you don't get work sort of thing.

Yeah. Well, you go. You've got real sort of reputational concerns on those ones. They would be assessed as a qualitative risk, which you would generally score to give you an impact, if that makes sense. Yeah. And you would use a five-by-five sort of chart effectively to assess what that impact looks like and the severity of that impact.

Whereas if you looked at some of the financial ones, yes, you try to categorize them to start with. And that's usually what I would do very quickly with a risk register when I first populated it, come up with a very quick qualitative score of what's the impact against the severity and get you scoring in that side of things. Because generally those ones that you're working, your 18, 20 row, where the top 10 risks are going to be 80% of the allowance that you need to build in, basically. So, yeah, depending on how fast and how much time you've got to work on it.

**Interviewer:** So, when you do... I mean fill in the register, are there any principles or rules you have to follow, you have to comply with?

**Interviewee:** So, any rules... I think you... you always very clear on that... there's generally a principle of how you complete a risk register. If that makes sense, there's usually a standard... that's either provided by the client or you've generally... I've always seen you're describing your risk, you're describing the impact, you're describing a cause, you're describing the scoring, you most minimum, most likely maximum probability side of things. What is it? Is it a one occasion or is it how would you, how are you going to model that score? I'm trying to think to answer your question to be honest.

**Interviewer:**  Sorry. I mean that when you quantify and price the risk allowance for the risk, are there any rules or principles you have to follow or comply with?

**Interviewee:** I wouldn't say there is, to be fair. It's based mainly on... mainly on perception, but I said the big role for me would be to seek that consensus. If you... you will complete a risk register and you will score it and you will run your first couple of runs on it. And the first couple of runs, when you run, you get your tornado charts, something like that. Risks will appear not as the group, see that they should be. And then you get that opportunity to consult, and review and get it to the point where I actually feel comfortable that actually those other top risks and these are the risks that you're assessing.

**Interviewer:**  So, I think you just mentioned that you will reflect on the project or the work you do. So, have you had any rules of thumb or guiding principles made from your previous experience? I mean in the risk assessment.

**Interviewee:** So, what guiding principles from what I've done in the past influencing how I assess my risks is that... that way you are? I would say. Yes, they dominate how I go about pricing risks if that makes sense if I'm doing repetitive work. If I'm not doing as much repetitive work, they're there. But you're more seeking to agree wherever the same risk exists if that makes sense. You're seeking to understand from others, wherever you think that there is that same risk. Whereas if you're doing that repetitive junction improvements or something like that, you look at the detail, you look at the information, does that same risk apply on this job as it did on the other job? Ground conditions on one job could be extreme to another. if that makes sense. See, you're looking to find those key things and there'll be generics that happen over and over and over again, whatever type of job you're doing, but there will be certain influences as well.

**Interviewer:** Yeah. So how do you think the idea that one day maybe, we do not need estimators, maybe there are some algorithms or software, computer softwares, they can automatically estimate the risk allowance?

**Interviewee:**  I think there'll be estimators for a long time yet because I think the human factors are significant in the type of work we do.

Maybe in some of the more simplistic elements, yes. I'd agree that you could start to come up with so many algorithms. On highly complex civil engineering projects, I think you still need that human... that risk adverse human. So, understands what's going on. I think on simplistic things or more repeat, doing the same things in exactly the same environment, I think it could happen. Yeah. I agree, but I think on some of the more complex stuff, you still need that human, human calculation.

And taking it back to first principles, you know, that's the key thing of how do you build something? Well, actually you build something with people. You'll be able to meet with plan. You build something with materials, and you build it up in your head and we've in engagement with specialists to understand how you do it.

**Interviewer:** Yeah. So, you know, what's the advantages, I mean, of the human being to do this work?

**Interviewee:** What's the advantages? Experience. They've been there, done that. I've got a t-shirt to a certain degree if that makes sense.

I think the advantage is to be able to be versatile. I think sometimes, sometimes computer algorithms and things like that take things as given. So, you find yourself in a tender situation where things are constantly evolving to queries being asked by the bid team and people are wanting to change their mind on things, whereas algorithms aren't very good. Generally, when you, when you say to it, actually, I don't want that. I want this if that makes sense. So, we... and clients are the same to a certain degree is.... I might turn round and say, actually, yeah, I want that bridge, but I never want it painted green. I want it painted pink. Yeah. And the algorithm might not understand how to paint it pink because pink might be a much more expensive color than to paint something, then green, if that makes sense.
