# Supplementary material for: Critical factors influencing cost estimators’ judgements on cost contingencies in highway construction projects: An empirical study in the UK
Source: PLoS One. 2024 Dec 16;19(12):e0314665. doi: 10.1371/journal.pone.0314665 (PMC11649144; doi:10.1371/journal.pone.0314665)
Supplement: S2 File — (ZIP) [file pone.0314665.s002.zip › Transcription (Interview E).docx]

**Interview E-Meeting Recording**

**Interviewee:** Yeah. Thank you. And, so, would you mind to firstly have an introduction of yourself, like how you become an estimator? I'm quite interested in about this.

**Interviewee:** Yeah, certainly. I'll quickly sort of tell you about my sort of progression of my career. I studied civil engineering at Nottingham University back in 19... I think I graduated in ... 1989, back in the last century. I... after university, I initially went to work as a sort of a site engineer for a civil engineering contractor, working on highways projects and many other kinds of projects ... sort of... all sorts of infrastructure projects, really. I did that for about seven years.

And during that time, I did my institution of civil engineers... sort of charter... chartership. So, I became a member of institution of civil engineers. And then, I kind of... I got to the point where I could either carry on in sort of the contracting and go more into the project management side of things. But I was also quite interested in engineering and temporary works and that side of things. So, I actually joined a company called [company's name] that there were a lot of temporary works and formwork and shoring and all the sort of equipment to build structures. So, I worked with them for about 20 years mainly on overseas projects. I actually, I worked in South Korea for a while and places like Indonesia and a little bit in Australia. So, I sort of had a... when I was a younger man, a had a bit of a wanderlust wanted to go to places.

And then I spent quite a lot of time based in the UK but going as part of the export business going to see people and working on projects, in other parts of Europe and in the middle east. So, I did that about 20 years until we have a look. I think that was until 2016. I got to the point then, well I was getting a bit... it was... I was finding the work… it was a bit... it was a bit sort of full on. There was... it was seemed to be 24/ 7. You couldn't switch off from it. And I was getting to sort of getting to my late forties and I felt like I was... it was all work and no fun, no life. So, I looked... I was looking for other things that I could sort of turn my skills to and [company A] was part of a bigger group called [company B] and they had a construction company, and they were looking for people to train as estimators, cuz they were that side, I think. So, I joined [company B] 2016 as an estimator and many of the... obviously I had a fairly good knowledge of the construction process from what had done in the past. And cuz I spent quite a lot of time as an engineer, I was fairly meticulous and quite good at breaking things down and that side of things.

So that's when I really started my estimating career. So that was sort of 2016. And in [company B], we were working on sort of highway projects and some water infrastructure projects. Really since then, until now, I've sort of gradually gained more experience on projects. A lot of… it's sort of really learning as you go along from your colleagues and other people when, and sort of feeling your way. I have a say, I have hoped around a little bit. I worked for [company B] for about two years and worked for... then I moved to a company called [company C], which are another civil engineering, a smaller civil engineering company who I knew [person's name] from [person's name] was my manager there.

And then they were getting a bit quiet. And I got to got tempted to go work for [company D] in the highway section where I work now. So, it's ... sometimes the work's a little bit tedious, but what I do like is that you can... you get a project, you can work your way through it in a logical process. And you've normally got a reasonable amount of time. And what I used to find with, when I used to work at [company A], I go to work and I'd have my own projects to work on, but it was also helping other people and managing part of the team. So, I used to find that I go to work and my day would totally change within about half an hour. So, I worked there and then I would get to about four o'clock and then I'd start working back on my own projects again. So, I might... so working as the estimator, I find that I've got a better. Still work fairly hard, but I've got a better balance in my life now. So that, yeah, that's me where I'm really.

**Interviewee:** Oh, that's an interesting experience. Yeah. And so, how do you think your, you know, your former experience, how they help with your, you know, the estimating work you're doing now?

**Interviewee:**  Yeah, I actually... I actually think that the experience I had when I very first, when I graduated, I actually worked on site used to do the sort of the setting out, to decide where, you know, the position of things and where things went and liaising with everybody. That knowledge of the construction process I think in those early, sort of those first seven years has helped me an awful lot because I could visualize a project of what needs to be done and the processes. So that when... obviously when you're estimating, you're breaking things down into their sort of constituent elements and then pricing those up. So, I think that side of things has helped a lot.

And then I think sort of the engineering side that I was doing with [company A], That's... I think it's that attention to detail has helped sort of things. I'm fairly good at visualizing what's required and barely feel unfairly sort of good at making each of... I've sort of ticked all the boxes and got the detail.

**Interviewee:** Yeah. Thank you. Thank you for sharing about this. So, in your... I know you have participated in many highway projects, and so in contractor's risks, I mean, risks which are undertaken by you, could you pick one risk which mostly happened and talk about, you know, all the things you will thinking about, when you make... assessing risks, their probability of occurrence and its impact? What things you will think about?

**Interviewee:** So, one risk that we normally up... one risk that we normally have to consider when we're pricing a project. Let me think really, obviously.... one, let's think of a good one. So, I'm just thinking of a good one.

There's always one of the risks. One thing that always crops up a lot is... the sort of, I find it's the work at the interface with other operations that are going on. Like... for instance, the companies that do all the diversions of the undergrounds, like the water, the stats as we call it, the electricity, the water pipes, the telephone cables. We always find that that's quite a big issue in terms of... They... they're working... they're normally working directly... they're normally paid directly by the same clients as we are, but we have to coordinate them and manage them and do our work around them. So, that's always an issue because we are expected as part of our contract to make allowances for them, but they can... There're a bit of what I would call a law to themselves. They are a bit... they don't really care about us, but we have to care about them. So, we have to... so that can cause a lot of... can cause delays to our program, which obviously time then you can get into to liquidated damages, if you overrun a project. So, you've, and also it can cause a disruption to our working patterns. So, really with the stats or that the services, we obviously... our planners are always trying to very accurately... try and plan when their work will be and how we work around them. It is always ...there's always some uncertainty, whether they will perform as we think they will. Obviously, sometimes, some of that risk can go back to the client, but the sort of the general disruption, we often have to add some additional time for that side of things, in terms of our risk and things, the risk of things over running and additional preliminaries costs for our staff for that period.

**Interviewee:** So, do you maybe have a risk register to...

**Interviewee:** Yeah. We have. What we tend to do is we have... we always have... we have a risk and an opportunity register, cuz there's risks, but there's also opportunities. Obviously. There’re the two sides of it. But yeah, we have... we normally have a risk register where we go through. Often, we'll work as a team. We'll perhaps have a workshop for a few hours, and we'll all sit and throw sort of brainstorm, what risks we think that are. What I tend to do is, when I start a project and read through the information, from that point, there on, I'm making notes about things that I need to do and things that might be a problem, things that we maybe need to ask questions about.

And then, there're some of those items then get... put onto the risk register. And yeah, we often will sort of subsidize. We'll have the whole bid team involved in putting together a risk register. So, everybody's putting in their view of things. And then, we'll... probably myself, or maybe the commercial manager will then take that register and look through and decide which of those risks are our own buyers, sort of thing, which risks are our sort of problem, because a lot of the risks are actually, we can pass some of those onto our sub-contractors or we can, or some of the risks that actually the client's risks. And then, and some of the risks we can mitigate, and they disappear. And some of the risks, we might try to put some assumptions in, if we're committed to then ...to sort of eliminate those risks.

So, we'll kind of go through all the risks and then try.... and try to sort of move away... you know, try to cancel out quite a few of them because there are other people's or not a problem. And then we'll look to sort of... we'll look to then try to put some costs against those risks. The ones that we think are ones that we need to take into account.

**Interviewee:** So, for risks, which you just mentioned, you will allocate cost to some of them. So, can you take one of them as an example and tell... and talk about, you know, what things you will think about when you price it, you know, how you allocate the price for it?

**Interviewee:** Yeah. Yeah. Really. Take for instance, would say delays due to the stats, the service people not doing their work properly. Obviously, those delays were obviously there's delays to the project. So, you've got the... so, one thing you've got is the chance that you start to have to pay liquidated damages as a penalty for overrunning. So, those costs are quite easy, so we could say, well, we think we might be delayed by three weeks. We know what the liquidated damages are per week, so that's quite an easy cost. And then, so we've got it... we've got a cost there and then we'll say, well, so, you say we've got, I don't know... say we've got a hundred thousand pounds as the delay damage and then we'll say, well how likely is that to occur?

**Interviewee:** So, how you decide the likelihood? You know, what things you will think about?

**Interviewee:** I think you look at ... yeah.... I think you look back on sort of previous experiences. The current, obviously at the moment, because of the sort of the pandemic and things, everything's a lot more disrupted. So, you've kind of... this sort of previous experience, plus how things are at the moment in terms of, you know, the current state of things, because what we're finding at the moment is skirt off on a bit of a tangent, but material it's quite hard to get materials now in the UK, there's a big delays and because of all the disruption. And I think also because there's not enough people trained to drive the lorries that deliver the materials. So, there's sort of, you look back at previous history of whether this has been a problem in the past. There's often somebody is as we would say, had their fingers burned. They've had a bad experience in the past so that when they start to look at another project, they.... these things are very much at the front of their minds. So, they'd be... they become... they may be over... they may be sort of over, maybe put too much risk into that because they've been troubled in the past by it.

There's a bit of an emotional side of things. Sometimes, you know, you've had a bad experience with it. 'Well, I'm not going to let that one catch me' next time sort of thing. So yeah, so, you look at the sort of, you know, the risk of it happening based on what you've experienced in the past. And sometimes, it's often, it's very, quite hard to actually... You know, you've just got to make a bit of a gut feeling, sort of thing, a judgment of what you think.

**Interviewee:** Yes. Okay. So, for like the risks, which you just mentioned, how do you think the controllability of it? I mean ...the controllability, I mean whether you can do something to manage it, control it. So how do you think the controllability of that risk and you know, if the controllability change, do you think it will affect your assessment on it?

**Interviewee:** Yeah, like you say, you certainly could look at... I say, take for instance, like say, the risk of us being delayed and paying damages, can obviously look with the planner rates. How much... as sort of risk allowance, time risk allowance, the original program, whether this ways to risk. If that becomes a problem, whether there's ways to reschedule the work to get around the problem, cuz sometimes you can have one problem, but if by doing other things, by changing the sequence or working in a different pattern, you could... you can remove or reduce that problem.

Sometimes things are very, very critical. When it's a particularly critical part of the project and in those cases, then there's obviously more... you'd probably put more likelihood on that particular risk occurring. So... yeah, certainly sort of look at each risk and then we'll look at that particular risk and then try to sort of find... certainly tries to find ways to mitigate it as best we can. Cuz you kind of... you could... if you're too pessimistic about everything, then you'll never win any projects. You've got to take some chance. Some things are a real sort of major problem, that's sort of a showstopper, but other things there's often other ways around the problem, sort of thing.

**Interviewee:** So, you know, for yourself, do you think you are a more pessimistic or optimistic person? And how do you think, whether, you know, your personality will affect your judgment?

**Interviewee:** Yeah, I think I would say I'm a bit of a pessimistic person to be honest. I think it's the color of the engineering type, you know. You realize how hard it is to sort of make things actually happen. But I also, I think, cuz I know I'm pessimistic though, I often... I'll kind of, your kind of ... you might go through the risks and decide what it's this much, but then you may actually sort of recalibrate it cuz you know, you're pessimistic and you know that if you're too pessimistic, you won't... you have too much risk pot and you... you may not secure the project.

So, cuz I think, cuz I know I'm pessimistic, I kind of then recalibrate it a bit to sort of say, well, I know I'm too pessimistic, let's reduce the risk or sort of, we're also very... cuz obviously we often do these as a group thing. So, my pessimism can be balanced out by other members of the team that are more optimistic. So, when we actually look at... cuz we often sit and say, well, okay, we think if we have this problem, it will cost this much money. And then we'll say, well, how likely is that to happen and how... what's the sort of impact? So, you end up with a bit of a score and we tend to do that scoring as a group. So, my pessimistic can often be balanced by somebody else's more optimistic approach because often if somebody's, you know, somebody's obviously.... we often have some people who are more sort of working on live projects, involved with the bids, so that... cuz when you, I think as you get older as well and you're a bit away from the sort of the actual construction process. You're a bit more pessimistic, but sometimes they'll say, well, no, I don't think that would be a problem. We can get around that. So, it kind of, my everything balances a bit more. So, we get a more sensible level of kind of risk.

**Interviewee:** Okay. So, it seems that you know, people with different characters, they will have different judgment on the same risk. So, except characteristics, any other reasons you think, you know, make them make different judgment on the same risk?

**Interviewee:**  Yeah, I think also there's that, as I said before, that sort of previous experience, or sometimes if you've priced, maybe you've priced a couple of projects in the past and maybe you're miss... you've not secured those projects and you've been too expensive. You kind of... you start to... it's like we say, we have to sharpen our pencil sort of thing. We have to try harder. So, I'll think as even though you may say you've worked.... bid two projects and you've not got the last two, when it comes to this one, there's... there may be more pressure. You feel more pressure to be... you know, to get this one. So, you may be a little... so there's probably like that sort of pressure of winning work that kind of maybe distorts your thinking a little bit.

And also... just think what other things affect it early. I think it also... you sort of.... also, you kind of, I think if the group's quite positive, the team that you're working with are all quite positive and if the feel that... I think that have... sort of you feel that people will be able to overcome problems. I think that can kind of give you more confidence that yeah....

**Interviewee:** Oh, you mean the team atmosphere?

**Interviewee:** Yeah. That kid of... what's the word. You know, sometimes you get that sort of, it's a bit of synergy, isn't it? Really where.... it's not really synergy, but it's kind of, you know, the team, the team sort of rapport on everything, and you feel... you feel more positive about it. So, I think... you think, oh, well, we can overcome this. We can do this. We can do that sort of thing.

**Interviewee:** Ok. So, you just mention that people in a team, they will have... sometimes have different opinions, different assessment. So how do you know, you know, whose assessment is right, or I will say... much better? How you judge that?

**Interviewee:** Yeah. That's the thing we were we will talk about in the daily record. I think we naturally tend to sort of differ... you know, we have a deference to people that are more senior in terms of their role and everything. It's strange now because I'm kind of in my mid fifties now, age wise, but some of the people that are more senior in role, in that... you know, are actually younger than me. So, it's kind of... but I tend to think, well, even... you know, it's less about an age thing and I think it's more... I tend to think, well, you know, they're at a higher level, so, I need to respect what they say sort of thing. But I think... I was thinking, we were talking about as though... I was thinking... we were saying we're really, you know, we should even... we should be challenging some of their decisions more because we shouldn't just accept them.

You know, we tend to think... as you get older, you get a bit more pessimistic perhaps. Sometimes, I'll say, 'oh, well that one will be a problem. This is one of the other things we find.' You'll get ... you will go to sort of meetings as you looking at these things, we'll raise an issue that we think is a problem and now we all know we'll go overcome that. We can do this; we could do that. And you're thinking... will you be able to... I think you kind of... you also get a little bit... you feel you've got to protect yourself personally, so that further, if you get the project and then there's a problem. So, you kind of... as long as you feel you can protect yourself and make sure these decisions are written down, then you're more happy to accept that somebody senior said, 'well, no, we will overcome that problem' sort of thing. That won't be a problem. I'm rambling, rambling on a bit.

**Interviewee:** Okay. Yeah. So, in your experience in highway projects, have you ever been. encountered a risk and you feel it's difficult to quantify the risk allowance for it and how you finally approach it? I mean, maybe sometimes the context is changeable or, you know, it's complex, or you're not familiar with that or sometimes there are some design challenges or something like that.

**Interviewee:** Yeah. I don't think I've ever... I think we've always been able to quantify within... I think you get to the point, if a risk is so difficult to quantify, then you think, well, should we... how should we be taking? You know, should we be actually taking on board this risk and should we be bidding for this project?

So, now, we had... we got to the project recently, which we started to work on. Then you... we were building... it was building a highway over some very soft ground. And you had to search, you have to build the embankment higher and then let it settle down and so that the ground compressed underneath. And the risk was so ...and all these risks were put on the contractor and the risks are so difficult to calculate, that we actually... and the client wouldn't accept any responsibility. So, we sort of... we got to the point where we actually, if we can't quantify the risks, then really, it's too... too risky to actually put a tender in.

I think most things you can actually come up with a reasonable assessment. And I think if you've got a project with lots of risks, then you ganna make... obviously you've got ... some of you will overestimate, some you'll underestimate. But hopefully, hopefully the pluses and minuses will balance. But then let's say if you get these risks that are so so difficult to quantify, especially if you think there are large risks. Then, we tend to get to the point where we think, well, should we kind of politely back out of this one?

**Interviewee:** Okay. So, you know, from your experience in calculate risk allowances and assessing risks, have you made any, you know, rules of thumb or principles for yourself from your previous experience?

**Interviewee:** Let me think really. Obviously, calculating the risk, normally you could... you can come to a sum of money, which you think is... you know, that would be what it would cost, if this risk occurred. That's relatively easy. It could... might be a time thing. And we know what... you know... we know what penalties there are, we know what it costs to run the project. So, the time thing's not so bad... I think it mainly comes down to... it's that what, you know, how much... what's the likelihood of that risk happening, which is the hard one to quantify, you know. And I think that a lot of it's just that previous... your previous experiences and what the group things really.

Yeah, I think a lot of these things become a group decision really for ourselves anyway. Where you maybe have 10 people in putting into it, sort of thing. So, between us and often there'll be some people that are more site... you know, actually site-based who are.... cuz, you tend to be... as you're an estimator, you tend to obviously be away from the... you tend to be working more remotely away from real projects that you... I think you maybe lose your reality check sort of thing a little bit. There're sometimes... there's people that are actually still, you know, actually at the sharp end, as we would say, actually doing the work that give you that reality check that, you know, 'No, that's not a problem or yes'. You know, that is a risk sort of thing.

So, I think for me, it's very much, you know, listening to... have your own opinion, but listening to other people and come into a collective decision on it.

**Interviewee:** Okay. Yeah. Thank you. So, you know, do you use any software to help you assessing risks, calculate risk allowances?

**Interviewee:** No. Not really. We use what we tend to do for assessing risk. I can't remember what the software's called, but cuz we're all working, we tend to work remotely anyway, but we have one of these... can't remember... I think is part of the [software], where we can all... you can all input into it, and you get like a spider diagram of all different... Somebody sets some basic thing. I forget what it's called now. I was trying to remember it this morning. So, we tend to say, when we have like a risk workshop, we tend to use some sort of collaboration. It's like a collaboration software, isn't it? Where you can all input. It's a bit like ...it's not the computer equivalent, there everybody having a bit of sticky paper and putting it on cuz that's what we would do in the old days. Everybody would have a sticky bit of paper and write them on. But, so, the only software we tend to use after that is just an Excel spreadsheet. That will ... from the sort of the workshop, we'll collate all the risks and then just use an Excel spreadsheet to... obviously, actually, the actual costing of actually working out the value of the risks. We just tend to use some estimate... just normal estimating software. We use a package called [package's name] at the moment, which I think is quite... but that's just... that just works out, you know, helps you to build up the cost of things.

**Interviewee:** Sorry. Can I ask how does that software work? I mean, how it calculates?

**Interviewee:**  All that does is calculate the sort of the value of a, you know, that it's really, that's just the estimating software. So, really, it's got a database of rates for different, you know, elements, sort of materials and people and plant and machinery. And then you can build up the cost for a particular item of work from that. So, all that does is works out the cost, the potential cost of that and then it's taking those costs and putting them into this spreadsheet and then deciding how much of those costs. You're going to take us a risk allowance?

**Interviewee:**  So, you mean for every project you will have to fill in something?

**Interviewee:** Yeah. For every project, we'll have a risk register on an Excel spreadsheet and where we'll put in... we'll go through the risks. We'll list them all out. Some of them will make comments that they're the client's risk or that we'll pass that... Some risks you can pass on to subcontractors. And then the risks that we think we've got to consider, we'll then cost... we'll then work out some estimates and costs for those. And then, those costs... and then we'll look at the likelihoods of those occurring. So, you end up with a... you build it up a total sort of what we call a risk pot. So that's the money that you would have as a risk then. That's what we would... that's what we would then put that into all sorts of tender settlement meetings with the, you know, with the directors and then they would have the ultimate decision. You know, we'd say this is what we think. And then they would have the ultimate decision whether they increase that or keep it or reduced it down.

**Interviewee:** So, because you know, risk allowances, it seems like a financial concept. So, when you think about the impact of the risks, will you only taking the financial impact into consideration, or, you know, you were thinking about other things.

**Interviewee:** We would ... mainly... that mainly the financial impact of that risk, but obviously, yeah, there are, you know, there's obviously possible risks to reputation and I suppose winning future work with a client and things like that. But for us, really, we were more concerned with the financial aspects. Obviously, the risks may... maybe a time related risk, but obviously we would then convert that time into what the costs would be for that time. But yeah, for us really, I would say it's primarily the financial side of things.

I think maybe at a higher level, they may take an overview about, you know, the clients, and whether they want to... Because sometimes you keen to work with a client, so you can secure a lot of work in the future and you... some people may have worked with the clients in the past and feel that they are fairly reasonable people. And, you know, you've got opportunities to to talk to them and you know, there'll be reasonable and not to be too... you know, I don't necessarily... would be understanding if there's problems. So, I think there's that side of things, but I think really... I think that that sort of aspect more that... the more senior directors get that sort of feeling more than ourselves. And I think that's when sort of the final, sort of settlement meetings, when we agree, what price are we going to put in. I think that sort of subconscious, their sort of feel for things gets brought into the equation really, in terms of... that how much risks we actually decide to put into the project.

**Interviewee:** So, for you, do you agree with the process? I mean, do you think you should only think about the financial... from the financial perspective, I mean?

**Interviewee:** Yeah. I... Yes. I think, yeah, I think so, because I think as estimators, we kind of feel that our role is to give the... sort of the wider team what we think is... what we think the project will cost to construct, and also tell them what risk we think they should allow for financially. So, I think that's what we feel... what then get decided is up to the rest of the team, then really. But I think what we feel is that what we've done should be sort of robust and it should be, you know, it should be.... and then if they want to put some sort of emotional reduce things cuz emotionally they feel they want to progress with this client.

And also, we were quite narrow cuz we tend to work on one project at a time. So, we're quite blinkered in what we're doing and we're not aware of the bigger picture. And I think that's where the sort of the final settlement meetings... the senior managers and directors who have got a ... you know, they're obviously hovering above us, can see the bigger picture, see what the future is, see what the past was sort of thing. They're a bit like a helicopter, aren't they? Hovering above. I'm down in little hole digging. I can't see what's, you know, all I can see is my… my thing. So, I think that's where they kind of then use their skill to then decide whether how much risk we put into it and how much, you know, profit margin, we applied to the project.

**Interviewee:** Okay. Thank you for sharing this. And it seems that you want to... you know, do your job more objective?

**Interviewee:** Yeah.

**Interviewee:** So, do you think you know, someone there is thinking that maybe one day, the estimator's work can be replaced by some algorithms or software. I mean, maybe they can automatically assess risk and calculate the risk allowance. How do you think about it?

**Interviewee:** I think you're quite right, because I think, I think really a lot of the... a lot of what we do really, I think it could be done by an algorithm, looking at previous projects or past performance. Because I think construction is quite… it's quite a slow evolving industry and many, many of the things, cuz I know we've... I've gone to great details before to build up risk registers and all the risks and everything. And at the end of the day really the directors want about 2% risk in the project. So, they kind of... you think to yourself, well, I've done all this analysis, but reality is there they kind of gut feeling is they want that 2% and that's what we're gonna have. So, I think, yeah, I think a lot of the things we do sometimes that probably, from first sort of... for the vast majority of projects, a lot of things are, could be automated. You know, could possibly that side of things could be automated, or a standard allowance put in.

But I think it's that... it's those projects where, you know, you get the one that's a bit of an outlier. That's different. It's got a very high-risk profile. Those are the ones that probably, if you didn't have people doing the work so much, you wouldn't necessarily pick those ones up sort of thing as... if you didn't have sort of people doing all the nitty-gritty detailed work, you wouldn't necessarily pick those up.

So yeah, I think I'll could be replaced by a robot pretty soon.

**Interviewee:** From my previous knowledge that you know, your role hasn’t been paid enough attention, I think.

**Interviewee:** Yeah, I think sometimes we're a bit, I don't know if you like nursery, all that Cinderella sort of thing. You're working away, doing the Cinderella. Is it the nursery, while they were working away, well, everybody else is off enjoying themselves? And yeah... I think… I don't know, I think everyone's role is important and I don't think you should get to you know, we're all equally important in what we do. Everybody has a different... I feel that. I've got my bit, but I also respect everybody else's involvement and think... I don't think we should get to sort of obsess with our importance.

**Interviewee:** So, you think nowadays what hinders the development of the algorithm or software we just mentioned? Because it's things that you feel it's possible, but why, you know, we haven't seen any development or something?

**Interviewee:** I think some of the problem is I think for... I think if we're talking purely at risk, I suppose, I suppose it's that sort of... suppose it's that people's trusting.... I suppose it may be that trusting computers and that sort of... I think because construction industry is fairly that quite old fashioned really in their thinking, aren't they, really? In terms of, we don't really necessarily take on new things that quickly. We tend to be either... I think that sort of things. I'm guessing in other industries you may have... you may well have looked at other industries in your work. You know, they probably have already gone to the point where they just look at algorithms and pascal. But I think, also construction... construct... no, cuz when you're working in a factory, building, you're building a motor car. Everything is very the same. So, you can get a good feeling for... You know, you know, that 99 cars will be perfect, one will be a problem and that will cost some money. So, it's quite easy to calculate that risk.

I think with construction there's so many variables you know, obviously you're working in the ground, so it's always notoriously difficult when you're working, when you've got to excavate and you're working outside, there's so many variables. It's a lot harder to... possibly a lot harder to calculate the risk automatically. So thankfully, they still employ people like me and keep me working.

**Interviewee:** Yeah. Do you think there are some differences, I mean, between human being estimators with the software and algorithms?

**Interviewee:**  Yeah, I suppose there is. Cuz that... I suppose you've... cuz we've got that... it's that sort of emotional kind of gut feeling, previous experience, I suppose. I'm not very, I'm so, I suppose, with that AI and things like that. They can develop... that can be developed into people, but it's also I think is that the group kind of like... say the group thinking. When you have a group of people and you're in a risk workshop and you're looking at the risks and everybody's inputting it, that I would think... that'd be quite hard to, for an algorithm to replicate.

But obviously my knowledge of computing is more sort of back in the old Fortran and, you know, I don't know much about computing. And looking at it, it's sort of, I think it's that being able to look at historic, looking back is the best way to be able to then project forward, really.

But I think a lot of projects, the risk is over complicated. We'd go to all these great exercises and spend hours looking at it really. They just want to put a, you know, it would be reasonable to say, 'right, this is an easy project. We'll put 1% risk in. This is a medium-size project. Not too bad. I think we could put two and a half percent in. And this one's a very, you know, difficult project. We could put 5% in' and, you know, sometimes I think we over overcomplicate things.
