# Supplementary material for: Critical factors influencing cost estimators’ judgements on cost contingencies in highway construction projects: An empirical study in the UK
Source: PLoS One. 2024 Dec 16;19(12):e0314665. doi: 10.1371/journal.pone.0314665 (PMC11649144; doi:10.1371/journal.pone.0314665)
Supplement: S2 File — (ZIP) [file pone.0314665.s002.zip › Transcription (Interview F).docx]

**Interview F-Meeting Recording**

**Interviewer:**  How you would become an estimate? Do you plan to do this, or something just happened?

**Interviewee:** No. Yeah, it sort of just happened. So, I did civil engineering at university, [University's name] and did work on various projects, railing, and highways jobs. Yeah, sort of a placement. And I did a year out, on site. And then I worked for a main contractor on site for two or three years. I then decided to join a smaller company. So, it could be more regionally based. Joining the smaller company, you got involved in a lot more than just the engineering side. So, you got involved in the costings, materials, programs, all that sort of thing.

I then went to work for a contracting business. So, basically predominantly a Korean business that had a contract on, So, we were laying [City] on motorways, A road council job, [name of airport] airports. And I guess, probably, 15... so, I've been doing it probably 17 years in terms of pure estimating in terms of, you know, being in more of a commercial role, that sort of started to happen probably after five years of working on site. So, I decided that... I like, you know, like to get involved in the costs and contracts and it sort of turned into more of a QS in straight project manager role. Yeah. And I guess I've just grown the estimating knowledge by working for a bigger company now. So, it's a slightly different to working for a regional company where it's very... it's lots faster pace, if there's more, there's more action in terms of projects are still little... projects normally work as subcontractors, but but as a main contractor I've been involved in, some of the biggest tunnelling jobs in the UK. So, [project's name], which took us 18 months to price, which is a big, super so up at the London. I've done a lot of tunnelling jobs to do with rail and a lot... lots of train depot. So, I've had quite a varied experience in terms of across all the sectors highways, rail, tunneling and airfield.

So, it's definitely something that evolve, and I firmly believe that you need to have to work for contracts or anyway, it definitely needs to have outside experience or useful outside experience. If you can't get that, if you come out of uni and decided that you end up, you know, working in a estimating role for a big contractor, one of the things you definitely wanna do is asking him if you can get outside and understand all the jobs build ,understand the risks involved, cuz if you can't understand it, if you can't picture it you had a very difficult process. So, yeah. I hope that... I've been long-winded maybe, but hopefully I've gave a favor.

**Interviewer:** Yeah, thank you for sharing these. So, you know, when you firstly come into the estimating work, does the company provide some trainings or courses to help you to understand and better understand your work, your job?

**Interviewee:** I think each company is different. I think if you work for a smaller company, you probably learn more on the job. So, you will have... you know, you love your labor, planner rates, love your program that you could... you know, you price it from first principles. So, it's more learning on the job and sitting alongside your line manager, I suppose. When you start to work for main contractors, management contractors, there is training out there. So, depending on what estimating software you use. The training for estimating software... base very much, very much, line manager driven it, very much learn on the job. And the company that we course on... I know this's, [company A], they do a course on understanding the different methods of measurement. So, whether it's specifications, hardware works, best measurement or assessing. There is a course on that which can go, you know, to help you. Okay, you build your knowledge because it's key, it's obviously key to estimate and understand your measurement works. I do think the scenario could be better in organisation...

**Interviewer:** Ok. Thank you. So, in your previous experiences in highway projects, can you pick one risk, which mostly happens, or you think it's more representative as an example and talk about all the things you will consider while assessing it?

**Interviewee:** There are various different ones but I suppose you could pick the big ones out. The big risk for me are ground conditions. So, it's difficult to took it in the early stages of a project. Well, you don't necessarily know the ground conditions because the SI (site investigation) has not been done, but you're advised a consultant, what you think of the likely outturn, price will be of that job. And that should be a biggest risk cuz you can only base your estimates on the assumption that, you know, the ground conditions will be fine. And if the ground conditions aren't, has contaminated or reuse material is there... there's a big risk. A: You get a really contaminated area. You don't know the volume of that material. So, the big risk is if ground conditions initially, and then it's access into the working area. So, can you get the labor, can you get the plant machine into the working area for you to actually build the job? And, if so, what temporary work will you need to be able to access the actual working area?

So, you know, on a drawing, you'd be able to take off quantities to it, either price or from first principles, knowing, you know, your labor, plant rate, your outputs or use some initial benchmarking range, depending on what stage, whose job is that. Then there's obviously a huge risk of assuming you can actually get into the working area to build the job. So, ground conditions and access for me are the biggest ...two big risks when you talk about construction.

**Interviewer:** Okay. Thank you. So, how do you think, you know, the controllability of ground conditions? The controllability, I mean, whether you can do something to control it. And if the status of it changes, I mean, for example, from controllable to uncontrollable, whether this will impact your judgment on it?

**Interviewee:** Yeah. I mean if we talk... if we talk of ... It depends at which stage of the process that you're in. So, I've done various different projects, some projects you got... particularly on the big government project, where they are trying to understand what that number is. But you've got very little detail. Your risk on that figure will be significantly higher than if you put together an estimate where you had detailed design and you are pricing the actual job. So, for me, risk is a... it's a process that you go through, which can start... you know, start out as a really big number and you manage and mitigate as you go through the process to the point where you get a detailed design, and then you can send that detailed design out to supply chain to get an actual price for that particular job. And at that stage, you will know the quantity of contaminator. You will have a good idea of what's contaminated and what's not. [construction company] to put a number to it.

It's right at the start of concept stage. You won't have any idea. So, you would just be putting in a typical, allowance of. You might assume that there's 30% of the material can be reused and 30% material has to get off site as contaminated. So, it's very much a... it's very much, in the early stages, very much a sort of experience stroke benchmark situation. Whereas as you come through to the design stage... detailed design process, you'll know actual quants. And the risk is that you've underassessed the risk for the material and increases when you actually get sufficient information back. So, I think, it's an area that often gets undervalued.

**Interviewer:** Okay. Yeah. Thank you. So, for you personally, how do you think of your attitude to risk, and do you think it will impact your risk assessment?

**Interviewee:** I think risks are very much a collaborative, collaborative approach. So, you can have some typical risks that you will find on a particular job. And you can obviously put price programming cost to that on what you think the impact of that risk will be to the specific project. But you also need to tie in with, the other stakeholders, whether it be... designers, the actual project delivery team. You need to know what your contract conditions are as well to understand what the risks are around contract conditions you are working to. As a lot of clients are trying to put all the risk onto the contractor.

And I have a pragmatic approach because there's a lot of... there's a lot of projects that very rarely, very rarely... sort of... you ended up building what you price. So, is understanding what the change is and what the risks are around the change. And it all comes down to the level of detail that you've got for your design and design... as estimators the design put so much pressure on you when it's likely... your design is giving you less and less time to procure, and you know, get prices from the supply chain, and price and programme it...

You know, it's very much a lot of pressure on the tendering team when the design is late in the process, which happens quite a lot. So, I always looked at what stage is the design? What do we know about it? And assess the risks around that particular moment in time? And always try... always go and have a look of the actual scheme that you pricing. You know, if you haven't got design, you can still go down to the projects and get feel for the area. What's the access or where's your compound gonna be? You can have a think about it. You can access the area your temporarily works. What we are required around that? Do you know any historical information around the area? Whether it will be, footplate or contaminate light... it has been contaminated? So, it's... I take a very pragmatic view on the risk, and I will put together along with the team all the risks that I think could come about and then take that forward to senior management.

**Interviewer:** Okay. For yourself, you think you are more willing to take risks, or you are more risk averse? Do you think it will affect your judgment on risks?

**Interviewee:** It's a difficult question because you might be risk averse and you might put a lot of risks into the risk register, which could give you quite a big number. That number wouldn't necessarily always be the risks that it gets carried forward to the actual, you know... the actual project. So, I think you can only do what you can do that your... is your role as an individual and present what you decide to present and then you talk to the directors on the... how much are their appetite for risk that they think it's the project liable to ... you know, to outturn basically.

**Interviewer:** Okay. So, do you assess risk in a team or make judgments in person?

**Interviewee:** No. It's very much... you might initially... you might initially do it on your own... you own take on it. You could probably do a bit of a brainstorming session where you'll put together risk register on all the risk that you can see and then depending on, what risk that is. You identify the costs involved, whether it be, you know, if it's going to be a delay of eight weeks, how much would that cost in terms of prelims. Material cost that you need to put in there as any additional labor and plant. What does that risk look like and then look at what is the likelihood, what is the probability that risk happening and then collectively and collaboratively then go, bring all your different ideas together form one risk register. So, it's very much... it might start off as an individual document, but they will then get put into the one risk register that will be normally owned by somebody to then change those risks, as the design develop, the project develop before the tender goes in.

So, you might have an initial risk register and then you might get further information from the client on the design, but then reinform our risk registers. So, it's bit of a iterative process. It's very much a team... it's very much a team... it should be a team activity. It's not a formal way but it should be.

**Interviewer:** So, for example, when you do it in a team, will people have different judgments on the same risk and for your understanding, why it happens? Why people, you know, will have different judgments on the same risk?

**Interviewee:** I think it comes down to experience and I think it comes down to what you've been exposed to. So, if you've got a... you might have a project manager on that job that has been experienced, you know, a lot of the risks they realize, so they might have a different view on the likelihood, something happening. There may be, you know, a commercial manager or an estimator. I think a lot of it comes down to experience in what you've been exposed to individually and how those risks were managed on site, because it is different. Isn't it, between a risk and between change? So, I think a lot of it comes down to your experiences as an individual as to how risk averse you are.

**Interviewer:** Okay. Thank you. Thank you for your perceptions. How do you know whether your team did a good job or how'd you know who among them, you know, whose judgment is the better one?

**Interviewee:** I don't... I think the risk, I don't think there's necessarily a better idea or not. I think it comes down to have you... have you collectively had all the risks that could potentially happen. And also, can you group those risks into one risk, can you categorise the risk? You might have four different risk items that could cause time delay, as an example. But obviously if you have a risk register, we've obviously got managed these risks as a project. So, have lots, lots, lots and lots individual little risks down on a document sometimes can be a bit unwieldy. So, can you... A: can you catch all the risks that you foresee and B: can you then group and characterise those risks to make it something manageable? So, I don't think it's a better idea. I think it's a case of getting all the risks covered. Yeah. I understand what all the risks are which is very much a team effort.

Cuz, you know, the commercial manager might understand in terms of commercial risks. A project engineer or project manager understand risk around buildability and estimator probably have a bit of experience of both of the risks... understanding. So, it's collectively looking at the job on its [unclear] and decide if you capture everything. So, I don't think there is a better idea. I think all of them need capture.

**Interviewer:** Ok. Thank you. So, how they make a consensus when they have different opinions sometimes?

**Interviewee:** That is purely comes down to your management structure then. So, it will purely come down to you collectively, it will be part of your governance process. So, you'll... you as a team will bring together what you think the risks are and where you think the risk... the risk... the cost of the risk is likely to be. And then it will be very much an internal, company governance stroke view on how those risks are presented back to the client.

**Interviewer:** Ok. So, I'm interested that in your highway projects experience, have you ever encountered a risk, which, you know, make you difficult to quantify the risk allowance for it? For example, maybe sometimes the context of the project is dynamic or very complex or sometimes there are some maybe, technical or design challenges for the project. So, can you take one risk as an example and describe how you finally approach it, pricing the risk allowances for it successfully?

**Interviewee:** I think a lot of risks, you can put some meaningful numbers to them. For example, there's a risk that the CBR value will be different when you come to build the job as to what you've attended it on, therefore the pavement debts and the contents and materials might change. So, you'll be able to put the increased costs of the materials in. You'll be able to understand what the time implications would be in terms of program.

The next step is what is the likelihood of the CBR value being different. So, if there's been site investigation, ground investigation, you've got actual test on the CBR. Then the chances are minimum. If the design hasn't, it doesn't specify or it's just an assumed CBR, then the likelihood of it being different is, you know, increases.

So, a lot of it for me always comes down to the design and what stage is the design and then, it's just a view on the likelihood of it being realized. I suppose, as a risk, which is why, you know, people that have risk registers still have Monte Carlo risk register-- a 3D point estimate... it all comes down to risk but it's very subjective, I think... on the likelihood of that happen. You got to look at the evidence, something you have to determine that.

So, for risk, I will look at the information. So, read the contract documents. I'll look at the design and then build the risk register, put around the contract docs and the design, being cognition of the categories of those risks. So, is it a weather risk? Is it a ground condition risk? Is it a commercial contractual condition risk? Is it a stakeholder risk? Is it down to traffic management? There's lots of different typical risks that you'd have on a project. Usually as a baseline and then further redefining it to meet the project, and then want to generate the risk register. Then look to put some numbers to it based on what your time-related prelims costs are and also what you potentially increase in material, labor, plant, all that might be for a particular item.

It starts of what your risks are, get a risk register that, you know, you think it covers everything that's involved and then apply the numbers to that particular risk. An early stage, i.e., if you're a consultant, I think that it's very difficult to do. It would just normally be a banded lump of money that risk will attract.

So, if you're thinking of like your highways, the highways projects at 50 million. If you've got a highways project, that's got an actual design and you can price and program it and then the risk associated with that particular project. Versus if somebody said, right, I want to build a road. It's so many meters long, and we think it's so many meters wide and assume that, you know, the depth of [unclear] is 300 miles of depth, [unclear] is 500 miles, assumed the drainage, obviously that stage, in the process, the risks involved in that particular tender and estimate is a lot higher than the risk involved in a job that's gone through the whole design process, and you've done all you surveys, you booked all your road space. You know, it's very dependent on what stage the projects are.

**Interviewer:** OK. Thank you for sharing. You know, acquiring like rules or principles from your previous, experience, made for yourself that can be used in risk pricing?

**Interviewee:** No. I think. I've got a risk register template if you like. Which is got, you know, categories of risks. It's almost like a prompt list to be honest. You know, in the first instance case, using that going through, and you know, building on it, using previous jobs. You know, you might be doing a job, that's got a risk register that's similar to... your next job, you might use the risk register from a previous job as a basis, but then you're very much tweaked that according to which other job you are pricing. It differs from one project to another, but you would use a tip. You can use knowledge management or previous risk registers that you've used to use as a starting point.

**Interviewer:** Okay. So, I'm, I'm curious, how do you think of the idea that maybe one day some algorithm or computer softwares, they can replace the role of estimators and they can automatically price and calculate the risk allowances?

**Interviewee:** My own opinion... I don't think it's possible. I don't see how you can get computer to decide what the risk on the project is because they're not gonna actually build it. The computer not actually... it just doesn't actually know what the constructability of it, the actual human risks, the risks around actual price and program. I don't... I think it's... I think this is very... you can have it run a calculation and you could probably use it as a means to give you a figure like... something like a Monte Carlo exercise, isn't it? But I don't think you can just use that on its own as a stand-alone piece of software information.

It's the same, it's the same with estimate, you know, you could get a model and put some typical rates and costs to a model. But ultimately, if you don't end up building it in the sequence that the model suggesting, or you have delayed your programs, delays on site, for whatever it may be, or something's slightly different. The model didn't pick up, like temporary works is a good example. I don't see how you can just automatically rely on a computer software or a model or anything like that to rely on in its sole entity. You need to have the experience, [unclear] as well.

So, I mean, you know, if it's in terms of an algorithm being developed to calculate risks, then I think, yeah, it's never, it's never going to be a bad thing, but you don't think you can just purely rely on that.

**Interviewer:** Okay. So, you know, what's the difference? I mean, the outcomes calculated by like the software and the outcomes given by estimators?

**Interviewee:** The differences would be experience, knowledge and understanding in the actual job. So, if you've got a risk register that risk register can't understand the drawings. So, how can they understand what the true risks is? The risk register won't read the, you know, the ground investigation report, so how can they gonna know what the likelihood is of that ground being contaminated and the computer software won't know spoken with stakeholders for example. So, they might not know, is it a particularly sensitive job in a particularly sensitive area of the country and the likelihood of being protested and the delays due to disasters is high. Well, the algorithm won't pick that up. So, you still need to have very much on the ground informed calculations to whether you think that's likely happen.

**Interviewer:** Ok. Thank you. So, for yourself, you think, what knowledge or skills you think are mostly helpful for you to make good judgment on risks and risk pricing?

**Interviewee:** All-round knowledge, really, you know, understanding what's happening in the market. So, for example, at the moment, you know, you might have a job that you are tendering now but it's not gonna get built ... say years time. So, yeah, you look at price increases and materials, things like that.... What's happening in the economy to drive price increases. You know, in some years there won't be a significant price increase but currently because of various different economic factors, like Brexit or Covid or whatever it might be. So, it's very much an experience. It's looking at each individual risk and use your experience to understand what impact of that gonna be.

Typically, you know... I don't know... say, you'd be... say you are pricing a tunnel which based on a design that is assuming certain ground conditions and then we actually start to come to build the job... you know the water table is high, the ground condition is slightly moistest than you thought. These are all realist risks. You know, if you've done a similar scheme in a similar area, then you can use your knowledge and expertise to, you know to inform your risk calculations, and decision making really.

**Interviewer:** Okay, thank you. So, how do you think about the idea that when you're pricing risks, it's always a financial application on the risk, I mean when you pricing the risk, you just mentioned that you will think about it's a likelihood and impact. So, will you only think about it from a financial perspective or not?

**Interviewee:** No, I'll start ...I start the other way around, I think about what is the risk? What is the actual risk we're looking at? And then what would be the financial, what would be the likelihood of that risk happening and then calculating what you think the cost of that would be. So, look at your risk, price your risk if it's going to happen, definitely going to happen and then look at what the likelihood of that risk being is, and then your cost is gonna affected by the likelihood to give you a... I won't say realistic but... an idea of what the likely cost is gonna be for that particular risk, and obviously added them all together and that will be your risk figure.

**Interviewer:** So, will you only think about the financial impact or you will think about other impacts which may bring about by this risk when you pricing the risk?

**Interviewee:** I think about the other impacts, but ultimatly those other impacts will have a financial element to it, anyway. So... you won't only think about the financial impact when you think... when you're pulling your risk register together, ultimately, whatever risk you end up, thinking of collectively as a team, realise, lots of other form of financial impact.

And, say for example, so you build a job which is gonna to be noisy, and you know the potential risk is.... you have to do in certain parameters, anyway, it could be a risk it is going to affect, you know, nearby, residents or you will be affecting the college in some way. So, you'd look at how can we mitigate that risk then. So, one way could mitigate the noise would be to put an acoustic shed for example or an acoustic fence to reduce the noise. So, you thinking of the stakeholders in terms of the risk and noise but then your mitigation is what can we do to mitigate it? And then you'd have to cost in, you know, what would be a cost of a ... accuse a shed shuffling if you dropped in shift middle of the city, how much is... X amount of accused bury gonna cost you? So, you won't always, you won't just think of the cost, you will think of the risk first, and then how you gonna mitigate it? What does that mitigation costs look like and that would be going into your risk pot? If that makes sense?

**Interviewer:** Yeah. If you tell someone with very little experience, all your experience in highway projects, do you think I can be good at-risk pricing as you do?

**Interviewee:** No. I think you need to gain the experience and the knowledge over time. I think you can share that knowledge and I do think that particularly on the bigger jobs, you... it's worthwhile, I have a risk manager on a big job so somebody that just purely looks at risk, but that risk manager would be very much collaborative with the estimators, the delivery team, the commercial team to understand the contract conditions. So, it's not a role that you can be left to do on your own devices. It's very much a role that that needs input from everybody involve really.

I do think there's definitely, you know, if you are on a smaller job, while you are pricing whatever element of it, you might just... you know, the supply chain would have a different view on risk as to what you would as a main contractor on large infrastructure jobs, big highways jobs. I think it's useful to have a, you know, a risk manager that owns the whole sort of process and the register and different individuals to feed into it.
