# Supplementary material for: Critical factors influencing cost estimators’ judgements on cost contingencies in highway construction projects: An empirical study in the UK
Source: PLoS One. 2024 Dec 16;19(12):e0314665. doi: 10.1371/journal.pone.0314665 (PMC11649144; doi:10.1371/journal.pone.0314665)
Supplement: S2 File — (ZIP) [file pone.0314665.s002.zip › Transcription (Interview G).docx]

**Interview G-Meeting Recording**

**Interviewer:** Firstly, I'm interested in how you become an estimator. Do you plan to do this or, you know it just happened?

Interviewee**:** I kind of fell into it, also I've been doing, it's picked up in, at the company 16 years now. I didn't have a clue what I wanted to do when I left school, knew I liked construction, the idea of it and kind of fell upon it really. There was a job going in the company I worked for [Company A]. I was in [A place], I didn't know what it entailed, but was working in the estimating department. And that was just going out for material prices, sub-contract prices, bringing all the ingredients together for the estimator, so working with them. And then I kind of... I saw it as a progression route that I liked it... I liked, I liked the role and I liked what seeing what the estimate is produced and how you are involved in so many different aspects even before a bucket is put in the ground.

So, I kind of put myself forward for pushed and pushed to be brought on. So, yeah, then I became an assistant estimator as well as doing the inquiry side. And then did that for a couple of years. Then I went into planning for a bit and then out on site. So, I had that for about a year and came back to estimating then was worked out a few different regional offices. So worked away from home. Did a variety of different projects, worked for the environment agency. Yeah, and then got made estimator, and then just gradually just went out now to senior estimator next steps, probably going to be managing estimate. So that's the plan. That's the graph. Yeah, I didn't, I didn't even, I didn't have a clue that estimating. Everything you see, as you walk around in everyday life has had someone estimated, planed it. You just don't realize until you're in that industry, you just don't realize the effort that has gone into... you just see roadworks turn up and you just think someone said, go and do that. And the amount of work that goes on behind the scenes to get a job off the ground is insane. No, I really enjoy it.

It's stressful at times. When you've got a deadline because you have deadlines to adhere, but Yeah, I enjoy it. I really enjoy it. But yeah, I kind of just fell into it.

**Interviewer:** You just mentioned that, you know, you learned something about estimating, do think they are helpful or useful where you really do the estimating job?

Interviewee**:** I don't know. It's just such a weird career that you have... well, I've got this a little bit of an argument going with the director of our company at the moment. Cuz he's pushing this intensity of estimating training program and I don't think you can do that. I think it's a learn... It's an experience. It's a combination of... like just doing the job and then site experience. Cuz it, the old-fashioned way was the old guys that have been there, built it on site. We're fed up, we're sitting in our cold side cabin, wanted a little bit of an easy retirement. They came in cuz they've got like experience, but nowadays it does appear to be more of an academic route that some people take, which I took coupled with a bit of site experience, but the old way of just it's a, the old guys that have been out on site is I think it has been gone now that has I think it is a career progression that you can... you can learn, but I think it's only learned through experience. You can't be, I could teach, I could teach you today how to drive the software and it would be fine, but it's just, the software is one thing. It's what then sort of is going on in your mental processes of how things are put together which I'm trying to get across to the directors that they're trying to push these young trainees. And it's just dangerous, I think. So sometime... whether you can learn it or not, I think it's more of a learning experience. I think that's probably ...

**Interviewer:** Okay. Thank you. Thank you. That's good. So, you might have many highway experience. So could you pick one risk which happens most, or maybe you are most familiar with as an example, and talk about... I like to know what you were thinking about, while you assess the risks? I mean, the probability of occurrence and its impact.

Interviewee**:** Yeah. So... when we're pricing up jobs, you're kind of, we price everything from first principles from scratch every job. So, along the way of putting a price together, you are kind of taking into account things like ground conditions, working around utilities, the actual fundamentals of building the job, which have their own inherent risks. So, we kind of build that pot up of money and then we have this thing called a risk and opportunity management plan, which is kind of the things which are a little bit more difficult to quantify.

You can quantify certain things, but things like weather, weather is a massive thing for us, because we're doing our job outside. We're not in a factory environment where it's completely sterile. So, weather is a big one, particularly with big... on a big highways job. When you've got earthworks, you've got a very narrow weather window and you've got the most expensive bits upon on your site at that moment in time. And if it rains and it ruins the site, you can push on, but then you could then... you won't be hit in the outputs. You then will be destroying the site and it probably take you another week or two, once it dries out to put things right. So, weather, it's one that we always come up in our risk and opportunity management plan.

And because we work closely with the planners and the construction teams. We get everyone's point of view, put in, and I build up a cost for losing... say, if it's over a year... a year project, maybe losing a month, month worth of downtime. So, build up that cost. And then we look at the likelihood of that. And particularly the... when we're doing certain operations, can we mitigate it by doing the earthworks in a different order to mitigate that risk. So, we don't have to put all that money in because it's a diff... it's a balancing then. Because you're trying to... you've got to be competitive, but then you've got to be realistic.

So, it just normally, we have these big tender assessments where I'm presenting what I've done and the risks that I've foreseen in this risk and opportunity management plan, and then becomes a big discussion as to how much of a likelihood and the impact, and we've got the spreadsheet we graded on low, medium high thing is very high. You put all these in and it spits out a medium or low, or what.... That brings for a percentage of the value of the say, the month's money. And then it's just a... it's a game of opinions. And if you get overshadowed by the directors, if they want the job, they can say, 'no, we're gonna yes. Thanks for flagging that risk. Hear what you're saying, but we really want this job. We can't afford the competition we're up against. We can't afford to build all of it in. We'll build a chord' or something like that.

So, there's a pile of money. That's there for... excuse the pun, a rainy day, but yeah. There's a little bit of science behind it, then it's gut feeling. And then it's then down to the directors to get their approval sign off on how much the business case for winning the job, because nothing ever starts when it says it's gonna start, very rarely. So, you can... you'll set your parameters that it starts in the winter, but experience tells me that things never start when they decided they're gonna start. They haven't got planning permission. There are legal agreements, which haven't been solid. There's a lot of things that might normally delay. The job I'm working on now should have started in April this year. And probably now looking like it's going to be more like January next year. So, what the... why I was looking at it back then, or April start is now totally different, to now January staff. So it just, it's just ever changing. It's a wave, the wave environment. You can only issue your best guess at the time with the information you've got. Yeah. So, and it's a pot of money for the site guys to go build the job, in any which way, they possibly can within the budget. Totally.

**Interviewer:** Okay. So, for example, the ground condition, as one risk which, you just mentioned and you said you will fill in low, medium, high as its probability and, some values for it. So, what things you were thinking about when you rate?

Interviewee**:** So, for those who are coming up with the value, I'll be looking at the cost of machinery that will be employed at the time, the cost of the blokes on a like a weekly cost and our time-related premiums or our supervision. So, that will generate a pot of money for say a month. And then we'll then start looking at when... we look at the program as to when we're doing these critical operations and then think, well, do we need to include all that because we'll do... say we'll do half the earthworks in the better weather and we'll do... so then you then start looking at the actual risk of that particular item.

And then you... we then put that money into those items in the bills of quantities. So... cuz the danger is, if you then just spread it over all the rates, decline and then emits a load of work. And you've got a risk part, which is spread over the whole job. And you've now doing half the job where you want it, kind of not a target on the risk items, and then that's where we put it on the program as well. So, the planners will put time risk into the earthwork’s activities. So, we'll try and target where we put those risk items. So, one, we know where the money is. So, we can keep a track of it. So, the risk and opportunity management plan are a live document, which... it... as when you win a job, you'll hand it over to the site staff and they've then got it and then they'll work that through. So, they'll then look at mitigation measures to try and lessen the impact, maybe we program something, maybe use a different data kit. They will then know that they've got pot of money to do a particular item that we've felt would cover it and they look to them better it.

So yes, the weather one is normally just a sort of... a bit of guess, best guess, cuz you never know what it's gonna do. We could have a really dry winter or we can have the worst winter that... I probably last year was horrible.

**Interviewer:** So, based on what things, you know, to make this guess?

Interviewee**:** It's just years of experience. So, there's loads of people around the table when we have these meetings with the directors. So, it's just, whoever can shout the loudest almost and who can put the best case across. So, yeah, I will present it. This is my opinion based on my experience and when I visited site, what I've seen. And then it's just a case of justifying that. And if anyone else has got a better suggestion or if they just totally overrule me.

Our whole point of... it's weird. As an estimator, you're coming... your job is to come up with the net cost -- what asking cost the business, and then kind of the directors can either make or break it. They're looking at the risks. So, the business as a whole. So, globally looking over the whole thing. So, like every job I take to them, they could just say, 'no we don't want it'. And they'll put a massive markup on it. So, I could go a whole year and not win anything. Now is that reflection on me that's as an estimator or is that just the reflection of the work types up and given and where the business is at this moment in time?

So, I can just only present what I feel is the most efficient way of doing something and the realistic view of the risks. I can get overruled in this meeting, these meetings and they could put loads more money on it and goes in and we lose the job. Now, it's not saying they're right or I'm wrong. It's just a game of opinions. And they're looking at the whole, cuz they're looking at the whole turnover of the business, the business needs. I'm kind of just focused in on, this is my little job. The business has said, we want to do this because it's obviously arrived on my desk. So, I have to give it the best possibly go and just try and weigh up.

So, as I say, the risk and opportunity management plan is... we're not always looking at the... you can get quite negative risk, risk, risk, risk, risk, risk. And we look at the opportunity. So, we look at the flip. So it's a balancing view. So again, we might put some money in for some weather risk, but then I might think of a value engineering way of doing something cheaper, better, quicker. So, it's kind of trying to get that balance. You can... there's a danger that we get very risk averse. That we like put money in for this risk, risk, risk, risk, and just build up this massive pool. You're never going to win anything. It's a game, it's a game of winning things, but you've got to win at the right level. So, you have to take a balanced view of... 'yes, there are some negative points, but if we get the job, we could use something different, do something different. We do stabilization. We could use a different bit of kit, which would speed up the program'. So, you have to kind of look at all those different and just get that balance just right. So yes, put some money in for risks, but also look at the flip side, what could go right that our competitors might not think of. And then that will then gauge the risk profile for the job.

And then it's just me presenting it to the directors and then like... they can kill it; they can say, 'no, see what you're saying, but we won't. If we win the job, we won't make a load of money on it'. So, they'll put a really high profit margin on it. Profit margin in civils isn't very big anyway, but we’re working about 2% or 3% profit on jobs and you don't have to have many days of bad weather and bad luck to get that written off which is not great, but... yeah, there's a lot of risk in this stuff... Stats is a big risk, the utilities, that always comes up. I don't know. Why it shouldn't be so difficult to arrange, sort out utilities but it's so disjointed getting them to site, getting them to perform.

Actually even, speaking to them at tender stage, they don't want us to know because we're not the ones paying the bill. So, they only want to speak to the client who's paying the bill, they're holding off, paying the bill until they're spoke to us and got what the infrastructure is going to cost. It's a chicken and egg scenario and it always comes down to a few cables and pipes that are in the ground, which have to be diverted, protected, moved. And I don't know why it gets so complicated cuz it's shouldn't be. But it's just a thing... I think just cuz there's so many moving parts and so many different people you have to talk to that no one fully grabs it and runs with it and looks after it. It's so disjointed. And that is always a massive risk for us as a business because that there got certain requirements and they've got certain leadings and they've got certain procedures that we can't just say, 'we're going to go have your pipeline'. They were like, 'no, this ours, you can't do it'. And that just froze us. We just stopped. They set the rules. So, that's a huge risk for us as a business cuz we're always digging in the ground as you see. We do tend to hit this, so, sadly.

**Interviewer:** Yeah, it seems it's a really complex process.

Interviewee**:** It's just so many different moving parts. You've got gas, water, electric, and the records of where they've been laid are never very accurate. They've got so many different procedures for working in and around their pipelines. They could just point blank, say, 'no, you're not going to build over a pipeline because we need access to it in the future'. So, then it's like a total flip on its head of a total redesign and maybe a full diversion that needs to be done. And that can just blow, if you've already started, that could just destroy your program. You could be waiting years for a diversion to be done, and it's never, it's never easy. Really isn't.

The present job, I'm looking at with... We've got BT diversion and they're stipulating a 23week leading to divert some BT. Our whole program is derived from that, on that information. And we can't get them to better it because we can't pay... no one's paying them yet. So, they won't lessen it. So that, we're getting pressure to reduce our price. But we're only basing on the information we've got. It's just so convoluted and just so too many things, too many things up in the air. It's not good.

**Interviewer:** Yeah. So for you personally, how do you think of your attitude to risk? Do you think it will affect your judgment of risk?

Interviewee**:** Definitely. So you can get... cuz we have to, then we have to read or give all attended documents, all the reports, unexploded ordnance, stats, flooding, ground conditions. You can get quite a tainted view. You're sort of submerse yourself into all this, all these reports, and try and take in all the salient points. It can taint your view, especially for the job you're not particularly... you don't particularly like the look of the information's a bit all over the place. Because if the information so hard to find you then start being overly cautious, I think. I'd prefer to have a really nice set of documents, which is clearly laid out. When it's just a mishmash of stuff sent to you and you've got to go and find it all. That straightaway gets you a sort of... you'll be on alert. You're a bit more cautious than you would normally be.

So, normally I think I'm quite bold with how I price. I'm quite optimistic. And then I use... so in ... that's in my pricing, and then when I... I then looked at it in the risk and opportunity management plan, which is a separate Excel document. I then look at the more negative points that way. But as I say, if I had to search for things, just to get the price together. It can taint my view and I'll be starting to be cautious already in the pricing. So there could be a case of, there's already risk built into the price before and then do my risk and opportunity management. So, it's kind of maybe a little bit of double bubble. I personally think it's down to the info. If you've got some really decent information that you can... someone says, where is this? And you just open a document as bang, you can find it, you have confidence and what you've been given. It's kind of a... one of those really... If you have really good information, you'll get a really good price. You haven't got good information and it's a bit all over the place. Naturally, you'll be very cautious because at the end of the day, I've got to protect the interests of the business.

So, I personally would push it further down... push it back to the client and the consultant that they need to get their documents as concise as possible. And they will then get the best price and we'll look at it. We'll be in a bad position. Yeah. It's based... it's definitely got to be on the information that you've been given. I think. Which isn't very good. Sometimes, some consultants are better than others. Let's put it that way.

**Interviewer:** So, you know, you work in a team, you just mentioned. So, between you and other estimators, why sometimes will you have different opinions on the same risk? So, for you, you know, why, what factors you think make this difference?

Interviewee**:** My team, we would all come up with a slightly different price. I'd like to think there would be all there on their bags, but they'd all be different. And that would all be... I think it's just based on your personal experience, when you've been out and see it, been it... seen it, done it, touched it, felt it, that's really where you get your... it's the gut feeling of... so, all of it ... it's just that... it's just based on your experience. That would be the only difference. So, and the people that maybe have been out on site more recently than others they'd have a better view of the, the kit that we've got available now. Whereas the older guys are probably recalling memories of years gone by and things obviously evolving. And it's just a case of other jobs that you've priced more recently that and things you've learned along the way.

So I just say it's just based on years of experience, gut feeling. Which... I know it's not an exact, so, I was always told this is not an exact science, there's never a right or wrong answer in estimating. There's better answers and worse answers. But my answer is, my answer is mine and someone else can provide something and that's their opinion.

And it's such a weird career and profession because there isn't a right and wrong answer. You... if I price something, I'll felt that's the price based on my experience and taking off measuring it. And that is the price, but someone else would have a different... could have a different opinion. And it's just based on their personal experience as well. And if maybe they've been caught out on something in the past, they might have... they might be a little bit tainted because they might've lost some money on a certain job. But the whole... the whole thing should be on a loop. I think we should. I'm not... I'm trying to stress this. We are getting better at it.

Then that needs to be that feedback. You price a job, we win it, we build it, we learned some lessons that then gets feedback. So, you're constantly refining the process. So, if there was a mistake made and there wasn't enough allowance for a certain risk that needs to be feedback in because we don't want to keep making the same mistakes. We need to be learning all the time. I think the danger is when you stop learning and I never want to stop learning. Every time I do at another job, there's always something new to learn. There's always a new. I've always be speaking with another member of the construction team. Okay. Their opinion.

So you just build... you get little snippets from everyone, and then that builds into your own personal experience, that's what I've found anyway. So, I just want to take much experience of people that have been there, done it. And even when I've gone out on site, I'll talk to the digger drivers and the people in the ground or on the ground doing the work, because they're the experts at the end of the day. You need their opinion. So yeah, I think the reason that you would get different price from different people, it's just their own personal experience in the industry. Hopefully that answers your question, but they tend to pop a little bit.

**Interviewer:**  Thank you. You know, you're quite honest, you know, sharing your perceptions. I'm so grateful for that. And so, in your past experience, have you ever encountered, you know, a risk, which you feel a little bit difficult to price the allowance for it, risk cost allowance for it and how you finally approach it?

Interviewee**:** Yeah. So, one of the risks that I had on a job or.... can't remember where it was now, but it was about, it was unexploded ordinance. It was... we were doing piling works. Is it... it might've been in the [place]. [Place] got heavily bombed. You get all the reports, about unexploded ordinance. We were in the tidal zone. So it's just... it's just a total guess. And the risk of hitting something is... if you hit something it's catastrophic and it's like, well, how do you quantify? I can... I can put mitigation. I can put a watching briefing there. I can put surveys in there, but what if something just washes in overnight into our pipeline and it just got to a point and I ended up having to... to push it further up the line to the directors, because it was such a... I think it was Randy and the Royal arsenal. So. It was a... it was a pretty... it was a military establishment for us, which wouldn't have been absolutely hammered in the war. So the risk was huge. But yeah, the impact of the risk would have been huge and the likelihood of encountering it was quite high. But we didn't encounter anything thankfully, but you just never know.

And I had to just push that to directors and other people, because it was just... it was above my pay grade. Cuz if we hit something that would have been [unclear] and yeah... Yeah, sadly you have to just push it up the line and someone else estimate the call. It could get to a point where if that risk became apparent early on, we might pull out of tendering for. Some risks can be too great.

Yeah. Another job I had was... this last job I've just been doing was a full design and build, and the contract was so onerous that the risks for gaining adoption, RSA three and four was all on us. And you're talking about, works on a dual carriageway, which in 10 years time, there could have been loads of rear end shunts, and we then have to stop build, widening the embankments to create a bigger stacking length for cars coming off the dual carriageway. And that was a deal breaker for us. And we had to push back and say, 'we've got reworded this contract. Otherwise, we just can't sign it'. If we designed it, we would have been liable. So, you can even get it reword out or you can just put money and against it. Yeah, those were... that was... That has been a big risk on that job because you just were liable for like 12 years to get the thing adopted. We wouldn't have got any of our retention released on 20 million pounds worth of work. That was huge. Yeah.

**Interviewer:** So when you mentioned the, you know, the impact of a risk, do you only mean the financial impact, or do you think you from other perspective?

Interviewee**:**  We tend to only be looking at the financial impact. We do. I would put risk registers together. We do sometimes look at that.... There's that health and safety issue or environmental issue. We flag it as a... we've just put a little code next to it, or E for environmental or quality or health and safety, but genuinely as estimators where we're mainly worried about the pennies and the pounds and wanting to put a financial allowance against it, to cover off that risk.

I get, yeah, I get there's environmental risks and things like that which, yeah... but as estimators, we mainly concentrating on the numbers at that stage.

**Interviewer:** Okay. Yeah, I understand. So, for you, how do you think the, you know, the risk pricing job, how do you think the idea that maybe one day, risk pricing job can be replaced by some... you know, the algorithm or some computer software.

Interviewee**:**  I don't know. I've been thinking about this because my brother [Brother's name], he works on... he's on an [Power station] at moment. He's working there and he was talking to me about, is it the Monte-Carlo. There's Monte-Carlo risk. I've heard of it. I've not ever used it, but it would be really nice if you could put a load of info into a computer and it spat out a number... I don't know. I don't know whether that would be... that'd be possible how..

Because we're not robots on the ground. There's always that inherent risk of someone doing something silly on site. And that just completely blowing some risks that you didn't foresee it. You've got always got human error. So I think you always have to have... it'd be great, if you could put all the data and everything into a machine and it spat out -- this is the risk you need to allow. But there's too many variables like the weather, the... you just can't predict it and you've got people on the ground. There's always that human error of things that are gonna go wrong. Things are gonna not be in the right place. So I, I don't think that can... it could probably do it to a point, but I'm thinking that it would completely cover off. I think it was still needed human indirect intervention to have a final gut feeling on something based on experience. I don't think you can give that experience to a computer fully. Otherwise we'll be out of a job.

**Interviewer:** Yeah. So, you think your... you know, the risk, your risk pricing job is not an objective one? Is it not more subjective?

Interviewee**:** Yeah. It's much more subjective. Yeah. Yeah. Yeah.

**Interviewer:** So it just mentioned that every time you will reflect on the project, what you have done. So, have you made any roofs of some or, you know, the principles,for yourself in risk pricing?

Interviewee**:** Based on jobs I've won; you get feedback based on allowances which you have made. Again, it's not hard and fast. It's just, again, subjective and subjective views from people who've gone out and built it. The site we will always say you've never allowed too much, will never allow enough. Because they always want more comfort to make their lives easier. I can't think of anything major that I've had to sort of reflect and revise forward on risk pricing.

But normally when we go to these Tender assessments, the guys have all been... they've all been looking at it. So, I've been looking at the financial points, planner has been looking at it as a timeline, the constructioner have been looking at it as buildability. We've all been looking at different from different perspectives and we all have an input into the pot. So, you get a good blender of... But my opinion and probably two other individual opinions and we are working independently even more so with COVID.

So, there's no conclusion. So, you're looking at it from, with fresh eyes and it's like a big melting pot, and just, everyone's pouring their ideas in and we come up with the best idea. And then we present that to the directors who then might have a totally different idea. So, you get a good... it should never be just on one person's opinion, although sometimes it feels like it is, there's an estimator. You kind of feel like you're making all the decisions. Hope that answer your question.

**Interviewer:** Yes. So, you know, for you, what knowledge or what skills do you think are mostly useful, helpful in your risk pricing job?

Interviewee**:** Good analytical... you've got loads and folders to go through 10 documents. You've got to be able to sort of take in the salient points. You can be forever reading every line. You've got to be able to really got pull out the salient points quite quickly. There's you are on a tender, we're on a deadline. Good organizational skills, so, and sort of documenting it. So, documenting risks as you're pricing stuff, making a note, adding it to risk register. That's key. Time management is key. Allowing sufficient time.

I tend to sort of price my risk and opportunity management plan as I'm pricing the job. So, I'll come across something that I think, oh, about flag that. And rather than some people will price the job totally over there and then they'll do it all of the end. It was, I think, needs to be done at the same time. It's sort of a one... one doesn't function about the other. So, I kind of think you need to be looking at it. You can't just... cuz you might forget and you might get to the end of pricing all and then 'right, I've got now... I've got to now do the risks' or you come across the risks as you're reading the documents, as you're reading the drawings, as you're looking at the detail. So yeah, I think you need to manage your time really well good organizational skills, be able to skim, read by quickly pull out the salient points.

And I've had that personal experience out one site and spoke to the people on the ground that I've come across these risks and I've had to try and find a solution. And just be a good listener and always try and learn and listen to people and take their opinion, understand it, and then try and yeah, just try and understand people's opinion. Everyone's got an opinion. You've just then got to try and get a good balance going. That's what I think.
