# Supplementary material for: Critical factors influencing cost estimators’ judgements on cost contingencies in highway construction projects: An empirical study in the UK
Source: PLoS One. 2024 Dec 16;19(12):e0314665. doi: 10.1371/journal.pone.0314665 (PMC11649144; doi:10.1371/journal.pone.0314665)
Supplement: S2 File — (ZIP) [file pone.0314665.s002.zip › Transcription (Interview H).docx]

**Interview H-Meeting Recording**

**Interviewer:** For you, how you become an estimator? Do you plan to do this or, you know, it's just happened?

**Interviewee:**  I did a degree in geology in [University A] and I joined a construction company that does not exist anymore. It... so it got bought by another company called [Company A], and they were taking people on, into the construction company that didn't have an engineering, or a construction background and I was due to move around the various different parts of the company and move. And one of the first parts I visited was the estimating department, and I enjoyed cuz I'm quite competitive person. I enjoyed the idea that you would be pitting your wits and your brains against other contractors to try and come up with a best solution.

So, I then did a... while I was working, I did a master's degree in construction management through [University B] and spent some time out on site but it was time out on site, knowing that I was gonna go back into the estimating department. So, I wasn't doing any engineering. I was just studying, how quickly, how many people, how often it was doing, how often they were standing, still doing nothing, that sort of thing. So, to answer your question, it was almost an accident to be an estimator.

**Interviewer:** Okay. So, I like to know, you know, you just mentioned that in the first company, they help people, you know, without the background. So, how they do this? Do they provide any trainings or courses?

**Interviewee:** No, that they... that was about... I think there were about 9 or 10 of us and they paid for all of us to go on the, the construction management degree because that covered a lot of aspects of construction about measurement, construction law, that sort of thing. Without going into the detail that you'd need to be an engineer, or you'd need to be... So, it gave a very broad background of the construction business and the construction industry then help people what they were doing. And then when I then moved into the estimating department, I will go on various courses, not necessarily to do with estimating but to do with ... so, if there was a presentation about sheet piling, about all the different aspects of work, so trying to learn like that.

**Interviewer:** Okay. Do you think these, you know, do you think they are useful in your risk pricing job, work?

**Interviewee:** I've used my geology degree and the knowledge that I got in construction, cuz obviously a lot of what we did, what we do is in the ground and understanding about how the ground works and the different sorts of rocks and strata wall. So, that was useful. The construction management helped, particularly on the contractual side and help understand... So, in essence, start to understand how the contract, because when you look at a contract initially, it doesn't look like it's… a register of risks. But that's all a contract is really. It's a statement that you signed up to that says we're gonna take on this contract here. So, that means that we're taking on all the risks that are there and anything that we don't take on, the client takes on. So yeah, it was useful and that sort of gets you thinking about, can we... do we as a company want to take that risk or is it something that we need to ask questions about the client.

**Interviewer:** Okay. Okay. Thank you. So, in your experiment in highway projects, can you pick one risk as an example, and, you know, talk about all the things you will think about while you make assessment on it's, you know, probability of occurrence and its impact on project?

**Interviewee:** Looking at a job recently, on the [position]. And that was interesting because it was running through an area that had a lot of different types of geology. There was... it was running through the limestone that was quite fractured. And then it moved quite a lot, not recently, but in geological history. So, the strata that we were going to be building on top of was fractured and faulted. So, we needed to be mindful of that and how we would engineer it.

And then at the northern end of the job as well, there are what we call a Fuller's earth and it's sort of a clay, but it can flow. You never know when it will flow. It can be in a solid state, 99.9% of the time. But if for argument's sake where then making a big cut to put the road through and we disturbed something, the underground water table gets moved or adjusted and that could have a really, really big effect on what the solution needs to be.

And when you're in, cuz it was a design and construct job, or it's studies. When you're in the very early stage of a design and construct job, you don't necessarily… the designers don't go into all of the detail to solve it. So, sitting there as an estimator, whilst we don't have the detail about what we need to do. We need to be mindful that there's a problem that we either need to engineer out, or we need to make it a cost allowance for doing something. And it’s sort of the experience over time of looking at things like that and saying, well, I know you've said that we're going to build the road there and it's going to be three meters down in the ground, but I know that by doing that, we've got... we're taking on the risk of ground conditions and things that could happen there.

So, I think a lot of it is sort of experience based. Some of it can be, I don't know, almost like an intuition. You get a feeling that something doesn't seem right. Even if you haven't got huge experience in certain areas. So, you, you know, 'oh, I'm not so sure about that'. So, you then find the right people to go and ask the questions of. So, does that answered that question?

**Interviewer:** Yes. So, you know, people always mentioned experience and intuition. So, for your understanding, you think, you know what makes up your intuition?

**Interviewee:** It's strange because when I said intuition, that sort of, you get more intuition as you get more experience. If ... because, you've seen more things, so you're sort of more aware that there are more things that can go wrong. So, you, rather than looking at a piece of paper, you're looking beyond the piece of paper and the more times things happen that are on that piece of paper, the more you look the next time. And so, the more you think, 'right, I've had that before, I've just got this nagging feeling. That it could happen again on this job because there are similarities.’

**Interviewer:** Okay. Yeah. Thank you. So, you know, for the risk you just mentioned, how do you think of the controllability of it? The construability, I mean, whether you can do something to control it. Do you think the construability of the risk will, you know, affect your assessment, judgment on it?

**Interviewee:** We... what we tend to do, or what lots of companies do... I say to the estimator, 'price what's in front of you'. So, if we got a hole to dig this 10-meters deep, then as an estimator, we would price to dig that hole in normal sort of conditions. What we then do is as a contracting organisation who was on the tender, we would have planning engineers who work in the program. We have commercial people involved. We have procurement people involved. We have the senior directors and everybody within that team contributes to what we call the risk register. So, and the risk register through the tender period is a live document that anybody can put anything in, anything that they're worried about and so, we contribute the risks. Maybe get... I don't know, 100? 200? depending on how many people were involved and the complexity and number of have workshop to sit down and go through and sort of priorities which ones that are important ones and which ones it just sort of..., 'I know we've put that down cuz we're worried this but there's no economic or program issue if that risk occurs or doesn't'.

So, we then get the main risks and start to go through them, and we have quite lengthy debates if it's... because ultimately, risk is about opinions and experience and different people have got different opinions and different experiences and different levels within the company. They have a different ability about whether to accept risk or to prioritize the risk. So, the directors will have the ultimate say and they say, 'no, we're not going to take that risk'. And it doesn't matter whether you've priced for that risk or not.

As a company, you know, we could say there's a 50% probability of the wall falling down and make an allowance for 50% of the walls falling down. But if it does happen, it happens in its entirety rather than as a proportion of something that you have put in a risk register. So, we tend to look at the probability of an event and then the... what the outcome would be if it happened and go through, and we might come up with a number and that could be 15 or 20% of the overall cost that is applicable to the risks.

And then once we've put a number to them, we say, well, okay, what's the probability of that happening? There's an individual risk and then.... So just for argument’s sake, if we're dealing with drainage, there could be a risk about the water table and that's a risk on its own but then with the drainage as well, if we've got the water table, the material that were excavated out, might be water suck in and we can't use it, so we need to do something with it. So, they’re two separate risks, but if one happens, then the other one has to happen. So, some risks can have relationships with other risks and then some can be completely separate, nothing to do with anything. So, what you then have to look is say, well, if that one happened and that one happened, there's an overall probability, likelihood. And it's trying to... there's no real calculation for the probability that a risk will happen. The calculation only happens once we've said that there's a probability, there's a probability times a cost times X, Y, and Z, but it is the likelihood of a risk happening is down to people's opinions and experiences. It's quite a lengthy process, a lot of people involved.

**Interviewer:** So, this is what's your company normally do. So, how do you think of this process? Do you agree with this process?

**Interviewee:** It depends. Within companies, you've got different types of people in different experiences of people. And if you've got a good team working on a tender and it can be risky, it can be hard work, but because of the people that working on it, it goes okay. You can also have a job that it's a quite simple job, but if you haven't got the right mix and the right people working together, that process can become really, really, really hard as well.

So, the processes are there to protect the company and make sure we've done that, we've done that and people at various levels of... brought into what we're doing. It's the people involved within that process that can make it easier or harder.

**Interviewer:** Ok. So, you just mentioned some people will have different opinions, different assessment on the same risk. So, for your understanding, you think what makes people have different judgment?

**Interviewee:** Have you ever heard the saying that people who've got the t-shirt? It's a saying that we have in England and if somebody had a problem before, and it's happened. We say, they've got the t-shirt. So, they've got that. They've been in, they are wearing that experience and if people have experienced something before and it's caused them personal pain in the work life or it's gone particularly well, then they will always be influenced by that experience they've had, whether they try to ignore it or not.

That went really, really wrong and is a good contractor, you should learn the lessons from what's gone wrong and not do it again. But that person who it went wrong for is always got it within their mind. It's a problem. It can be that people can have those experiences quite early in their career, but they take them through as they get more senior and different people have different experiences throughout their career. So, you get a lot of different people arriving at different levels within the company with a lot of different experiences about different risks.

So, that sounds like a little bit of a big pile of you stir it up, but it's because of the experience and the knowledge of people that you get to have the debate. And if a lot of the team are assigning to the person who had the bad experience, 'look, we hear what you're saying, but this time we need to have learned, we cannot go through'. When we start the contract, we have a meeting on day one and we highlight that problem. And what we should then do is risk manage and manage the risks rather than just let things happen.

**Interviewer:** Yeah. Okay. Thank you. So, you know, in a team, how you finally make a consensus on a risk? How you judge, you know, whose idea, whose judgment is (better)...

**Interviewee:** The short answer is that it's the person who's most senior, who gets to make the decision, ultimately, because they're the ones that have to sign the tender. It's their responsibility as directors. So, they will make the decision, but if they've got a good team and what they should do is listen to the team and the team has a strong, healthy debate about each risk and each problem. So, it should be... the 99% of the time, the team comes to an agreement or a consensus about... he believes, she believes it's going to be terrible. He/ she believes it's going to be okay. Let's have a conversation. And then end up somewhere in the middle of two extremes. So, there is a debate, the people that make the decision are the senior or the team.

**Interviewer:** So, would you mind to, you know, give me an example because I like to know, for example, how people... from what perspective... people will support their ideas or judgment?

**Interviewee:** Yeah. What risk management about... is about, for me, it's about protecting the company from the effects of the things that go wrong. Cuz things do go wrong by accident by design, whatever.... things can go wrong. So, yeah, what we don't want to do is we don't want to put ourselves in the position where we know that we're accepting a risk that we can't control.

If we're working on a coastal job, or a job that runs over a river... the last few days, I don't know how... Do you live in Loughborough? I don't know what the weather has been like in Loughborough, but where I live, there's been some really, really, really torrential rain over the last few days. If you're working in an area, that's got a river close to the surface or whatever, we can look at normal weather and say, we're fine work then. But if it's... there's a sort of one in ten year or a one in 50 year or a one in 100-year event and the one in 100-year event is a storm just that bad that you should only get it in every hundred years. No, that's a theory because it doesn't just happen every hundred years, you could get two years... and that will be absolutely exceptional.

But we can't control things that happen during the one in 100-year events, because you could get half a mountain side washing down and you could... it could be almost catastrophic. So, if we can control something, then we will be prepared to take the risk. If we can't control it, or we can't influence it or help, then we don't want to take that risk because that's going to endanger the company through potentially the actions of weather... or all other people doing things that we can't control.

**Interviewer:** Yeah. Okay. So, I think you actually mentioned twice that your judgment, the start point of your judgment is to protect the company. So, you know, in detail, from what aspect you want to protect the company?

**Interviewee:** Reputation. If something goes wrong, in the construction industry and everybody hears about it. If there's a problem with this alarm slicker, whatever, then word gets around the industry. And what you don't want to do is you don't want to be in the position where people are thinking that your company takes risks, is works in unsafe ways. Because when you submit your tender and you say, we will do this, this, this, this, and this, in the back of their head, they're gonna be thinking that they might be saying that, but they actually do things differently because they take risks. So, there's the reputation of the company that we want to be seen to be a company that's say that manages its risks well, that looks after its people. Just lost my train of thought.... And so, yeah.

And then the second thing obviously is the cost because in the construction industry, we work on incredibly small profit margin between 2 and 3% generally, and it doesn't take very much for that profit margin to disappear. So, if we aren't on top of the risks, if we've accepted a risk that we can't control, then that 3% is a very, very small proportion of what we do, but it is the reason why we do it-- the company's got to make money. So, if 97% of what you do go wrong, it has a really catastrophic effect on the profit. And what we do is we look at it and think is... if you've got a job where there's been a bad occurrence or a risk that's happened that we haven't controlled, it generally takes somewhere around 10 jobs that performing normally to cover the lost profit or the losses that occur on the bad job. So, if you've got one bad job, then 10 good jobs. You then on a level where you're not making any profit. So, you don't want that bad job. You really, really do not want that bad job on a financial aspect because it ruins the performance of 10 good jobs.

**Interviewer:** Ok. Yeah. Thank you for sharing these. So, how do you think of your attitude to risk? Do you the person, you know who are willing to take more risk or you know, on the opposite? And how do you think this will affect your judgement on risk?

**Interviewee:** When... I said earlier on that the estimator, in reality, if we're digging a hole, should price to dig the hole, in normal type conditions. But as a person, if I had experience of things that can happen, then that experience will impact how I priced it. If I've had an experience where I've been digging a trench and the sides have fallen, and this has been a problem then within my normal pricing for doing that work, I'll put some supports in the trench to stop it being able to collapse and that is managing the risk of that collapse through processing it. And that sort of thing wouldn't necessarily go into the risk register that would be down to the individual estimator pricing it. Is he believing it should be done safely? So, you may get somebody who is more or less experienced, who would price to dig that trench in a different way without any support or would that... and they aren't necessarily wrong and the person who processes with the support isn't necessarily right. It's the blender of ... a sort of the experience of how you've worked with those things previously. So, we will price to do work safely.

**Interviewer:** Ok. Thank you. So, in highway project and in the, I mean, the risks you take, have you ever encountered a risk and you feel it's a little bit difficult, you know, to price it? Maybe, you know, the context of the project is dynamic or it's new, very new to you, or maybe there are some technical or design challenges? And so, can you take one as an example and describes how you finally price the risk, how you finally approach it?

**Interviewee:** When we're working on the highways network for Highways England as a client, they don't want any cubes in their roadworks. So, they want the traffic to be free flowing through without the traffic jams that backup and slow people down. And we through performance indicators, we can be fined financially if we slow the roadworks down by X percent, over a period of time. So, there's a risk that we can't really control because of a number of factors, that may be something and an event happening in an area where big audiences are gonna arrive, pop concert or whatever, and we can't control that when we're tender a year before hand or two years before hand. You don't know what's going to be happening when you're actually there. That's a risk that we can't control, but our experience tells us that by... if we suspend the works and if we stop working for short period of time and move the barriers back to give a little bit more space on the road, then we can generally manage that. That stuck sort of thing.

We won't... we can't take the risk on ground conditions unless we've got a lot of what we call site investigation. So, if we've got a road room's along 20 miles, and it is open down, where the line goes up and down what you want the road to go straight. So, we will cut and fill in materials. We can't take the risk on that ground material unless we've had somebody who's gone along and drill down the depth and the construction that we're going to do to see what materials there and how that material is going to behave. And what we can do is take the risk, instead of having a hole every two meters. So, we're absolutely certain what the ground is like. We might have them every hundred meters or every 200 meters, and then say, we will take the risk, that the ground is similar between those two boreholes. So, we won't take the overall risk, but we'll look at the quantum of the risk that we can take within that, if that makes sense.

**Interviewer:** Yeah. Yes. Yeah. Thank you. So, any knowledge or skills you think are useful, helpful in this process, risk pricing process?

**Interviewee:** It's the more you know about something and the more that you understand something and generally the better decisions that you can make. So, we should over time become better at our jobs, with more experience and with more knowledge, which is why you sort of have a trainee estimator, assistant estimator, estimator, senior estimator. So, you've got a hierarchy of different positions and as people get better, then they move up and as they move up, they get bigger projects, more responsible projects.

And people learn at different rates. They experience things at different rates. So, you can get people that get there very quickly, or you can get people quite a long time getting there. So, it's about how you accumulate and deal with your experiences and how much you learn from others as well. So, if we just, you know, I was talking earlier about, reputation. What we do is... the best thing to do is to learn lessons from what other people do wrong rather than making the mistakes yourself. 'Oh, that company did something wrong. This happened. Let's make sure that we don't do it'. We don't want the company to be the one that other people are learning from. So, yes, trying to learn across the business.

**Interviewer:** Ok. Thank you. So, I know you have many years of experience with highways projects. So, do you make any, you know, rules of thumb or guiding principles for yourself in risk pricing?

**Interviewee:** Not... not in individual risks. What... cuz I've been in a quite senior level estimating where I would get involved with the directors as well as working sort of at a lower level. And what people tend to do is they look up very broad comparisons. So, if we get involved in a scheme and it's very early in its inception, and there's a lot of details to be. Then people will say, we'd need a risk allowance of 10%. Or if we've got a scheme, that's quite well-developed and a lot of the details are there and we know how the risks are gonna be a portion then, right, we'll say that risks two and a half percent, or it feels like a two and a half percent risk job. And that's what a rule of thumb.

But there's probably not a lot of science behind it. It's just, yeah, it's just a rule of thumb, but people have always said 2.5%, 5%, 10%, that sort of thing. So yeah, there are some.

**Interviewer:** Okay, thank you. So, how do you think of the idea that maybe one day, you know, the role of estimator, they can be replaced by some algorithm or computer softwares? I mean, maybe they can do risk pricing automatically. How do you think of this?

**Interviewee:** I like the question. I do like the question because I think the estimating could be done by algorithm to probably about 95% certainty. Whether you then get the... An example, if you put in a curbing and it's in the middle of a field with a new road that you're building. Then that's fine. And that's a way of doing it. If you're then in a different, in an urban environment where you work in and there's traffic there and you've got pedestrians there. It's whether the algorithms will pick up the difference between different work locations. So, I say yes, as long as the algorithm has the ability to work. Or if it's a simple algorithm that it's occurred, it's the answer is A and then no. It won't be any good, but if the algorithm is good and it can take in a number of factors, then that answer would be better.

Probably then still needs a human brain at the top to look at it and just say... cuz when I've managed people I've always gone through and done a double check on their price. So, you would still need to do that if it was a... if it was generated by algorithms, because nothing's fair safe. So, yeah.

**Interviewer:** So, how do you think of, you know, the difference between the risk pricing done by computer software and those done by human beings?

**Interviewee:** You might, you might get a better answer from a computer because the computer won't have opinions. It will be working on logic, on fact, on what it's been given to work with. It won't say, 'well, I don't like that, or, but I do like' that type pulling. It might... yeah, it's interesting way of looking at it because it takes the intuitive, emotional side out of it.

**Interviewer:** So, do you mean, you know, it's more... it's better to be objective or subjective, you know, in risk pricing?

**Interviewee:** Everybody tries to be objective, but it's having the ability to not involve your emotions and your feelings that then become subjective. So, it's better if you're objective in the way that you're doing it. And particularly if people have different opinions, it then becomes ever more subjective. Because while they're being subjective, they probably believe that their action being objective from their point of view.

**Interviewer:** Ok. Thank you. So, how do you think the idea, you know, risk pricing, it's always, you know, financial application on risk. I mean when you're pricing risk, what perspective do you thinking from? Do you only think from the financial perspective or...?

**Interviewee:** Yeah... it, ultimately, we build in something to help with the environment, make life better for people, that sort of thing. But a company in business generally to make money. We will look at the reputational side of risk. We will look up the time side risk, but I think the long in short of that is that if there's a reputational risk that happens and we do something wrong, it might... that reputation might not affect us financially on that job, but if we don't get the next job and the next job because of something that we've done wrong, then that will affect us financially. Also, if we say that we're gonna finish a job by the end of the year, and we don't finish by the end of the year, then we might have to pay damages to the client for finishing late, or it gets publicized that we've finished late. So, everything comes back to money in the end. There are two different headings but all impacts on the company, on their ability to make money and grow and be a good company.

**Interviewer:** Yeah. And it seems to be, you know, it's a principle for your company, so, for yourself personally, do you agree in these or, you know, you have some other thoughts?

**Interviewee:** Yeah. The company that I'm working for a minute has strong belief in its behaviors and on that it should behave well for its clients, for its people. And if you are working for a company that's like that and the decisions that you're making, the right decisions, whether you're thinking about that decision with pound notes against it. If you take a human situation, and you're managing somebody with very, very little cost, if you help them, if you mentor them, if you give them training, that's doing the right thing. What it can do is it can motivate that person to stay with the company and to do better for the company. And if that person left, because you aren't doing things right, then we probably have to pay to recruit somebody, we then have to pay to train them. So, if it's... when you're doing the right things, things tend to go right financially, overall.
