# Supplementary material for: Critical factors influencing cost estimators’ judgements on cost contingencies in highway construction projects: An empirical study in the UK
Source: PLoS One. 2024 Dec 16;19(12):e0314665. doi: 10.1371/journal.pone.0314665 (PMC11649144; doi:10.1371/journal.pone.0314665)
Supplement: S2 File — (ZIP) [file pone.0314665.s002.zip › Transcription (Interview I).docx]

**Interview I-Meeting Recording**

**Interviewer:** Okay, can you explain how you make the judgements on cost risk allowances allocated in the tender prices?

**Interviewee:** So, about risk, we will look at... is it something we know about historically and what problems we've had with it or it was... and then, so that will influence our thinking on risk. And also we'll look at where it is because you've not only got the risk of, you know, the quality risk, so the product we're trying to produce, but you've got the risk of how easy is it to get to it, the location, can be a risk, transport. If it's in the center of London, it's very difficult to, for the logistics. If it's in the middle of you know, the country, the other risks we have, there is environment and ecology. So there's... it swings quite dramatically.

So if we're looking, say in the country we're looking at... ok, so it's far from the main road. So the transport is a risk, particularly. So with concrete, are you going to get sufficient delivery quick enough? Then I have all the environmental and ecological issues being dealt with. We need to make sure that in our planning and in our pricing, that we've got, you know, we've taken care of those issues.

Some of which will have long notice periods. So we would work with the planners to say, 'right, you're going to have to do this, but to do this operation, you may need to give nine months’ notice or six months’ notice'. We also need with the planners to understand the ecological calendars so that if... there is species need moving. There's only... there's all different times of the year that you can do these things and we have to understand that, okay. That is all parts of our process. Well, then we, and that's the sort of the delivery risk that we're looking at then of course, as estimators, we're interested in the pricing risk.

Which currently is something nobody can predict because construction materials prices are going up exponentially at the moment because obviously, I suppose it's a post pandemic. Boom. Everybody wants materials and it may be that some of the... some materials production was scaled back when there was no demand. Now the demand has gone completely mad. They can charge whatever they want to challenge.

And it's the availability. That's another risk we have to think of because if it's... if it's not available there's a very good chance the price will go up. So there are the key things we look at is the delivery risk and what can prevents us from delivering and the price risk and the escalation risk.

**Interviewer:** Okay. Thank you. So, you know can you pick one risk you know, in contractor's risks and talk about, you know, all the things you will think about while you make assessment, on it. I mean, the it's probability of occurrence and its impact. What things you were thinking about?

**Interviewee:** Well, particularly from our point of view and, and we're being asked to fixed prices earlier in the process. So we haven't got a complete design. So for that point of view, for most of what we do, the biggest risk is ground conditions. So we don't have a complete design. We don't have a complete geotechnical investigation. So we then have to make assumptions on the risk of the material in the ground or the ground not being strong enough to support what we will constructing. And we will look at that and say, right... well, we have, we know that the ground historically, and we can go onto the geological society website and we can find certain or whole data, which will tell us what the ground is, but we'll also, we'll be saying 'right, well, some of the data tells us that the soils and the ground are this strength and this nature. But we will know from experience. So yes, but we know historically in that area we encountered a problem'. So we will then say, right, the chances of the ground not being satisfactory is 30%. So we will say about 30% of the material may be unsuitable. Therefore the risk is pretty high that we will have to import material. And that is obviously more expensive and we will put those risks into a risk register. So we will say that our feeling, because a lot of this can only be done on the experience and your own feelings. There are computer models that generate a risk profile, but they're only as good as the information you put into them and so you say, right, we think that there's a 30% chance that we will need to import an extra 100,000 tons. So, we price in 100,000 tons multiplied by 30%. And then we think, well, what's the likelihood of that occurring. So, then we have a, like a best case, worst case and an intermediate case. And that's how we do the calculation.

**Interviewer:** Okay. Okay. Thank you. It seems like that's what your company normally do. So, is that the same as how you do this?

**Interviewee:** Yeah, yeah. We tend to do this. Although we use, there is a standard spreadsheet that we use, which has some pre-calculated formula in it to help you. But again, it's, it's really, each job is slightly different due to location due to where it, due to ground conditions. And you just really have to take all those factors into account and use your experience.

We also have things like we have in probably the way as we... there are there... within various organizations, there are various stages of design so that like you have RIBA, the architect spec, or building spec, where for each of the stages, that is the maturity of the design with rail work, you have GRIP, which is the guidance for rail infrastructure projects. Each of the designs and execution stages have a number. And depending on what stage you are. Is the level of risk. And then with that, we tend to use another thing we... it's another tool we have, we use the [tool 1] [took 2], which calculates optimism bias. So that if you're... if you have a very immature design, then you might say, well that design's going to change. Therefore, my pricing going to change. It is likely to change looking at the historic data. I may have to add 35% optimism bias. If the design is more mature, then you may add 15% and it's all laid out in tables. When you come to actual, you know, you've got a mature design, you've priced the full design. Then you'll figure will be, say 5%, which is your risk, which is what we've been talking about, how we assess the risk. But on average, for a major construction project, you will be looking at around about between 3% and 5% risk allowance and mostly. So, so mostly we would go between 3% and 5% on our own as a risk allowance within a major project pricing.

**Interviewer:** So, you know, who did decide the rate for the likelihood the impact?

**Interviewee:** It's usually done at one of the you know, with, with a group of people, people who have knowledge of the project, people they're doing the pricing, the planning, people doing the engineering and the methodology. And we will have a group discussion on this. And I may say, well, I think it's likely to be 20% chance that this risk will be realized and somebody else might have a different view. But at that discussion, we will come to a conclusion. So, we will have a risk review. So, what we'd say, all of our estimates, we have a SharePoint site, so everybody can feed information and easily. And then we everybody puts into the schedule.

So, somebody thinks of a risk, it goes on, however, large or small that risk may be. And then when we have a review of that documents as a group, and it won't be a big group, maybe six people we will go through and say, well, that risk is so small. We'll dismiss that or that risk could be a real problem, therefore we need to, and we'll, we'll, we'll refine it. So that when we present our tender, we maybe have 20 items, you know, 10 of which will be big numbers. And then a likelihood... you have to remember as well as some of these risks within the contract will be client risks. And then his ... the contract may say that if you find unforeseen ground conditions, the client takes that risk. That's fine. We will put it and we will bring it to his attention. So, the client's attention. So you do realize that this is your risk and this is the chance, but that doesn't go into our pricing. We just bring it to the client's attention. But if it's our risk, then we have to put a figure against it because if the risk is realized, we're going to pay for it.

**Interviewer:** Yes. So, you know for your understanding why people in the same group, they will have different opinions, different judgments on the same risk?

**Interviewee:**  I think people who've had different experiences in their career and also some people look at it from a purely a productivity or, you know, delivery basis. Whereas somebody like me, I look at it how much it's going to cost and you know, at the end of it, we are all in business to make money. If we do not make money, we don't survive. That's the... that's the reality.

Also the planners the guys who do the programming, they will have a view on the program risk, will be slightly different to the commercial risk... will have an impact commercially, of course, everything does. But there will be... most of the opinions actually will be fairly aligned. There will be slight variances in the opinions, but it's always good to have challenge anyway.

**Interviewer:** Okay. Okay. Thank you. So, for yourself, how do you think of, you know your personal attitude to risk? Are you the person who are more willing to take risks or, you know, you're more conservative and do you think this will affect your judgment on risks?

**Interviewee:** I think I am very risk averse. Yes, very conservative when it comes to risk and I will in my pricing, I will state what I believe to be the risk and put a figure to that risk. They usually tell me that I'm too pessimistic, that I've put too much in, but I'd rather bring it to somebody's attention and let those higher up than me make those decisions. But my view is I, my job is to analyze it, evaluate it, and then present the information. What others do with that information is entirely up to them. But I am... yeah, I would say that I am very conservative when it comes to risk dances. Maybe because we might be as on the delivery side, most of those risks have been realized and I've had to sort them out.

**Interviewer:** So and you know, you just mentioned the ground conditions. It's you think, the risk mostly happened and that you pay attention to. So how do you think of , you know, its controllability? The controllability, I mean, whether you can do something to control it. How do you think of the contribute your that risk? And do you think, you know, the controllability of the risk will affect your judgment?

**Interviewee:** Definitely does. I mean, how, how we can deal with the risk and how we can mitigate it is very important as to how we judge it. I mean, if we have a ground condition where like a cohesive soil isn't suitable, it's too wet. We can do things to it, which so we can think about, well, maybe we have to stabilize 25% of this particular area. So, we can price then for mixing lime and cement where the cohesive soil, which is a lot less expensive and a much more efficient solution than digging it out and trying to replace it. So, we will look at those sorts of solutions at the same time. So it's... so that the whole thing is quite a holistic exercise.

**Interviewer:** Okay, thank you. Thank you. And sorry, I think of the question, which I just forgot. I'm sorry, what you just mentioned that you will have a discussion group and so, I'm curious, you know, from what perspective people will you know, to support, to argue for their own judgment?

**Interviewee:** People will, they will put a good case but again, we will go and everybody will see everybody's points of view. There will be a senior person in the room who will make the final decision based on the evidence presented. Cuz obviously we can't... We have to, we have to come out of that discussion with a decision and as time goes on, that decision may change.

As we get closer to the tenders, people might think, well, actually we have now had... if we have more information. It might be a late issue of the geotechnical report and it might say, everything's fine. So we will remove that risk from the risk register, because we are now satisfied that we have all the information we need. And that's how the... so the whole thing is an evolution. Almost up to within sort of three or four days of a tender being submitted, the information is evolving all the time.

**Interviewer:** So, you just mentioned that, you know, you said experiences are the thing, which mainly, you know, make people have different judgment. So, for yourself, you think, you know what experiments have mostly ...have the impact on your risk pricing, the way you do risk pricing now?

**Interviewee:** The way I do risk pricing. The thing that influences me most is the fact that I've been on delivery side. I know what are the physical and actual risks that we can encounter on site and you know, I've been on quite a few different types of projects.

I mean like one risk and again, it's becoming more important now is weather. If it's high winds, you can't lift use cranes. So, you have to think, well, the area I'm in, is it, is it... what is the providing wind direction? How often... we can look at weather records and say, well, how often does the wind exceed a certain speed here? And we can say, right, we are going to have 20% downtime on drainage because there'll be 20% of the time. There'll be one day a week where we cannot use a crane and then the program will reflect that. So, if the program reflects it, then we wouldn't put a price against it in the risk register because the program and how we price the time-related costs would reflect that. So, we would, it would extend, the time related costs would extend slightly. We wouldn't need to... the danger is you're putting... if you're allowing for it in your pricing and programming, then you wouldn't price it as a risk. Cuz you'd be pricing it twice. So you have to, then that's another thing we have to take into account that we don't price more than once, otherwise we're not successful in the tender.

**Interviewer:** So, you know, besides experience., do you have any, do you ever done any courses or training and ... or say it in another way... So, do you think, you know, the risk pricing work can be taught or learned? How do you think of that?

**Interviewee:** I think it can, if you're... if you're doing a purely numerical analysis, then I think it can be but I still think in most of what we do, it is a function of... it's like anything. It's, it's a function of training and experience. So it's.... and you can learn of other people, but... and, and I still do. I mean I, if I don't, if I'm unsure, I think, oh, that persons dealt with that. I will go and seek good advice. Which, which again, these are the other tools that we have. There are people in our business who know more about things than I do.

**Interviewer:** Ok. So, so in your personal experience in the highway projects, have you ever encountered a risk, which you feel, you know, it's a little bit difficult for you to price the allowance for it? It may, because you know, the context is dynamic, complex, or maybe there are some design challenging, something like this. Can you take one risk as an example and tell me how you finally approach it?

**Interviewee:** I suppose. Some of the structures that we've had to deal with. We've looked at the design of them and how they're made. We look at the other constructability risks. Actually, how do we actually lift this? Or how do we transport this huge lump of steel and how do we lift it and then how do we make it stable, in a temporary condition? Because quite often the bridge deck was absolutely fine when it's all together. But while you're actually construction, you'd have to think about your, what extra support does it need and it's temporary condition.

And we've had one recently where the structure was very eccentric and we had to earn the pricing, but we looked well that... if that stays... in a certain stage of it, this thing is going to topple off and fall on the ground. Because it's... the center of gravity was outside the line of the beam, in a temporary condition. So, we basically, we allowed for temporary propping and there's things like that we have to take into account. We look at it, we looked... do a quick analysis . We get the temporary works department to analyze it for us -- what do we need to do, well, we need to temporarily prop or temporarily brace something. Then we will, might get a design and we will allow for that in the pricing. So it's a risk that we've noted and we've dealt with.

**Interviewer:** Okay. Okay. Thank you. So, you know, in this experience, what skills or knowledge you think are most helpful for you?

**Interviewee:** For me, my practical knowledge. The fact that I've actually delivered projects, but I haven't been an estimator all my working life. I've only been doing estimating for the last five years. So it's... and that's how I tend to look at things is... that it's the practical. Because, because you've actually dealt with it physically. It gives you a much better insight into how to deal with it when you first see it on, on a drawing.

**Interviewer:** Thank you. So, you know, from your years of experience, in highway projects in risk pricing, do you have any like rule of thumb or sort of guiding principles in risk pricing?

**Interviewee:** Guiding principle really is, well, first look at it and think, well, what could go wrong? What's the worst that could happen. How can we... Look at the program, the logistics, the... you know, how we build it to overcome those issues? And then if there are any residual issues that we can't resolve at the stage we're pricing of it. Do we, do we price and put some money in to cover, you know, if something goes wrong or we need to do extra work? But basically, the first, first rule of thumb is really what, what can go wrong? I know it sounds very pessimistic, but we're not, but we're just, you know, we've got to make sure we get it right.

**Interviewer:** Okay. Thank you. So, you know, how do you think of the idea that maybe one day, you know, the estimator, the role of estimator, it can be replaced by some algorithms or computer software? I mean, maybe they can do risk pricing automatically?

**Interviewee:** I think that's a long way off. We have, we do have software that will run analysis of risk, but again, it's all, depending on who's putting the data in. There are lots of bits of software that and, and lots of analyses that can be done. But there's a lot of answers from these analyses that you can decide what the... you want the answer to be. And they... the data gets that... gets input will give you the answer you want, not necessarily the answer you need. So, I personally, I'm a little suspicious. I think we're a very long way off having software that we can trust 100% to do risk analysis.

**Interviewer:** Okay. So you know, compared to the software, what do you think if the advantages of, you know, human beings to do the risk pricing?

**Interviewee:**  Purely the fact that we can... it's almost the fact that the human beings can think illogically. So we can think outside the box, whereas the machine will always think logical. Well, I think that's an advantage to us.

**Interviewer:** Okay. Thank you. So you know, you think the risk pricing process you think it's better to be subjective or objective?

**Interviewee:** I think... It needs to be objective to achieve that otherwise you'll just end up getting nowhere. You won't... you'll end up with too much risk in there. I think we need to think about what do we need to achieve by analyzing the risk. We need to get the job done. We need to get the price correct. So, we always need to look at it objectively, but with, you know, making sure we've done a proper analysis of each risk.

**Interviewer:** So, in reality, are you trying to do the risk pricing job objectively?

**Interviewee:** Yeah. I will say so.

**Interviewer:** So do you think there are any factors that maybe you're unaware of it and they have impact on your judgment?

**Interviewee:** So difficult one. I think the, the thing is we look at these various risks and a lot of it is to do with how, how we judge our risk is based on how much data we actually have; how much information do we have on that risk. I can look at a design and say, yes, that's perfect design. There won't be any problems with it. Or I can look at a designer design and think, well, this is a very young design. It needs a lot more work. And therefore, there's a lot of unanswered questions, therefore I need, you know, to allow for more risk. But the more information we have then the less the risk becomes.

**Interviewer:** Okay. Thank you. So I think you talking a little about this question at the beginning of the interview. How do you think of the idea, you know, risk pricing, it's always the financial application on a risk or, sorry, let me say it another way. I mean, when you pricing risks, well you only think from the financial perspective?

**Interviewee:** Yes, I do. I, I, I, obviously we think about the likelihood of it happening. There are, you know, and we look at... we do look at things like safety risks, but... but if you can... if you... but again, that's, you know, it all part of the process in formulating how you construct the job and how you overcome, you know certain risks, but you then... at the end of it, we have to do it safely. So safety risk is part of the methodology. But the... But, once we get to a point, then we've worked out the method. We've worked out what we need to do. We've analyzed where the risks are and what we need to allow for, if those risks realized.

So yeah, from my perspective, it's... I want to know what it's going to cost, but there is lots of factors before that. Can we do it safely? Can we do it that way without damaging the environment? Can we do it in a sustainable way? But this will be part of how we build up our total pricing.

So there will be a team of engineers looking at the methodology pre-tender. And they will say, right, well, this is how we're going to do it. And then as the estimator, we will say, right, if that's how you're going to do it and that's the safe way to do it and the best way, the most efficient way of doing it. That's how we will price it. So we won't just price it and saying, well, there's a list... there's a, there's a shopping list. We'll put some prices to it. We want to know how it's going to be done because that'll give us a more accurate price.

**Interviewer:** Okay. So it seems like, you know, how your company doing. So do you agree with this process or you think, you know, there something can be, you know, improve?

**Interviewee:** I think any system can be improved, but I think the way we do it now is as good as anybody else's way. We're always trying to make it better and to refine it. But what we have at the moment works and it's proved to be successful. And every time we do, when we think of, we're thinking of better ways of doing things better ways of analyzing the risk.
