# Supplementary material for: Critical factors influencing cost estimators’ judgements on cost contingencies in highway construction projects: An empirical study in the UK
Source: PLoS One. 2024 Dec 16;19(12):e0314665. doi: 10.1371/journal.pone.0314665 (PMC11649144; doi:10.1371/journal.pone.0314665)
Supplement: S2 File — (ZIP) [file pone.0314665.s002.zip › Transcription (Interview J).docx]

**Interview J-Meeting Recording**

**Interviewer:** Yeah. Okay. So, I'm quite curious that, how you become an estimator, you know, do you plan to do this, or it just happened?

**Interviewee:** No, I was working on site as an engineer building... I was actually in the steelworks in [City] at the time building things and the training, the company was in, they put you in each of the different departments for six months to a year. And I came off site, went into an estimating department. I quite enjoyed it. They thought I was quite good at it. And to be honest, I then just, that's where my career went, and I just stayed in estimating and working for different companies. So, I've worked for quite a few different companies over the years, but mainly in estimating. So, mostly estimators, have worked on site, and then come into the office to do either planning or estimating and get in that way.

There are some that just come straight. We've got a guy who's working for us, who's actually comes straight into estimator, and he's got no site experience. So, we'll see how that works over the next few years, you know, whether he needs to go out and do some work on site. But so that was my past. Start on site and then came into the office.

**Interviewer:** Okay. Thank you. So, do you have any courses or trainings, which related to risk pricing?

**Interviewee:** It's such a long time ago. Over the years, I've looked at risk for different companies and they're all look at them in different ways. One time, the big thing that everybody was using was a thing called Monte-Carlo. It's a ...you know of it, that seems to have died a death, and we don't use, those types of models anymore.

We tend to look at risk now as value and probability, and nothing too much more complicated than that. You know, there's a risk that is my… cost a hundred thousand pounds, and the probability is… it's 30%. So, you put 30,000 pounds and, you know, it's a very simple way of doing. Other times people look at a job and they'll say, well, on this type of work, this sort of client, we would put a percentage on it. It may be 2%, 2.5%.

So, there are lots of different ways of looking at risk. But I personally think the right way to look at risk is what is the risk, what's its value, what do I think the chances are of it happening and say, it's just a value of times of percentage. If you present it in that way, that people can, just go set reflect on it and say, well, you know, I think there's less chance, or I think it might cost less or it might cost more. So, it's an easy way to review risks.

I mean, the other thing I should say is, for me, there are two types of risk. There are physical risks and there is commercial risk. And physical risks of health and safety and environment, they don't appear in my risk registers at all because they are covered as part of the price, we can't ever look at a physical risk and say, well, there's a chance that might... man might get injured. There's a... I'll put a 50% probably. No. You price it. So, there is no chance that man might get injured. So, it's not, it doesn't appear in the risk at all. And the same with environmental risk, you know, there's a risk we might pollute the water costs. No, there isn't, we've allowed sufficient money that there's no chance we're going to pollute our water. So, the only reside really talk about in the risk register are commercial risks, not physical risks at all. I mean, is that what you wanted to talk about? Physical risks or commercial risks?

**Interviewer:** Actually, both, but more prone to the commercial ones. Yeah, yeah, yeah, yeah. About, you know what you just talked about; I have many questions about that. Yeah. So would you mind, you know, to firstly take one contractor's risk as the example, and talk about, you know, all the things you will think about while you make the judgment, which you just mentioned, the cost for it and the probability. Yeah. What things you were thinking about?

**Interviewee:**  One risk, which is very, very typically put onto the contractor is unforeseen ground conditions. So, when you dig out what you'll come across. So, the process that I always go through in trying to assess what that risk is is first of all, what information do I have? Do I have lots of soil information, boreholes, trial pits? So, I know what the material is we're digging in. And if I do, then the risk is quite low because I know what it is, and I can price to deal with that material. So, I know what it is... you know, what level we'll go to, to find a foundation. I know whether the slopes or stand up or whether it needs... So, if I've got lots of information, I can allow the cost based on that information.

If at the other end, I have no information whatsoever, the first thing I would do is say, can I move that risk from my risk onto the client? Can I say 'because you're not giving me any information, that's not fair that you're asking me to take that risk. You know, that would be yours'. Mostly they say, 'no, it's in the conditions, seeing the contract, it's your risk'. So, then what you have to do sit down and say, well, what are we doing in this ground has a risk and one thing might be supporting the ground. And if we think there's a chance that the ground needs supporting, we would have to allow for piling, you know, a cofferdam. And then you start saying, well, we may or may not need that coffer down. And that's when it becomes a risk, it's not a... we definitely need it, it's in the price. It's we may or may not need it. So, it's a risk. So, then what I will do is I would price all the cofferdam and say that cofferdam, you know, when it's piled and whatever, my cost is 50,000 pounds, then I look at it and I say, 'first point would be well, that's a coin. It's a 50, 50 chance we may or may not'. So, the starting point is probably 50 50. Then you start to think a bit more about it and you say, well, although we don't have any good ground information here, locally, we know it's in clay or it's in sand or it's... you know, so we got so many ideas of where it is. And so, we might say, 'well, we can downgrade it from a 50 50, it might be a 25% risk. And we all agree that yeah, we'll put, it's a 50,000-pound cofferdam, but we'll put 25% of the cost'.

It says... it for me, risk is understanding the risk, what information do you have and then based on the understanding and the information, value it and say what the probability is. And that... I do that for all risks really but say typically ground conditions would be one that would get onto is.

Another standard risk is weather. For us the job, we’ve come up with a program and there's a chance that there's a... there's a chance of weather, it will affect us. So, then you look at the program. You say, 'when's the worst time for bad weather?' Worst time is general in the UK... It's not usually at the... coming into the winter. It's usually coming out of the winter. Autumns in the UK, generally, very wet, lots of rain. Say, what operations are we doing in that time? And the worst one you can be doing is earthworks, doing bulk earthworks. If you're doing earthworks in the spring, then you're going to get delayed. So, then we look at that and we say, well, because of the operation we're doing, we're doing earthworks in the spring. Let's say we're gonna lose two weeks. Well, it'll be two weeks we'll lose. How much does it cost to run the site for a week? Maybe the site and all the earthworks team would cost us 20,000 pounds. So, two weeks, times 20 that's, 40,000 pounds. And you could just say, well, it's gonna happen, put 40,000 pounds, and put it in. You might actually say, well, it's a chance might not happen. So, then you're looking at its 4,000 pounds in the risk register on what probability you putting on it. So yeah, you might say, well, again, start with 50 50, but chances are, it's going to be higher than 50%. So, you might take a 75%, a percentage on it.

So, it's always a balanced what goes into the risk register, what actually goes into the actual price straight. I had a conversation the other day, and we were talking about risks, and somebody said, put it in the risk register and put it a hundred percent. And I said, if it's a hundred percent, it's a certainty. It's not a risk. It's a certainty. So, that’s not going in the risk register. It's going into the price as a price item. So, it's just a balancing hope. That's an item to look at risks like that. So, yeah.

**Interviewer:** Okay. Thank you. Thank you for sharing these. And you just mentioned that, you know, the subject, 'we', so, do mean that you do this in a team or, you know, you do this personally?

**Interviewee:** Yeah. Well, in an ideal world, in an ideal world, you'd have a blank risk register. You'd get all the team together. You'd sit down and you'd say, 'let's come up with some risks' and you know, whatever methodology, sticking things on walls or flip charts, whatever. And you'd put something together and as a team, you'd pull it all together. That's an ideal world. It very rarely happens.

What happens normally is the estimator has a blank risk register, and as he's going through pricing the work. It comes across risks. Some of them are always there, weather, ground, you know, that they're always there. They may be a big risk or a small risk, but they're always there. So, the generally what happens is the estimator pulls the risk register together. He has his view of cost and really the estimator is the best person to come up with costs cuz that's his job. So, he'll come up with cost, in obvious, first stab a probability and it maybe-- I have no idea, so, it's 50, 50, you know, all way down. And then that risk register is reviewed by the team. That'll be viewed by the delivery team, the people who are going to build it. It will be reviewed by the commercial team and the management as well. They'll review it. They might add to it, say, the estimator has missed some risks that other people think are big risks. They might decide, well, some of these risks, I think, aren't really risk, we'll remove them. And some of them might say, well, I think your probability is a bit higher or a bit low.

So, it's generally the estimator puts it together, but then it's reviewed by a team. It's in my experience, that's the way it normally happens at the moment. I mean, ideally, it would be a team effort, but the teams are too busy really.

**Interviewer:** So, you know, how do you think, why people, they will have different judgment on the same risk?

**Interviewee:**  People's judgements of risk of based on their own personal experience. So, if someone has had a problem on a site with ground conditions, say, they are very focused on, you've not got enough money in for ground risk. If, weather has been a problem to somebody, you know, they've had a crane that couldn't move because of the wind was too strong or they've had a job where all the earthworks have been held up for weeks on end and so they, you know, cost a lot of money. They'll be very focused on ground condition risks.

So, everyone brings their own experience to it. And what you have to do is make a judgment call based on everyone's experience. If you always take the worst case, you'll end up with a massive risk register and you'll never win any work. If you always say that the sun shines the case, then you'll not have enough in. So, you have to try and balance it and, and apply people's experience to that particular job.

So, say somebody had really bad experience of ground conditioning, wet weather, and they were in clay. In the UK, clay, just you... you just stop, you don't work, if there's problem in clay. But the job you're looking at is a sandy ground. You might say, well, it doesn't apply because this ground will dry out really quickly. And so instead of you saying it's a four-week delay, you know, we will to get away with only a week's delay on this because it'll drain. So, you take people's personal experience, and the estimator has got his experience. So, that's why it needs reviewing by other people. Because you need their experience as well, to look at a risk and to say, yeah, you know, 50, 50, but I don't think it will be as bad as that. We agree. It's only a 25% risk. So, to me, it's... you start with a list, and you use people's experience to, to try and get it to near to a number that everybody's happy with.

**Interviewer:** Okay. So, do you mean that, you know, the people, for example, in the financial team, when they have some opinions, different opinions, will they discuss with you or, you know, because they are in the high level so they can, you know, just change it?

**Interviewee:** Well, they can, they could do, if they're my boss and my boss says do it differently. Then, you know, how I work if… but it's my experience that doesn't very... that doesn't happen very much. Certainly not these days. He may have done 20 years ago when the industry was a lot more authoritarian than it is now, but nowadays people want your judgment. They want your ideas. They may disagree and generally it's a compromise between someone who thinks this risk is really, really big, someone who thinks this risk is really small. There's a compromise somewhere in between.

And that... risk is not a real number. It's a 'maybe number'. So, it is a range, somewhere between the worst case and the best case. So, and we're, we're always trying to find that position and it sometimes we've towards the worst case, sometimes it's towards the best case, but it has to be a team decision. But at the end of the day, if the managing director says, 'I've heard all what you say, but we're going to put a hundred thousand pounds in there'. That's his job and you know, if he wants to do it, he'll do it. I'll argue with him that it shouldn't be. But if at the end of the day says it is, you know, and that's what we put in, but that's just the way of business, isn't it? You know, somebody has to be in charge at the end.

**Interviewer:** Okay. So, for you, from what perspective you will argue for your own judgment, your own opinion?

**Interviewee:** If I think a risk is a real risk and it's a big risk, I will argue that we should put some money in for that risk. If I think somebody else has come up with a risk that I don't think is a big risk, I will argue, we shouldn't put money in. So, it's I said to you before, it was all about personal judgment. It's all about personal history, and that's why it has to be a balance between everyone, because I'm not always right. Probably wrong, more than I'm right. So, I will argue my case. I'll say what I think is the right amount of risk to put in, but that's not just my risk register. It's the project risk registers. It's the team's risk register.

So, the thing I will get annoyed at people is if they put risks in which aren't risks. So, somebody said, this is a hundred percent risk. No, it's not a risk. It's a certainty. It goes in the price, or it's somebody starts talking about a health and safety risk. I'll say no. If you've got a risk on health and safety that should be addressed in the price. We should have allowed enough money to do this job safely. We shouldn't ever have a health and safety percentage. You know that there's a 10% chance we might harm someone, no chance. You know, there's a no chance that we harm someone, or we don't put the price in at all.

So, yeah, I'll argue, whichever I think is... my... you know, in my experience, if it's a big risk, I'll argue for it, if I experiences it's only a small risk, I'll argue that as well. But it's a balance then between everyone in the team, you know, if enough people think it's a big risk, it's a big risk.

**Interviewer:** Okay. Okay. Thank you. So, for you, how do you think of yourself? You think you are, you know, you're more risk... conservative in risk or you are the prison who would like to take more risk, and do you think this will affect your judgment on risk?

**Interviewee:** It does. Yes. And I, because of my years of experience. I am very, very conservative. I'm very pessimistic. I've seen things go wrong too many times. Yeah, you know, so many jobs have had problems with earthworks, so many jobs that had problems with weather, you know. So, just because of my experience, I am very conservative.

So, I will say that that risk is 100,000 pounds and that risk could take two weeks or whatever, and that's why it needs review. That's why you need then someone who is less conservative to actually review it and actually to mitigate some of that down, you know, to say, 'well, all right, you might cost 100,000 pounds but it's unlikely to'. I'm gonna knock your risk percentage from 50 down to 25.

Generally speaking, estimators, are two things that pessimistic, they're conservative, because they've seen lots of things go wrong, but they want to win the job. So, they don't like other people add in money into their jobs. So, if somebody else comes along and says, 'oh, I think we should put 50,000 pounds worth of risk in for this'. And I don't think that needs to be in, I will argue it, but generally speaking estimators are quite conservative because we've done so many jobs. That so many of them have had a problem with earthworks or weather or whatever. So, I'd say I am, and the majority of estimators are conservative in the way they do things because you get caught. If you're not conservative, you get caught out too quickly. People go back there and say, there isn't enough money in this job to build it. So, that's why we tend to be conservative.

**Interviewer:** Okay. Okay. Thank you for sharing. Yeah. So, you know, in your experience in highway projects, have you ever encountered a contractor's risk, which you feel it's... for you, it's a little bit difficult to price the allowances for it? Can you take one, an example and, talk about, you know, how you finally approach it?

**Interviewee:** Right. There's one that's happening right now and that is after COVID, we are in a position where in the UK prices are rocketing. They are really going up. We can't get materials without paying lots of money for them. Subcontractors are charging more. A lot of us, contracts are fixed price. So, we have to predict where the prices are going over the next year or two years, depending on how the... How long the project is.

I had a conversation yesterday. The conversation is I cannot predict that I'm not... I don't have the information to predict that sort of crystal ball gazing. So, what we're doing in that instance, we're going to get together our commercial leads, who are placing orders for subcontractors. We're getting our buyers involved who are placing orders for materials. We're talking to suppliers to get their ideas. of how much prices are going to go up over the next few months and years. And we're going to actually tabulate all that to come up with a value.

So, I'm not... as the estimator, I'm not doing that because I don't place orders. I don't buy materials. So, I've got the people who do place orders and do buy materials to actually... They're the best people to come up with the numbers. Well, then review those numbers. We might make a judgment that we're gonna add a bit more or a bit less in, but that's how we'll do. It's finding the right person to make that judgment. And you start all with the estimator. And in this instance with crystal ball gazing, what materials prices are going to do over the next two years. And that's not something that. I'm really qualified to do, to be honest. Others are more qualified to do it and I'm going to let them do it for me.

**Interviewer:** Okay. Okay. Thank you. So, you know, are there any general principles or procedures that can be used in risk pricing?

**Interviewee:** I think, as I said at the beginning, you've got to know what the risk is, understand it, see what information you've got that can help you understand that risk. So, that might be a documentation. It may be like talking about the risk of inflation, talking to people who have that experience, buyers, commercial people. So, try and pull as much information together about that risk as you can. So, you understand it, then value that risk in terms of... you know, for inflation, it might be, well, steel is gonna go up dramatically. So, steel I'm gonna allow, you know, 50,000 pounds. Concrete might not be going to go up as much as that. So, that's only 5,000 pounds. So, value the risk and then at the end of it, say, what is the probability? So, it may be that each one of those items of steel, concrete formwork, fill material, they may have a lot of different probability. So. It may be the really high probability that steel is gonna stay expensive.

And China is one of the reasons for that. We understand that China is going through a boom of building. And so, because of scrap metal going for China building that affects our prices. The United States, we understand is gonna build its way out of the COVID recession. So, they're using lots of steel. Well, there's only some whoosh steel in the world. So, we, you know, we have to take into account that price of that gonna be more expensive to get it. So, it's not just a local thing. So many... sometimes it's a world economy that affects it. And when you put all that together, you then have to put a percentage to it. And that then is the final figures that you're going to put in the job to allow for risk.

And the certainty is you're wrong. Certainty on all risks is you wrong. You never can get it right. Some of you've put too much in, some you've put too little in, but your hope as a whole, you've got sufficient money to cover risk because it is a guess. It's not real numbers. It's predicting things in the future that may or may not happen. But that's the sort of process I go through anyways. Understand it, value it, and then look at the probability of it.

**Interviewer:** Oh, okay. Yeah. It seems to be a very, very complex process.

**Interviewee:** It depends. In certain items, like inflation you know, fixing prices is very complicated because as we said, it's not just a local thing. It's a world. You know, steel prices are not, not just to do with UK economy. They're to do with the world economy. Things like our price for stone and materials like that. They are more UK based then they're not really affected by the world economy. We don't import stone from America and things... So, it depends on what it is and, you know, ground condition risk is very local. It's all about the field that you're digging that road in. It's not about anything else than that.

So yeah, it's... as I say, it's ... you have to understand the risk and to understand the risk you've got to get as much information about that risk as you can. And some risks are world risks, and some risks are, you know, no big than the field that you do in the road in really. So, once you've done it, once you understand it, it's easier to value it. And once you've valued it, you can then think about what the percentage, probability of that risk is that. That's always the process I use anyway.

**Interviewer:** Okay. Yeah. Thank you. So, you know for your understanding, you think what skills or knowledge are helpful for you to make these ... make a better guess, make a better risk pricing?

**Interviewee:** Yeah, the most important thing in making the right assessment of a risk is understanding what you know, do you know enough to understand that risk? And if you do, then you're the best person to do it. If you don't know enough to understand that risk, it's found someone who can understand that risk. So, if it was a risk about the ground, I would go to someone, a geotechnical engineer who has expertise in that and I'd use their knowledge to help me make a judgment about that risk.

As we said about the risk of inflation, I know my knowledge isn't good enough. So, I go to someone, the buyers, the commercial people who have that knowledge. So, to me, the most, the first big thing is, do I know enough to make a judgment? And if I don't, I shouldn't do it because it's not a risk. It's a guess. And we shouldn't be putting guesses down. We should be putting calculated risks, not just think of a number and throw it in. That's you know... that way you end up bankrupt in the company, you know? Cuz, you don't know what you're doing.

So, that’d be my first point is understand the risk. And if I can't understand it, I need to ask somebody to help me understand it, geotechnical engineer, or a structural engineer, or a buyer or a commercial manager, whoever it is has got better knowledge then I asked them for that knowledge and I use that then to calculate the risk.

**Interviewer:**  Okay. Okay. Thank you. So, you know, at the beginning of our interview, you mentioned that you classify the risks into the physical ones and the commercial ones. So, you know so for the commercial ones, when you price allowance for it, do you think it's only a financial application or... do I, sorry, do I express clearly? I mean, for example, for the commercial risks where you make an assessment on them, do you only think from the financial perspective or, you know, you will take other things into consideration?

**Interviewee:** It's mostly financial because as an estimator, I am responsible for the price. So, it's mostly financial, but some risks are more about the company's image. Would we want to be seen to ... let's think of something… we're working on a road where gonna close some lanes on that road. And we have a period of time where we can close those lanes and then they've gotta be opened up for traffic again.

And there's a risk that we might not be able to do it. Now that has a financial cost. It costs us more money to keep the traffic management running and there maybe penalties that the client the highways agency might put us and say, you know, 'for every hour that the lane's not open, we're going to charge you you know, 50,000 pounds'. So, that's a big financial risk, but there's also an image risk. That do we want to be in the middle of, you know, London and we've granted the whole of London to a standstill because we've not taken enough... some traffic management. And our name is banners everywhere to [place A], you know, [place B] to you know, to a standstill and we wouldn't not allow that to happen.

So, it may be that we'd look at that risk more as a risk of image, than there's a risk of the cost of it. And if we thought that there was a big risk to our image. You might actually say this risk is too big. I know you can value it. And I know you can say it's worth, you know, 100,000 pounds, but the risk to our image. It's too big and we're not going to do the job, or we're gonna tell the client, if we are gonna do the job, we're gonna do it in a different way. We're not going to do this. So, normally it's about money. It's about financial risk, but sometimes it can be about the company itself and the image that company, once they keep up there and it doesn't want it tarnishing by, you know, we've blocked the M1 for a day or something like that. So, you know, if we're bringing a, putting a bridge over a motorway, we have to close that motorway. And if we think the risk that we aren't gonna be able to open those motorways. Great. We're not touch the job and it's not particularly just the money, it's we don't want to be on the news headlines that night our name in banner, say we closed the M1. It can't open. So, there's not just financial, but mainly it's financial.

**Interviewer:** Okay. Thanks. Thank you for sharing this. So, I think you mentioned something about Monte Carlo at the beginning. So, how do you think of the idea that, you know, maybe one day the estimators they can't be replaced by some algorithms or computer softwares? I mean, maybe they can't calculate the risk allowance automatically.

**Interviewee:**  Well. It's happening. It's happening already. The estimator is is using a lot more technology. Pricing systems are automated. So, you can now have a system where you could draw something in CAT. You could use BIM to bring all that through, into a pricing model that pricing model could have a library of rates, which it uses that library of rates, and it brings it to a price total.

And then in theory, that is right. But that model is only as good is the information that's being put into it. So, if nobody has actually thought about a particular risk, it isn't in there and it won't ever be in there. So, the algorithm or whatever it is, is only as good as the information that's gone in. And my risk perspective is based on 30 years. And eventually that algorithm will be populated and be able to use the same experience, but it will take time for that to do because you can't just put it in straight away. You can look at data, but the data is only about what it knows about.

So, it, always will, in my opinion, it will always need a human to check it, to look at it and sense check it because mathematical algorithms, BIM models, they're only as good as the information that's been put in. And if it doesn't know about something, it can't allow for it. So, it would know this, but if nobody had put into the model about inflation, you wouldn't get any inflation in it. You know, if nobody's put into the model about weather, you won't get any weather allowance. If nobody's putting to the model about ground conditions, you won't get any ground conditions.

So, it's knowing that it's got everything in it and even then, does it understand the fact that this is a high-risk or a low risk? How does it make that judgment? Humans that still have a role to play. You know, mathematic algorithms are really good and, but I still think humans have a role to play. That sometimes things aren't quite the norm, but anyway, you're right. It will. The more we move forward, the more people will put together algorithms and they will become more usable.

But I think for me at the moment it's still sick with me. I wouldn't like to just the machine turn the handle and it says put 100,000 pounds in because I need to know what the risk is, understand the risk, value the risk, and come up with a probability. If the machine doesn't give me that information, I wouldn't be very happy. That's why I was never very happy with Monte Carlo because it gave you a curve and you had to just choose where you were on the curve. And it's like...No. I, you know, I saw how my mind works. My mind works... an individual risk. Then the next risk, then the next risk while Monte-Carlo seems to add them all together, multiply them and I wasn't ever very impressed with it to be honest but well that again, I'm quite old. So, maybe, younger people to be more interested.

But... but yeah, it will work, it will happen because people are expensive. Mathematic algorithms are cheap. You know, you can get somebody to type it. That'll cost you a lot of money, but then you've got it and you use it forever. People cost wages every month. So yeah, the more that industry can automate and use math, use algorithms, use models, then they will because it's cheaper than using people. But it does have a risk in itself in doing that.

**Interviewer:** So, you think it's good to be objective or subjective in risk pricing?

**Interviewee:**  I think you need both. I think the best way to look at risk is to have more than one person, because one person looking at it, looks at it with their own subjectivity, their own history, their own experience. If you have two people look at it, then that's two loads of experience. If you have six people look at it, it's six loads of experience. So, I think you need the whole range of experiences. And I understand my own experience. I know that I am likely to be a conservative in my pricing. And I will find the risks. I'm good at finding risks. It is what I've had to do over the years. I'm not as good at finding opportunities. So, maybe other people will find the opportunities and it's that balance of risk and opportunity. That means that our can actually put a successful tender in really. But...so I, I'm never frightened to say, I need someone else's experience on this. Who's the best person to actually helped me with that, cuz he can't know everything. And so that's... it says I'm quite a conservative, quite pessimistic in a way of looking at risks. I need to be balanced by someone who's more optimistic. And we understand that when we look at risks and we do a meeting about risk. We tend to understand who's optimistic, who's pessimistic. And it's a balance between those views because there isn't a right answer. It's a, it's a judgment at the end of the day.
