# Supplementary material for: Critical factors influencing cost estimators’ judgements on cost contingencies in highway construction projects: An empirical study in the UK
Source: PLoS One. 2024 Dec 16;19(12):e0314665. doi: 10.1371/journal.pone.0314665 (PMC11649144; doi:10.1371/journal.pone.0314665)
Supplement: S2 File — (ZIP) [file pone.0314665.s002.zip › Transcription (Interview K).docx]

**Interview K-Meeting Recording**

**Interviewer:** So, what do you want to say about being a cost estimator and handle risks?

**Interviewee:** Well, from a personal point of view, I suppose I think you've already spoken to one of my colleagues, [name]. I think we... I think when you've been doing a job, as long as we have sort of the same topics of risk, crop up or... you know, job after job. So, you know, as well as some jobs have specific risks... that, that generally the... it's... there's almost a shopping list of the types of risk you're likely to get on the schemes.

So... really, we use a risk register as a...as a sort of formal tool to put down our ideas of where we think the risks lie. And I was just looking at the dictionary definition of risk and one of them was the possibility that something unpleasant or unwelcomed will happen. And usually in construction if something unpleasant or unwelcome happens. It's usually a financial risk we suffer. So, it's sort of, I suppose the assessment of risk is based on the experience of, of an estimator which you can only really get when you've, you know, you've been doing the job for a number of years.

I mean, I, I started my career as on site as an engineer. So, and then I moved, moved into tendering, estimating. When I got fed up with all the bad weather and the... being outside in the winter. So, I think you, you know, that those sorts of things that you learn throughout you... my practical career on site, seeing things gives you a sort of insight into the likely like things happening. But yeah... So, when we're producing a tender as I say, we have a risk register and as we go through the process of pricing the job. We, we fill that in come up with ideas that, you know, we think may affect the job may affect the financial outcome. And we produce that as a basis that we then, as a company we discuss before we submit the bid.

And one person's idea of a risk is not necessarily another person's. If, if, for example, you're talking to a director, who's had a bad experience of a particular thing, their view is tarnished by that, you know, that experience. So, they, you know, I might say, well, I don't think, I don't think this is a high risk. And they say, well, you know, I've, I've had bad experiences. So, you know, you're affected by good and bad experiences. And I think the other thing you have to bear in mind when we're pricing, where we'd normally in competition. So, we're, we're trying to win work and we're up against other contractors. We may think that a job has a high risk, but we have to balance that against wanting to win the work and not put into a ... too much risk onto the job and in a, not, not getting the opportunity to to win the work. So, you know, it's this sort of, it's this sort of a bit of a science, but also also an art I would say. And that that's the sort of. An overview of how we look at risk.

I mean, if you're looking at specifics in terms of what sort of things we're looking for when we're assessing risk I mean, obviously in this country, weather is as a big influence. So, if, if the job is likely to go ahead during the winter there's a lot of excavation. You know, there's obviously a risk there that if it, you know, if it's wet or snowing or freezing, you know, it's going to affect the, the outputs. So, you, you, you tend to, you might price an excavation item assuming all the conditions are in your favor. But then you may, you may, in the rear of the end of the job say, well, what if, what if it rains? What if, you know, cuz we're going, we're gonna do this job in January, February. And we look at the weather conditions for that part of the country. And you can go through weather records. It gave you an indication of what the likelihood is of, of the the weather's going to be in, in, you know, at the time of year, because sometimes you think, well, if it's in the winter it'll be wet, but sometimes it's wet in the summer. So, you know, it, it's a bit of a gamble, really.

Other things that we look at obviously at the moment, material supplies are affected certainly in the UK, because of you're aware of Brexit, the leaving of the EU, and that's had an impact on supply. So, at the moment where one of the big things we look at is material inflation. So, we, we use indices for predicting the cost of increase in materials, which are published. And we use those. So, if the job's going to be said a-year contract we know we're gonna buy materials throughout that year. We'll apply a percentage inflation rates at the material cost. Take that into account.

But there's, there's inflation and then there's the supply problem that we've got at the moment, which is probably more than inflation. So, you look at, you know, this particular time is a big issue, material supply. Cuz if there isn't any, it affects, if you can't get materials for the job, it doesn't matter how much you pay for it. If you can't get them, then you can't finish the job, you know, that's that's the issue at the moment. Hopefully, hopefully things will settle down in the next few months and go back to where it, where we, where we were before. Sorry. Yeah, I'm not I'm rabbiting on you. You, you asked if you, if you want to ask me.

**Interviewer:** Yeah. Yeah. Thank you for sharing these and based on what you have talked about, I like to know that you just mentioned that you know, because you use the subject me, so is it the same as what you do as what you just described?

**Interviewee:** Yeah. I mean... well, initially it’s me or the estimator but then before we submit any job we have a meeting well, we'll have the planner does the program. We do have probably somebody that's going to be doing the job on site and the project manager, project engineer. Then we'll have senior management directors. You have to sign off all our bids. So it's a discussion with with a group of people and we come to a collective decision particularly with risk, because as I said before, it is a bit subjective. One person says something as a high risk and another person, you know, is ... that's a lower risk. So, you have to come to some, you have a discussion and you come to an agreement, that's the team effort.

**Interviewer:** Thank you. I think you just mentioned that people will have different judgment because they have different experience. Do you have any other ideas, you know, why people will have different judgements on the same risk?

**Interviewee:** Well, as I just said, I think a lot of it is about experience. And I mean, I suppose, I suppose I would come to a meeting with the view that I've listed all these things out that could happen and maybe on the cautious. So my risk, my risk money is, you know, high. Then a director may come in and say, well, yeah, I accept that these things are gonna happen, but we've got to be a bit more optimistic, and we'll reduce that, that level. So yeah, it, it's not... there is a science to it, but it's not, you know, it does come down to a gut feeling, I think in the end or, or a group gut feeling, if you like, you know, and the, and the most senior person... we'll have the final final say, I mean, that's how it works. I see my job as highlighting the risk and then it's for the rest of us to decide, you know, how much of that we're prepared to take.

**Interviewer:** Yeah. So, you know you just said that you will have a discussion about, you know, the final judgment. So, for you from what perspective you will argue for it. I mean, support for your own judgment.

**Interviewee:** Well, as I say, I think sometimes you look at, say for example, it's probably not a good example because you tend not to have tower cranes on on road jobs. Let's say you had a ...you were doing some crane [unclear], you lifting the bridging over the road. Obviously one of the factors affecting it, a crane, is wind. One of the things you can do, as I said before, you can go through weather records and look at... I mean, if it was on the coast, so the job, you know, is on the coastal position, assuming there's very windy. You may, you may say, well, you know, I've looked at the data on wind speeds in this particular area. And at this time of year, you know, certain time of year that we're gonna be doing this job, you may lose two days of every week to to high winds where you can't use a crane. So, you try and back up the, the, the risk money that you allow by some some science or, or some investigation. You know, you, you're not just plucking figures out of the air, and you're trying to, you build up a cost with, with with a logical argument of why you've put that cost in. So, yeah, they're not, yeah, we don't just guess it numbers. It's it's we try and apply some, some practical science to it to justify it.

**Interviewer:** Okay. Okay. Thank you. And I think you just mentioned a risk of material supply chain. So, how do you think of the controllability of it? The, controllability, I mean, do you think you can do something to control the risk and if the controllability of it change, do you think it will affect your judgment?

**Interviewee:** Emm... yeah, I think, I mean, one of the things I suppose you can do is you can have you can try and talk to suppliers before you submit a bid and come to some, an agreement that if, if you, you know, if you're successful in the job, then the price will be fixed for a period of time. That's normally something suppliers are interested in because it means they're going to be guaranteed the work. Yes. Just at the moment that that is, is very difficult because nobody's committed to a fixed, fixed price. Because no... because of the volatility. Nobody's prepared to say, yeah, I'll, I'll fix my price for a period of time. I mean, that is one way of trying to control supply... the supply chain. It's just at the moment it's, that's, that's difficult.

**Interviewer:** Okay and you just mentioned, you know, the motivation to win a contract, so sometimes, you know, the judgment. So, for you, do you think the motivation to win a contract will affect your judgment sometimes?

**Interviewee:** Oh, definitely. I mean you know, we, we priced a number of schemes. We're not always interested in all of them. You know, we will because we work for different clients. We get inquiries the tenders and they're not all attractive jobs, you know and if some of the, the reasons for them being an attractive is the fact that they've got high risk. So yeah, I mean, we try and target the schemes that we're really interested in. The problem is if we're interested in it, if it's a good job, everybody else is. So, you know, we all, we all, all the contractors all, all make similar judgments. On the, on the, on the flip side of that, you know, you... you can look at a job when you can say, well, I don't think anybody else will be interested, so we can put a higher risk value to it, and we might still be successful. But we don't need be interested in doing it if we had, you know, we'd covered the risk element.

So, you know, it's... tendering is a bit of an art really. It's... you know, you're trying to second guess what other contractors are doing. So yeah, I suppose our, our assessment of risk is, is a lot depends on how keen we are. We might be a bit more optimistic if we're keen on the job, as I say, you can't, you can't always know how it's going to go.

You know, you might, you might go into a a meeting settlement meeting on one job, and everybody says, oh, this is risky. We, you know, it's got a particular problem. And then you go into another job that they're really interested in, 'that's not sort of, you know, we're not so worried about that', you know, yeah, it's this balancing of, of risk and the needs to win work.

**Interviewer:** Oh sorry, because they know you use the subject, we, so is this the same as what you think?

**Interviewee:** well, as I say, I come up with a... My own thoughts, but ultimately, the way the company decide, whether they're interested in, you know, that they're gonna accept what I've, what I've given them. Yeah, it is, it is a way, you know, we do, it's a collective decision. There's not one person that said, this is what we're going to do it, you know, it's based on a collective decision, but the initial costs it down to me or I'm sorry. Yeah.

**Interviewer:** Oh, okay. Thank you. So, in your personal experience and in the contractors' risk, have you ever encountered a risk, you think it's a little bit difficult for you to price it for example, maybe the context is dynamic or complex? Can you take one as the example and talk about how you finally approach it?

**Interviewee:** Well, you, you, yes. I mean, there's always, there's always new problems that that crop up. I mean, there was a scheme we did recently. Well, we just finished it. I mean, we priced it a couple of years ago and the client had... It was a road scheme and it required the volume of fill material. And it was on a... it was next to an old airfield and the client had secured a source of material that was in a stockpile. It was about 12,000 cube of excavated material that had been left in his stockpile. And they wanted to use that as, as, as fill on the job. So, it would save them money because they weren't importing, you know, they weren't buying fill because they'd already got this, this source.

The other risk to us was the... it was only a suitable fill for reuse if the moisture content was... was a certain level. So, because this material had been excavated and left in a stockpile, the chances are that it would have got, got wet and would have rendered it unsuitable. So, the risk to us was could we use that material? Cuz if, if, if it was too wet, we may have to cover the cost of importing a different material. And on that particular job, I think what we decided to do because there was no way of knowing until you tested it and normally when you're in the tendering process, you don't know... Opportunity to test the material. You can only test it when... once you you're on the site. And you know, so the risk was, is that material reusable? So, what we decided to do, we put the extra cost of actually excavating it, but puts it in, spreading it out into areas to allow it to dry. And then we had an... then we'd have enough time... by the time it dried out to reuse it. So that, that was our solution. And I think to be honest, when we got on site, when we did the job, I think it was all right. We didn't need to do that. So that was a sort of risk that we, we allowed for. But we, we, we probably didn't need, you know.

Again, it was, it's one of those, if you're asked, I mean, particularly anything like that, where you're using existing material. For you, you're excavating material that is contaminated say and if it's contaminated, it crosses a lot more to get rid of, to take, to tip. So sometimes you, we, me may ... whatever you like. We'll say, well, if we're digging out a volume, we think 50% of it might be contaminated and that'll be... 50% of that volume will be more expensive than the other 50. Then, sometimes we might go back to the client and say, look, we can't assess. We can't make a judgment because we get borehole information. We're told what the ground conditions are and say, we got 50 boreholes, you know, 50% of them might have shown contamination. But it's very difficult to assess on the job, the... you know, the total problem. And sometimes we go back to the client and say, look, we can't take these risks because we don't know what, we can't quantify it, you know, and, and sometimes we will do that. We just say, sorry, we can't quantify it and they'll come back. And they say, well, well, as a client, they'll take the risk. Although give us more information, you know, it's... that's one of the big, big problems of unknowns.

**Interviewer:** Yeah. Okay. Okay. Thank you for sharing this. So, in this process, do you think there are any general principles or rules can be used?

**Interviewee:** Well, I mean, I suppose the only, the only, the only rule is that anything any unknown or anything that you're unsure about anything you can't quantify directly is, is, is a risk. You know, if, if, if, if you think that you're going to be excavating something that's contaminated and you can't say exactly what it... what it is or how much there is of it that's a risk and, and I don't know about rules that I don't know if you apply rules. I mean, as I, some of the start, you tend to have a list of things in your mind that can go wrong because they've gone wrong before, and you come up with a sort of standard list of things. Then like for example, this, this, the fill material that I refer to is... it was a bit unusual. So, we had to think, think differently about that, but yeah. Yeah. I think the rules are unwritten. I think you… I suppose you do have a list of ideas and things that you're always looking for. Not, it's not written down.

**Interviewer:**  Yeah, I understand what you mean. So, you mean something like rule of thumb?

**Interviewee:**  Yeah.

**Interviewer:** So, would you mind sharing some of yours, rule of thumb... Something like this？

**Interviewee:** oh, crumbs... well, I'm I'm not... a gambling man by by nature. If I think there's a risk, I will always try to quantify it, even if it's having a bit of a guess at what, you know, what the level of the likely that is. I mean, I don't... I think it's far better to try and quantify something rather than, you know just to have a bit of a gut feeling towards something. But yeah, I don't, I'm not sure I don't see it in terms of rules. I just see it as part of the tendering process, you know, like you, you might, you price something, you know... like you know, if there's a pipe drainage trench in pipe being lane between point A and B. You know, we calculate all that we think it's gonna take and what materials we need. Well, the resource, you know, labor resources are required machines that are required and so on. So, we do a sort of analytical, analytical approach to calculate in something that's known and then the unknown. Well, as I say, the material that you're excavating, well, you may hit, you may, you might hit some hard material. It might be concrete or some rock which will slow you down. So, you know, it's yeah, it's a bit of, a bit of just assessing all the information that you're given. And the more information you are gave, surer you are that you can calculate the cost of it. And probably not explain that very well but....

**Interviewer:** Oh no, that's fine. I know something like a gut feeling. It's hard to express. Thank you. Thank you. So, for you you think what knowledge or skill you have, and you think it's helpful for you in risk pricing?

**Interviewee:** Emm... well ... knowledge? Well, the, the knowledge I mean, in terms of understanding, I mean, one of the big issues with, with construction is that all the big unknowns are in the ground. So, you stop digging a hole that's when the, you know, the the uncertainty arises. And so, thing or one scale, if you like, or one area is understanding ground investigation. You know, the client often gives us the ground investigation report. So, a lot of technical information, some of it's useful, some of it discard but it's sort of been able to read those reports and getting an understanding of what the grounds like, the likelihood of at the top of the material that you're gonna be excavating. So, it's a sort of knowledge of, of geology if it was the science of of the soil investigations the certain tests carried out on material that gives an indication how... well odd or soft is. You know, obviously, the types of grounds, whether it's clay, sands, gravels, rock, you know it's just having a sort of an understanding of that sort of science. I'm not an expert. I'm not, I'm not had any formal training in that. That you pick these things up as you go along, you know, you read these reports and you, you get an understanding of what they're telling you.

**Interviewer:** Okay, thank you. So, you know when you price the risk do you think it's always a financial application? I mean, when you, for example, price a risk, do you only think from the financial perspective, or you think it's not?

**Interviewee:** Well, personally, as an estimator, Yeah, everything I do is, is about cost. I mean, one element of a risk, maybe time. So, if, if, if, if we think, you know, it's gonna rain for two months of the year and our program's gonna get pushed back. So that, you know, I will need to talk to the programmer, the planner and say, look, I think we're going to have to add another two weeks to this job. But that have a cost implication, because all our prelim costs, staff costs, temporary accommodation, traffic management, all of those things are priced on a weekly basis. So, by extending the program by a couple of weeks, that will have a cost implication. So yeah, I would say most of my decisions are cost related things.

I don't know if you come across liquidated damages on schemes and the client puts a penalty clause in if you overrun. They'll... it'll cost the contract to so many thousands of pounds a week to as a, as a penalty damages clause. So, you may say, well, we're ganna go over the end day. So, we need to allow that as well, you know, the cost of damages. So, yeah. Yeah. I think, I think estimating is all about cost. Understanding what they, you know, what it's going to cost, whether it's a risk or program or whatever.

**Interviewer:** Okay. Thank you. So, you know, how do you think of the idea that, you know, maybe one day the role of estimator, the risk pricing work can be replaced by some computer software or algorithm? I mean, maybe they can do risk pricing automatically. How do you think of this idea?

**Interviewee:** To be honest with you, I've never come across the idea. It would make my job a lot easier. If we were relying on an algorithm to come up with a cost the only thing is on... I should probably retire in the next couple of years.

So, it, it, it's probably something I'm, I've not come across that was likely to, I would suggest. But is there software out there that, that, that can do this? I don't know. You tell me,

**Interviewer:** no, just, I suppose.

**Interviewee:** Yeah. You're just speculating. I mean, yeah... you can, you can design software and they're all manner of things, but I think whoever, whoever develops an algorithm has got to some point it will be based on people's knowledge and experience, you know? Yeah. I don't know. I suppose if, if, if there was some software that brought all the aspects that we looked at like the weather conditions and the ground conditions and it somehow could bring all that automatically together in an algorithm. Yeah. Yeah. Well, why not? but I don't know if the, about these, these not my... not my field of expertise I'm afraid.

**Interviewer:** Yeah. I'm just curious about your thoughts.

**Interviewee:** Yeah. To add, to be honest, but I don't even really think about it, but I mean, we, yeah. I mean, we use computer software to price jobs, but it doesn't tell us what the price is. It just, it just allows us to bring resources in it's our input that affects the final figure. So, I suspect relying on a machine to do that would be a bit risky, but I think yeah, maybe, maybe it'll come one day. Maybe it's sooner than we think. But personally, I think that would probably be after I've retired.

**Interviewer:** Well, so, what do you think is the difference from you just mentioned you will use a software to price a job. So, what's the difference do you think? I mean, the price given by the software and the price, you know, down by the estimators?

**Interviewee:** Well, yeah, I don't, we, I mean, our software is just a tool. It doesn't tell us what the price is. I mean, we can build standard outputs and constants, or give an... you could sit at the computer with, with our software and you could price the job but that'll give you a basic cost. But then what it doesn't do. What he doesn't understand is the difference, the different conditions that you are likely to find. So, you know, a pipe from A to B or one site in one part of the country might not necessarily be the same price somewhere else. So, that's... the estimator's knowledge and experience to build that in. So, the computer helps, the software helps to get you a basic, you know, to get all your labor, your plant materials into, into a job. But ultimately you have to make judgements on the final figure.

**Interviewer:** Okay. Okay. Thank you. So, for your understanding, you think it's good to be subjective or objective in risk pricing?

**Interviewee:** I think, well, I think you have to be objective thinking... I think the, or as objective as you can be. I mean, sometimes you do, you say, I, I think there's a 10% or a 50% chance of this happening. Yeah, that might be a subjective view. And it might, you might just be producing some of money, a lump of money that you've say, well, I think that will be enough to cover that risk. When you come to that stage, it is a subjective view, but you, hopefully that you've come up with that sum of money.

I mean, there's two aspects. You come up with what the worst-case cost could be. So, this material that was talking about what's going to cost us 50,000 pounds to ... you know, to treat. So that will be the worst case. And they said, well, we can't, you know, we don't think it's going to be that much 50,000, but we think it might be 50% of that. So, we may add 25,000 but it [unclear] ... whether it's 50% or 25% or 75%. Sometimes it's... it’s a bit of a subjective, subjective. Yeah. So, there's an element of it that you try and be objective, I think most of the time, if you can.

**Interviewer:** Oh, so you think it's good to be objective or subjective?

**Interviewee:** Well, I don't know if it's good. I mean, the safest, the safest thing is to be objective. And then if you, if you do end up a bit of, subjective, gut-feeling and nobody can say you're wrong. And sometimes in a, in a group, in a meeting, the group's looking for somebody to say, well, I think it's 50% because nobody knows. And so, my guess is as good as anybody else's. But what... if I say, if, if, if I say, well, I think it's going to be 50%. Everybody got to agree with that figure. because you know, we can't, we can't go to a situation where I've, I've said it's 50%. We got the job, and everybody comes back and says, oh, that's that wasn't enough? I said, well, you know, that was my assessment. My personal view. The time to challenge it is before we put the pricing. But sometimes people like, like somebody to stick their neck out and say, yeah, this is, this is what I think it should be. But I think as a company, we always try and come to a consensus.
