# Supplementary material for: Critical factors influencing cost estimators’ judgements on cost contingencies in highway construction projects: An empirical study in the UK
Source: PLoS One. 2024 Dec 16;19(12):e0314665. doi: 10.1371/journal.pone.0314665 (PMC11649144; doi:10.1371/journal.pone.0314665)
Supplement: S2 File — (ZIP) [file pone.0314665.s002.zip › Transcription (Interview L).docx]

**Interview L-Meeting Recording**

**Interviewer:** How did you become an estimator? I'm quite curious about that.

**Interviewee:** I started out as a bricklayer as, as, and then obviously went to college and got me certificates and I kept on doing my college work and got my H&C and O&C. And then I moved out of the tours into the office, into estimating. That's how I got into estimating.

**Interviewer:** So, you mentioned you go to the college. So, do you learn something for, you know, something relevant to, estimate?

**Interviewee:** No, you picked it up on the way. You, you, you obviously do measurement courses how to do take offs and then the pricing become, well, as far as I was concerned, the pricing became after I got the job, as an assistant estimator. I didn't just go straight into estimating. I did a struction in work training course, literally learning on the job. So, I got into it.

It's not a conventional method of get into estimating. I basically came off the tours and went and went into estimating as an assistant and then aggressively learned over the years.

**Interviewer:**  Oh, okay. So, are you just mentioned that you know... do you mean that company provide some trainings for you? because you just mentioned you have some, on side training?

**Interviewee:**  On site on, on site, on the job. As far as I'm aware, there's not a structured college or university course for estimators to ... They tend to be... they tend to come from other backgrounds and go into estimate. I know you talked to [name]. He's background, he was a site agent before he came into... into estimating. So, all except me at this, tend to be, come from other backgrounds within the construction industry.

**Interviewer:** Oh, okay. Okay. Thank you. So, in your experience in highway projects can you take one risk as an example, and, you know, talk about the things you will consider while you make assessment on it's probably of occurrence and its potential impact on the project?

**Interviewee:** Most of the risks are time related regardless of what they are. Certainly, construction risks are time related. So, all you really do is just try to make an assessment of how much time, if that risk happens is going to add onto the job. So, the obvious one is weather. That you know, if you get in rain, snow, something like that will delay at the job. And so, you have to make it, you try to make an assessment of on which weather will impact the construction of the job. That makes sense?

**Interviewer:** Yes. So how, you measure it, I mean, the probability...

**Interviewee:** Okay. The probability is time of year and looking at historical weather patterns which you should… can get from the Met Office. So, if it's in a winter... if it's in a sort of autumn to springtime, then the probability is going to be high. That you're gonna get delayed on the job. In the wonderful world of estimating it's about one day a week, regardless. They always arrange, one day a week. That is... so then to assess that, to put value to that. It'll be stuff costs and the site costs, which should be cabins, electricity, things like that, any time related costs. So, there's a, there's a rule of thumb on a 2-million-pound job. The weekly on cost will be somewhere between six and eight thousand pound a week. So, if you then evaluate that there's gonna be two weeks delay, then it's two times 8000, 16,000, and then you put a probability against that.

**Interviewer:** Oh, okay. Okay. Thank you. Thank you for, you know, to interpret this. So how, how do you think your, I mean, your personal attitude to risk? Do you think you are the person who are willing to take more risk, or you think you are more conservative about risk?

**Interviewee:**  I think most estimators tend to be conservative. We know that if something goes wrong on-site people get hurt. So, that’s the prime reason that most estimators tend to on the cautious side.

**Interviewer:** So, do you think your risk attitude will affect your risk assessment?

**Interviewee:** No, definitely. Yes. The last thing we want is for people to get hurt and for the job to be delayed. So, we will tend to be on the cautious side rather than I don't know if you're familiar with a gung-ho.

**Interviewer:** Yeah. So, do you, I mean, do the risk assessment by yourself for the project, or do you do that in a team? Something like this?

**Interviewee:** No, we'll be a team, the team collaboration or a team method. Cuz we'll look, we all look at the job and we all try to identify risks. Once it's been identified, then that's usually me or the estimator that would have to put a cost value against that. But no, I don't come up with all the risks myself. It’s a team effort. I just have to group money, how much we should have against it.

**Interviewer:** So, do you, I mean assess the value by yourself?

**Interviewee:** The most of the time, yes. This is the areas where you do check advice especially if you've never come across it before, or too unusual risk. And in civil engineering, most of the construction risks are in the ground that digging holes, that's work support. So, if there's something unusual in that, then you obviously have to take advice on the cost and the delays and the consequences of that happening.

**Interviewer:** Okay. Thank you.

**Interviewee:** So, the other types of risks you get will be commercial risks or contractual risks. There, there will be probably assessed as... at director level then.

**Interviewer:** Oh, okay. So, the risk you assessed and give value to it, is that the final, I mean, the final value you will add to the bid?

**Interviewee:** For want of a better phrase, I put forward my best guess of what it is, and it will be reviewed by my directors. If they think I'm low, they add lots more to it. If they think I'm high, they’ll take some off. So, it's never the estimator's... I know... you know, this is it, take it or leave it. That just doesn't happen.

**Interviewer:** Ok. Thank you. So, how do you think, why you and your, for example, the director, why you will have different opinions, different judgments on the same risk?

**Interviewee:** It's just... It's more about experience and familiarity, I think. At the end of the day... most of the time they agree with what I put down. Then on occasions, they'll just see it and "I think you haven't got enough in there. For it, nothing will be more, more like four weeks rather than a three-week delay". That... it's those type of minor deviation, sort of all the opinions. But these are directors, so he is this... takes president.

**Interviewer:** So, when you, I mean, discuss with him, or argue for your own judgment, from which perspective you will do this? I mean, argue for your own judgment.

**Interviewee:** You will... you... it's not... if you feel really strongly about something, then you will argue for it. But for most of the time, it is done to the director's final sealer not.

**Interviewer:** Oh, okay. Okay. Okay. Thank you. So, you just mentioned experience, so can you be more specific? I may for you, you think what your experience, you know, are useful, helpful in your risk pricing?

**Interviewee:** Again, to put it simply, I'm a... Dig a hole and throw some concrete in it. But if it comes across a tunnel for instance, then other people have more experience they're digging tunnels than I have. So, you would have to bow to their better understanding and experience of the job... so... of the risk. It's just... there's no real formula to it. It is just about experience and feeling and motion of the job.

If... If you're digging a hole in rock that there's very little risk attached to that, but if you're digging it in wet sand, then there's a lot of risks to it. The risk would be collapse, what would be the hole or the trench collapsing. And if there's somebody in that, you know, you could ... could kill somebody, if you haven't got... if you haven't properly, properly assessed the risks of that. So, safety is, right at the heart of any risk assessments that we can do.

**Interviewer:** Okay. Okay. Do you mean when you price a risk, do you think it's always a financial application?

**Interviewee:** Yeah, that’s really what it is. We knew all the dangers and we put a lot of the safety element into the cost... it's all over and above that, if something happens, something that you, you can't really foresee our price for. You... get... if it rains persistently for two weeks, nonstop. Then that's gonna really impact on the ground. It's water seeping in and you can actually undermine the works that you've done, even the temporary works. So, it isn't just mainly a cost implication of what we've already assessed is necessarily to do the works. That make sense? To over and above, what we've already allowed for within the direct costs.

**Interviewer:** Yeah. So, I mean, for you when you price the risk, do you only thinking from the financial perspective or I mean, will you only think about its financial impact, or you will think from other perspective as well?

**Interviewee:** Yeah, ultimately it does all come down to finance, but that's not, that's not the main driver. The main driver is, is... it's unanticipated, what can stop the job? And what can cause... what can be caused damage to the works. Ultimately, whatever the risk is, you have to put a cost against it, but that, but that is not the driver of the risk. The risk is about how likely is it to happen and if it does happen then... then what's the financial ... the financial cost to that event happening?

The protocol ... hand in hand... It's... it's... you can't... I don't think you can separate the cost from, from the rest of the works, foreseen know this. [Unclear] I'm an estimator and that's what I have to do. Just put a cost to some put a cost to something, whether it's a risk or, well, the actual pricing of the jobs. I still have to think about how I'm gonna build it. You can't... you can't just focus on cost that you can't really see it. The cost is the driver. It's... it's the finance… It's the impact of a risk happening. That's what is, is the driver.

**Interviewer:** Okay. Yeah. Yeah. I understand what you mean. Yeah. Thank you for sharing. So, you know, in your past experience, in highway, projects and in the contractors' risk, have you ever encountered a risk, which you feel a little bit difficult for you to price it to? I mean, to price the allowance.

**Interviewee:** Okay. Yeah, you... you do get risks at times where you just cannot evaluate it or put a cost against it. In those circumstances, what we would do, would be to write it out of the contract, the client's risk, not the contractor's risk.

There are risks where I don't know if you've ever heard the term before a tombstone risk.

**Interviewer:**  Sorry, no.

**Interviewee:** It just means if that risk ever happened, then the project would just go. Yeah, the company will probably be out of business because of the financial impact of that risk happening. Most of the... I think I've only ever come across two jobs where we actually had that kind of catastrophic risk that we... the company walked away from in the end. They just, they just wouldn't take it. Okay.

**Interviewer:** Yeah. So, I mean for the risk, the contractors' risk, which you undertake. And I mean, it's difficult but not that difficult. It may because the context of the project is dynamic or complex, or maybe there are some technical or design challenging, something like this, and it make you feel a little bit difficult to price it.

**Interviewee:** Yes. In those circumstances you would, you would talk it over with your line managers and your, your directors. And their attitude is if you can't assess the risk, you pass it on to the client. You, you make it a client risk, not, not a contractor's risk.

**Interviewer:** Oh, okay. Okay. Okay. Thank you. I understand. So now in risk pricing are there any general rules or principles you think that can be followed in the every project maybe?

**Interviewee:** Certain risks happen in every job I mentioned, one was the weather, another one would be soft spots where the ground really soft. So, you can't actually build anything on it. So, you have to dig out the soft spots and replace, place them with the soil with Either a better soil or some, or some granular field, but until you actually start digging, you, you don't know what's there. So, you just make an assessment to see, that 10% of the excavation will be soft spots, which have to be excavated, cave it away and replaced. So, they are all general risks that you... but they are a formula for... and this is ... and they vary from company to company about what kind of percentage you, you put, you put to that... those genetic risks to a job.

**Interviewer:** So, where the percentage come from?

**Interviewee:** That's more historical. From again, from experience of feedback from sites of jobs have been completed, and then they will see. A poll, right? We add this amount of this and why amount of that? And you do tend to get a picture from other jobs all the time, the stool, a percentage on likelihood of that risk happening.

**Interviewer:** Oh, okay. Thank you. Yeah. So, for you, you think what knowledge or skills are useful or helpful for you in your risk pricing?

**Interviewee:** Well, being able to do the job yourself is always good. And it really is just a lot of experience on site what you'd come across in your own experiences, listening to what other people's experiences as well. For them, cuz... things that you haven't thought of other people will tell you that is... Will happen. Whether you agree with them or not is another thing, but yeah... you do have to have a lot of experience and to communicate with, with your other colleagues that over activities, or job... risks that they have come across as well.

**Interviewer:** Okay. Okay. Thank you. So, I'm curious, you know, how do you think of the idea that... sorry, let me firstly ask, does your company use any software to help you do the risk pricing?

**Interviewee:** The short answer is no; I've come across software was it the [list different software] I've come across all those in the past... was can... I don't think they are any better or any worse than just pulling the figure out the air. Really all they... all these software packages, they only work on percentages of historical jobs that... They're useful at… we're getting some money in, but if you're ask the question, why did you do that? You just see oh, them, that's what the machine told me. That would not go down well, with my mind, my directors. They wanted a bit the thought all behind it to help you with that. So, as a room [his company's name], but certainly in the infrastructure side that [his company's name], we don't have that kind of software. It is just solely down to the estimator or the bid team that comes up with the risks. And then the estimate... we'll come up with a price to those risks.

**Interviewer:** Oh, yeah. Okay. Thank you. So, do you think it's possible maybe in future one day there are some software or algorithm they can, I mean, price the risks allowances automatically, and maybe we do not need estimator anymore? Do you think that's possible?

**Interviewee:** You probably do with generic risks. As specific risks to a job, I don't think the technology would be there to be able to price it automatically. Again, gone from my experience of using these electronic versions. They're too generic, to look at a specific risk. Okay.

**Interviewer:** Yeah. Okay. Thank you. So how do you think the risk pricing process? Is it better to be subjective or objective?

**Interviewee:** Both. You know, objective, you identify it. Pricing is very much subjective. So, to your own opinions. So, once the risk has been identified, then you have to be subjective with it and be realistic in assessing it. Is that makes sense?

**Interviewer:** Yes, I know it is... I think the reality, but for your opinions, you think the risk pricing process it's better to be subjective or objective? Yeah. Maybe I can explain this in another way. Because, you know, while people involved in risk pricing job, it seems that the process will become subjective. So, what is the advantage of involving human beings, involving estimators in risk pricing?

**Interviewee:** Well, again, it's just against the technology assessment?

**Interviewer:** Yes, yes, yes.

**Interviewee:** I don't think a computer, or a technology-based program can properly assess a risk. Just one do... just one do that. It doesn't know whether... You could tell it that there’s trench was 10 meters in the air, and they would accept that, whereas everybody knows that the trenches are in the ground. A machine would not be able to do that. And if you told it, it would just throw a number out there. It wouldn't mean anything. So... so I don't believe that a computer program can objectively assess a risk. It needs people to... to put the money in ... it can crunch the numbers, but the people will need to put the parameters into that machine for it... for it to chew out a number or cost. So, I don't think you could... I don't think a computer could ever assess risks and not physical risks, might be to assess a financial risk. With the physical risks, I don't think they would ever be able to do.

**Interviewer:**  Okay. Okay. Thank you. Thank you for sharing these. So yeah, I think that's all I like to ask today. Do you think there are something you think are important, but I left out in this process? And do you want to add?

**Interviewee:**  Right. The whole risks thing is just subjective. And... You can get overly involved in trying to think of every risk there is. And your risk register then just becomes far, far too big. Most of the risks are not really risks anyway. They shouldn't be in the costs as the work. Do you understand direct cost or?

**Interviewer:** Yes.

**Interviewee:** And you do get people at times try and to call something a risk when it isn't the risk. The basic one is if there's a hundred percent chance of it happening, then it's not a risk. So, that has to go into the cost, and you do get people at times that that still want to put money in or put the risk... into a risk that... that shouldn't be in the risk register. So, you, you do get a lot of that at times.

**Interviewer:** Okay. Okay. Thank you. I actually, I have a question, but I don't know how to, how to express it. I mean, for example, there is the risk and for you, for the company, it seems to be a... mostly a financial one, but actually they may have some impact on the, I mean, other stakeholders, but that's not directly related to your financial cost. So, will you take those I mean, take those things while you make risk pricing?

**Interviewee:** There's a ... a sort of contractual third-party risks.

**Interviewer:** Something like, for example, people who live around.

**Interviewee:** Right. So yeah, we get them all the time. An example of that would be a dumper for driving up and down the road. And the people complain that, oh, that dumper was damaging my pavement or damaging my driveway. So, yeah, so you have to have those type of risks in that as well. They might complain about noise and that could stop the job as well. So yes, third parties' risks are more commercial than construction risks, but not should in physical construction risks.

**Interviewer:** Oh, okay. Yeah. Okay. Thank you. Thank you. Yeah, I think that's all I like to ask today.

**Interviewee:** Okay. I don't know whether is... When you're talking about risk. It is... it is about perception of well, what can delay or what can cost you that you can't anticipate? So, my company doesn't like me using the word guests, but that's where it really is.

**Interviewer:**  So, how they describe it?

**Interviewee:** It is assessment. They can use some, woolly words... do evaluate... unknown... put a monetary cost against it. There's some type… type of a science behind the number, can't just say "oh, that looks at about 10,000 pounds of risk". They wouldn't accept that. There would have to have some kind of science and build up behind that number. So, you could, so you can [unclear].

**Interviewer:** So, do you think there are some sciences behind this? I mean, risk pricing.

**Interviewee:**  Yes, there is. As I said probably right at the beginning, it is... it's... most risks are about delay and, and you can assess the delay on a weekly or a daily basis. You've got to do this work, give some kind of science behind the length of the duration of the delay.

As we said, if it... usually it's round and round about, you had the 6,000 pound a week. If the delay is gonna be four weeks, it's 24,000. So, so there's that kind of science behind it.

**Interviewer:** So, is the science always, right? I mean in different projects.

**Interviewee:** Oh no, you can get it wrong. Yeah. But at least you made an effort. You... you've got the, you've got to... you've got to put something there, whether it’s enough or not enough. You never know until it happens.

**Interviewer:** Yeah. Yeah. Yeah. I agree with you.

**Interviewee:** Yeah. That's about it. Yeah. Risk is just about the perception of the delay. Something... cuz nothing in this world happens perfectly. So, there's always gonna be some kind of delay or unforeseen event that you come across. Make it fine... Make an assessment of the likelihood of that event. If it happens, a lot... lot of risks that we do price and never happen.

**Interviewer:** Yeah, but you have to priced it.

**Interviewee:** Yeah. You have to price it cuz the likelihood of that happen. Again, that’s a risk on a risk.

**Interviewer:**  Because I know some... Because for example… even one risk happened, maybe, you know, all the profit are gone.

**Interviewee:**  Yeah. One event can wipe that out if it happens. Yeah. There's a lot of risks that we do prize that never happened.

**Interviewer:** That's a good thing, but yeah...

**Interviewee:** you have to make... you have to make the assessment that if it did.
